# Cooperative CCL2/CCR2 and HGF/MET signaling enhances breast cancer growth and invasion associated with metabolic reprogramming

**DOI:** 10.1080/15384047.2025.2535824

**Published:** 2025-07-30

**Authors:** Wei Fang, Yuuka Kozai, Diana S. Acevedo, Rebecca Brodine, Haasini S. Gorrepati, Nizhoni Arviso, Paige Cote, Alala Thompson, Zachary Gerdes, Ashley Espinoza, Nick Bergeron, Audrey Brownfield, Nikki Cheng

**Affiliations:** a Department of Pathology and Laboratory Medicine, University of Kansas Medical Center, Kansas City, KS, USA; b Department of Cancer Biology, University of Kansas Medical Center, Kansas City, KS, USA

**Keywords:** CCL2, HGF, chemokine, CCR2, MET, metabolism, DCIS, breast cancer

## Abstract

With over 60,000 cases diagnosed in women annually, ductal carcinoma in situ (DCIS) is the most common form of pre-invasive breast cancer in the US. Despite standardized therapy, under-treatment and over-treatment are prevailing concerns. By understanding the mechanisms regulating DCIS progression, we may develop tailored strategies to improve treatment. CCL2/CCR2 and HGF/MET signaling pathways are upregulated in breast cancers. Our studies indicate that these pathways cooperate to promote DCIS progression and metabolism. DCIS and IDC tissues were immunostained for CCL2 and HGF expression. DCIS.com and HCC1937 cells were analyzed for cell proliferation through PCNA immunostaining, apoptosis through cleaved caspase-3 immunostaining, and invasion through Matrigel transwell assays. AKT, AMPK, p42/44MAPK and PKC activities were analyzed in vitro through immunoblot and pharmacologic inhibition. CCL2 and HGF-mediated metabolism were analyzed by LC-MS. Glucose uptake and lactate production were measured biochemically. CCR2 and MET were targeted in breast xenografts through CCR2 knockout and treatment with Merestinib. Significant associations between CCL2 and HGF were detected in DCIS and IDC tissues. CCL2 and HGF co-treatment enhanced breast cancer cell growth, survival, and invasiveness over individual CCL2 or HGF treatment. These CCL2/HGF-mediated phenotypes were associated with metabolic changes including glycolysis and increased AKT, AMPK, p42/44MAPK and PKC signaling. CCL2/HGF-mediated glycolysis was reduced with AKT, AMPK and p42/44MAPK inhibition. CCR2 knockout combined with Merestinib treatment inhibited growth, survival, and stromal reactivity of breast xenografts more than CCR2 or MET targeting alone. CCL2/CCR2 and HGF/MET cooperate to enhance breast cancer progression and metabolic reprogramming.

## Introduction

With over 60,000 cases per year, ductal carcinoma in situ (DCIS) is the most common form of pre-invasive breast cancer diagnosed in women in the US.^1^ DCIS is characterized by the growth of carcinoma cells confined within the breast ducts. It is considered the immediate precursor of invasive breast ductal carcinomas (IDC), characterized by the presence of breast cancer cells in the stroma. Standard DCIS treatment involves a combination of lumpectomy and radiation therapy, along with anti-hormonal therapy for DCIS cases that are positive for estrogen receptor (ER) and/or progesterone receptor (PR). Despite an established treatment regimen, up to 20% of DCIS patients experience disease recurrence, with half of these cases accompanied by IDC. As such, standard treatments are not effective for a subset of patients. Conversely, most cases of DCIS cases are not expected to progress to IDC, resulting in overtreatment and reduced quality of life for many patients.^2,3^ Currently, there are no reliable approaches to predict which cases of DCIS will become invasive. Prognostic factors commonly used to assess IDC hold a different significance for DCIS. Small- or low-grade DCIS lesions may still become invasive. Basal-like breast cancers are considered the most aggressive subtype for IDC, but not for DCIS.^2,4^ Increased expression of biomarkers such as COX2, FOXA1, HER2, Ki-67, p16/INK4A, PR, and SIAH2 are associated with higher risk of recurrence for a subset of DCIS cases but do not predict invasiveness.^2,5,6^ By understanding the mechanisms that regulate DCIS progression, we may develop more effective treatments to reduce under- and over-treatment.

The breast is made up of mammary glands surrounded by connective tissues. The mammary gland is made up of milk producing lobules and ducts that carry milk to the nipple.^7^ The function of normal mammalian cells requires the balance of energy production and nutrient consumption. During aerobic glycolysis, the uptake and metabolism of glucose results in the production of pyruvate, in which its conversion to acetyl CoA drives the TCA cycle. The TCA cycle fuels mitochondrial activity, including oxidative phosphorylation to generate ATP and production of reactive oxygen species. Glycolysis and the TCA cycle provide intermediates necessary for metabolic pathways involved in the synthesis of fatty acids, amino acids, and nucleotides.^8^ Metabolic reprogramming is an important hallmark of cancer and is partly characterized by elevated glycolysis and glucose conversion to lactate even under aerobic conditions (Warburg effect). In late-stage breast cancer, lactate contributes to tumor growth and metastasis and modulates immune response in the microenvironment. Lactate also promotes tamoxifen resistance in hormone receptor-positive breast cancers through AKT and c-MYC dependent mechanisms.^9–11^ Recent studies show that expression of glycolytic enzymes is upregulated in DCIS and IDC tissues, indicating that metabolic reprogramming occurs in early-stage breast cancer progression.^12,13^ Currently, it remains poorly understood what metabolic changes occur during early-stage disease progression and how these metabolic changes are regulated in breast cancer. Our studies indicate that these processes are regulated through cooperation between receptor tyrosine kinases (RTKs) and G protein coupled receptors (GPCRs).

RTKs are a class of transmembrane cell surface proteins that includes a cytoplasmic tyrosine kinase domain that is activated upon extracellular binding of ligands such as growth factors.^14^ In breast, MET RTKs are important for mammary gland development. MET RTKs are expressed in ductal epithelial cells and bind to soluble hepatocyte growth factors (HGF) expressed in stromal cells to coordinate ductal branching and outgrowth.^7^ HGF binding to MET triggers receptor dimerization, autophosphorylation at Y1234/5 in the activation loop of the kinase domain and transphosphorylation at Y1349 in the C’ terminal domain, which acts as a docking site and recruit signaling effectors to regulate cell proliferation, survival, and scattering.^15^ Overexpression of HGF and MET RTKs have been reported in IDC.^16^ Increased HGF/MET signaling has been implicated in DCIS progression and enhancement of late-stage disease progression, including increased breast tumor growth, metastasis, and therapeutic resistance.^17–19^ While HGF is considered the primary ligand for MET to regulate signaling, recent studies indicate that activity of MET receptors may also be regulated through interactions with GPCRs mediated by chemokines.

Chemokines are small soluble proteins (~8 ka) that form molecular gradients to regulate the homing and trafficking immune cells during inflammation. Chemokines bind to seven transmembrane spanning GPCRs and activate pathways that regulate cell adhesion, proliferation and migration. The chemokine family is categorized in C-C, CXC, CX3C and XC classes depending on the composition of a conserved cysteine motif. C-C chemokines are key regulators of T cell and macrophage recruitment and function.^20^ In particular, the C-C chemokine ligand 2 (CCL2) promotes chemotaxis of monocytes/macrophages to sites of inflammation during wound healing and infection. CCL2 coordinates immune function primarily by signaling to CCR2 and activating PLC, AKT, and MAPK signaling pathways. Chronic dysregulation of CCL2/CCR2 signaling has been implicated in fibrotic and inflammatory diseases including liver and pancreatic fibrosis, inflammatory bowel disease and colitis.^21^ Expression of CCL2 and CCR2 are upregulated in multiple types of carcinomas including prostate, lung, pancreatic and breast cancers.^22,23^ In late stage cancers, CCL2 is best known for its role in regulating the recruitment of macrophages to promote tumor growth and metastasis.^22,23^ In early-stage breast cancer, CCL2/CCR2 signaling to carcinoma cells is an important mechanism for promoting breast cancer growth and invasion.^24–26^ Currently, it remains unclear how CCL2/CCR2 signaling regulates DCIS progression in the presence of other oncogenes.

In recent studies, we demonstrated that CCL2 induces interactions between CCR2 and MET RTKs, independently of HGF expression, to regulate breast cancer cell growth, survival, and invasion.^27^ Although CCL2 and HGF are over-expressed in breast cancers,^16,28^ it remains unclear how CCL2 and HGF function together to regulate breast cancer progression. Here, though analysis of patient biospecimens, in vitro and in vivo models, we demonstrate: 1) associations between CCL2 and HGF expression in breast carcinoma tissues, 2) cooperativity between CCL2 and HGF signaling in regulation of breast cancer cell growth, survival, invasion, and metabolism, and 3) therapeutic effects in animal models of DCIS progression when CCR2 and MET are targeted. Overall, these studies have important implications for the treatment of early-stage breast cancer.

## Results

### HGF and CCL2 co-stimulation enhances breast cancer cell growth, survival and invasion associated with increased p42/44MAPK, PKC, AKT and AMPK signaling

**Figure 1.**
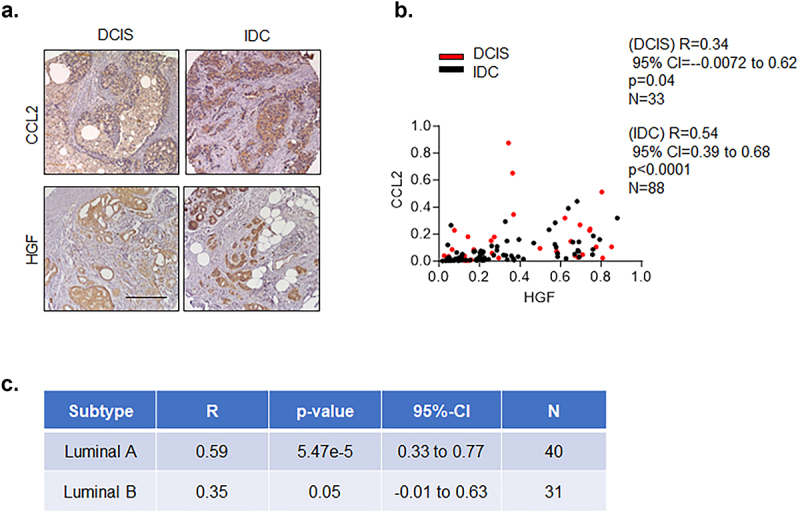
Associations between CCL2 and HGF protein expression in DCIS and IDC tissues. DCIS and IDC tissue microarrays were immunostained for CCL2 or HGF expression. (a). Representative immunostaining is shown. Scale bar = 200 microns. (b–c). Associations between CCL2 and HGF expression in DCIS and IDC were determined by Spearman correlation test. Associations between CCL2 and HGF expression are shown for DCIS and IDC (b) or molecular subtype (c). Statistical significance was defined by *p* < .05.

While CCL2 and HGF expression were shown to be elevated in breast cancer, these studies were conducted independently of each other.^16,28^ Therefore, we sought to determine the associations between CCL2 and HGF expression in DCIS and IDC tissues. mRNA breast cancer datasets contained less than 10 DCIS cases for investigation, as determined through searches of cbioportal.org. Therefore, we assessed CCL2 and HGF expression in DCIS and IDC tissues via immunostaining. To quantify protein expression, we utilized an Image J approach that was previously developed and validated in our laboratory. While the standard clinical approach involves manual scoring of protein expression in tissues, this software approach represented a quantifiable method that provided continuous values for more thorough statistical analysis and was validated in multiple studies.^28–30^ Using this approach to analyze immunostaining, CCL2 and HGF were found to be detected in epithelial and stromal tissues in DCIS and IDC tissues (Figure 1a). Correlation analysis revealed that associations between CCL2 and HGF were stronger in IDC than in DCIS tissues (Figure 1b). We then examined associations between CCL2 and HGF with molecular subtype in IDC cases. The molecular subtype of breast cancers was defined according to clinical criteria established by St. Gallen International Cancer Conference.^31,32^ Briefly, luminal A breast cancers were defined as positive for ER and/or PR and positive or negative for HER2 with a proliferation index of less than 14%. Luminal B breast cancers were defined as positive for hormone receptors and positive or negative for HER2 with a proliferation index of more than 14%. HER2+ breast cancers were defined as hormone receptor negative and positive for HER2. Basal-like breast cancers were defined as negative for hormone receptors and HER2 expression. In this study, the most prevalent cases were luminal A and B subtypes. Compared to the Luminal B subtype, associations between CCL2 and HGF expression were stronger in the Luminal A subtype (Figure 1c). Due to the small sample sizes for basal-like (*n* = 10) and HER2+ breast cancers (*n* = 7), we did not analyze associations between CCL2 and HGF in these subtypes. In summary, these indicated significant associations between CCL2 and HGF in DCIS, which were strengthened in IDC.

**Figure 2.**
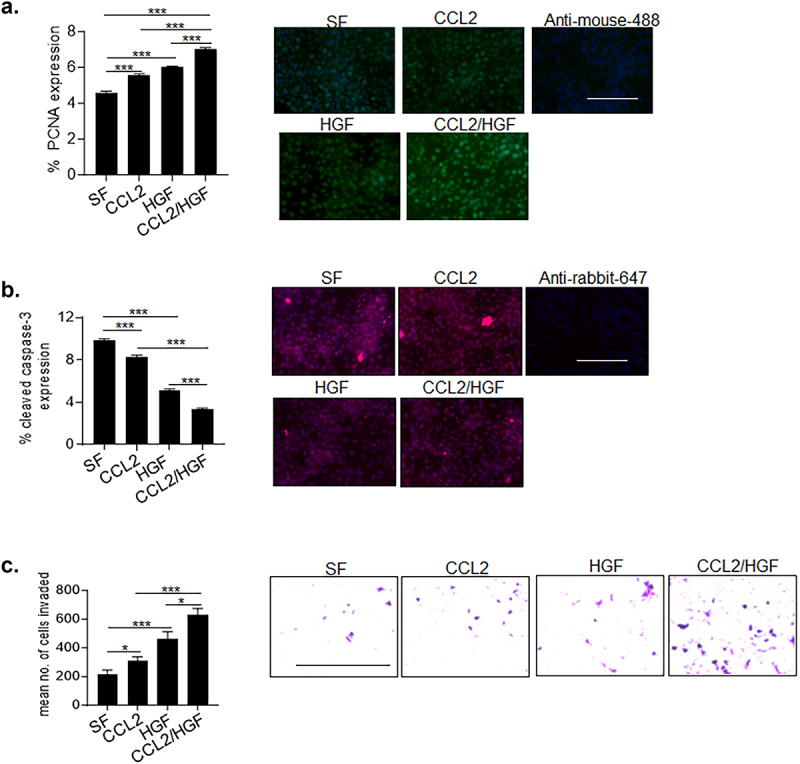
CCL2 and HGF co-stimulation enhances breast cancer cell survival, migration, and invasion associated with increased signaling in DCIS.com cells. DCIS.com breast cancer cells were treated with/without 100 ng/ml CCL2 and/or HGF for up to 24 hours and analyzed for (a). proliferation by PCNA immunostaining (b). Apoptosis by cleaved caspase-3 immunostaining and (c). Transwell invasion assay. Measurements were performed using Image J. Statistical analysis was performed using One way ANOVA with Tukey’s post-hoc test. Statistical significance was determined by *p* < .05. *p < .05, ***p < .001. Scale bar = 200 microns. Mean ± SEM are shown.

Given the associations between CCL2 and HGF expression in breast tissues, we compared the effects of CCL2 and HGF co-treatment with a single treatment on breast cancer cell lines. DCIS.com and HCC1937 cell lines were chosen based on their high levels of CCR2 and MET co-expression.^27^ In DCIS.com cells, co-treatment with CCL2 and HGF resulted in the highest levels of proliferation and invasion as indicated by PCNA immunostaining and Matrigel transwell assays. CCL2/HGF co-treatment also led to the greatest reduction in cellular apoptosis, as indicated by cleaved caspase-3 expression. We noted that HGF treatment alone had a greater effect than CCL2 alone on cell proliferation, apoptosis, and invasion at the concentrations used (Figure 2a–c). In HCC1937 cells, CCL2/HGF co-treatment enhanced proliferation and invasion and inhibited cellular apoptosis the most, compared to single CCL2 or HGF treatment. Similar to DCIS.com cells, HCC1937 cells appeared more responsive to HGF than CCL2 in cell proliferation and apoptosis assays. In contrast, HCC1937 appeared equally responsive to CCL2 and HGF treatment in transwell invasion assays (Supplemental Figures S1a-c). In summary, CCL2 and HGF co-treatment enhanced proliferation, survival, and invasion in DCIS.com and HCC1937 breast cancer cells over CCL2 or HGF alone, with some differences in responsiveness to CCL2 and HGF between the cell lines.

In previous studies, we demonstrated that SUM225 cells were lowly invasive cells that expressed low levels of CCR2. Overexpression of CCR2 in SUM225 (CCR2-H) breast cancer cells increased their invasiveness, correlating with increased MET expression and activity.^27,33^ To determine whether the increased cellular responsiveness of CCL2 and HGF co-treatment were due to levels of CCR2 and MET expression, we compared the activity between pHAGE control cells and SUM225 CCR2-H cells, with or without CCL2 and HGF treatment. In control cells, HGF but not CCL2 alone increased cell proliferation, which was not further affected by CCL2/HGF co-treatment. Neither CCL2 nor HGF affected apoptosis. CCL2 or HGF treatment alone enhanced cellular invasion, which was not further affected by CCL2/HGF co-treatment (Supplemental Figures S2a-c). These data indicated that with low level CCR2 and MET expression, CCL2 and HGF exerted minor effects on SUM225 cells. In CCR2-H cells, CCL2 or HGF alone enhanced cell proliferation, with a minor increase from CCL2/HGF co-treatment. In addition, CCL2/HGF co-treatment significantly reduced apoptosis and invasion over CCL2 or HGF alone. Interestingly, in the absence of recombinant protein, invasion was increased in CCR2-H cells over pHAGE controls, indicating that elevated receptor expression alone possibly increased autocrine signaling to enhance activity of SUM225 breast cancer cells (Supplemental Figures S2a-c). Overall, these data indicate that CCR2 overexpression increases responsiveness to CCL2 and HGF in SUM225 breast cancer cells.

### HGF and CCL2 co-treatment promote alterations in glycolysis associated with nucleotide metabolism

**Figure 3.**
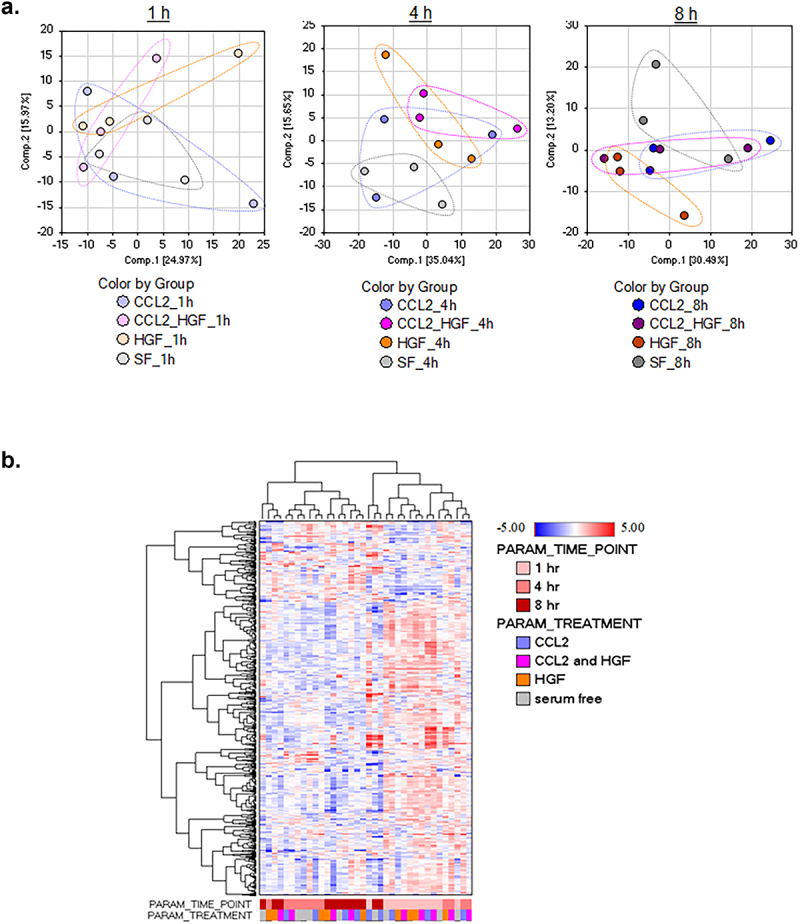
CCL2 and HGF treatment of breast cancer cells alters metabolism over time. DCIS.com breast cancer cells were treated with/without 100 ng/ml CCL2 and/or HGF for 1, 4 or 8 hours and analyzed for metabolite production by LC-MS, *n* = 3/group. Treatments over time were subject to (a). Principal component analysis (b). Hierarchal clustering analysis by Euclidean distance. SF = serum free.

In previous studies, it was shown that CCR2 and MET signaling influenced metabolism in breast and squamous cell carcinoma cells.^27,34^ Given the cooperative effects between CCL2 and HGF on breast cancer cell growth, survival, and invasion, we sought to determine whether CCL2 and HGF also functioned together to regulate metabolism. DCIS.com cells were treated with CCL2 or HGF alone or co-stimulated with CCL2 and HGF for 1, 4 and 8 hours and subject to metabolomics profiling by liquid chromatography/mass spectrometry (LC-MS). Metabolites are summarized in Supplemental Table S1. Principal component analysis and hierarchical clustering analysis revealed that groups clustered according to time and treatment. Treated and untreated groups clustered closer together at 1 hour compared to 4 and 8 hours. At 4 hours, CCL2 treatment group clustered closer to the untreated group, while the HGF and HGF/CCL2 treatment groups clustered together. At 8 hours, CCL2, HGF and CCL2/HGF co-treatment groups clustered closer together over the untreated group (Figure 3a,b). These data revealed alterations in metabolism with CCL2, HGF, and CCL2/HGF co-treatment over time.

**Figure 4.**
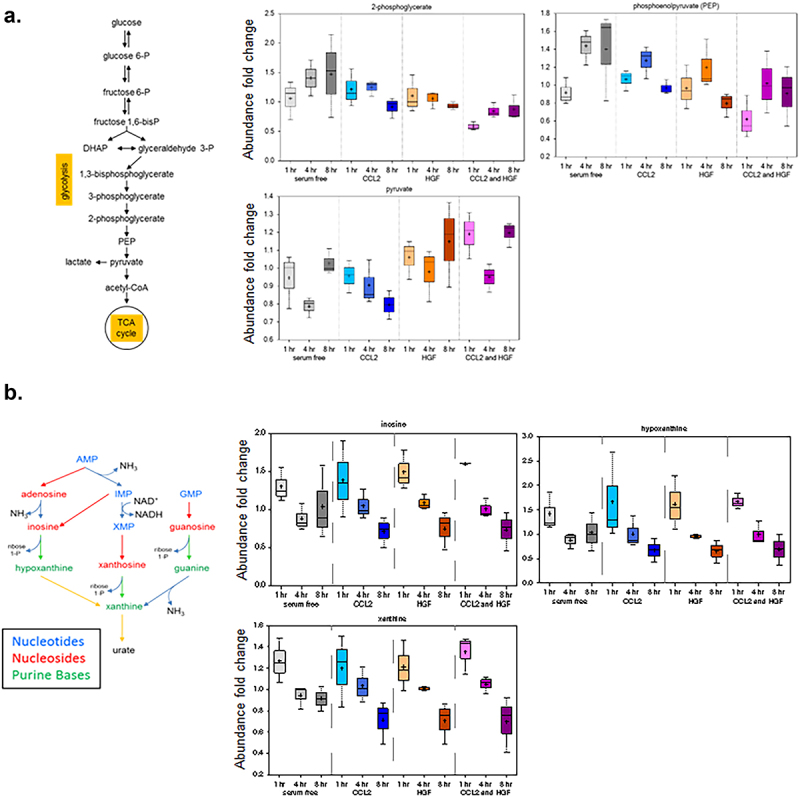
Glucose and nucleotide metabolism pathways are altered by CCL2 and HGF treatment in breast cancer cells. Diagrams and corresponding metabolic intermediates are shown for (a). Glycolysis and (b). nucleotide metabolism. Box plots with upper and lower quartile of median are shown. Error bars represent min and max distribution.

**Figure 5.**
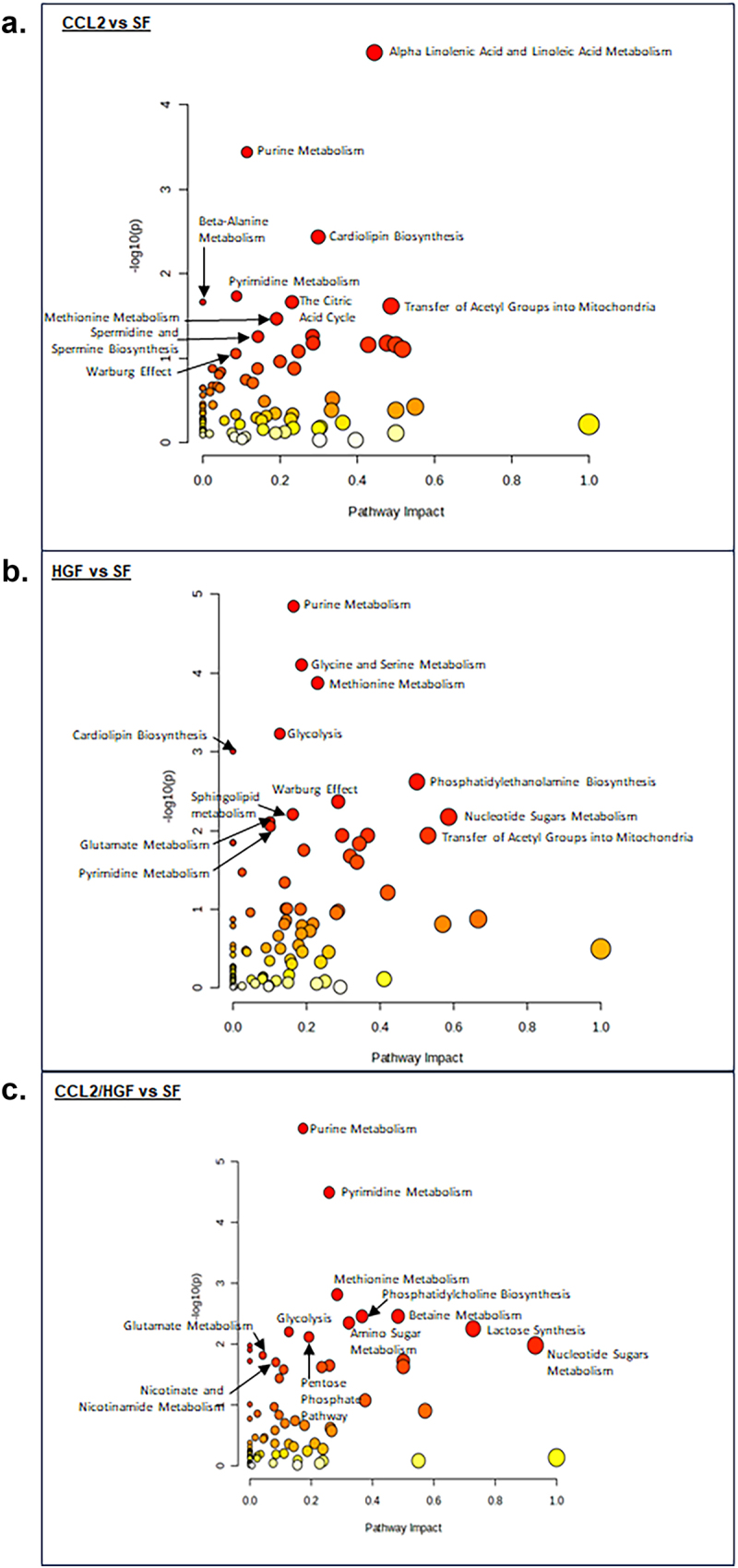
Pathway analysis reveals alterations in distinct metabolic pathways associated with CCL2 and HGF treatment of breast cancer cells. Pathway analysis of metabolites was performed using MetaboAnalyst. Scatter plots with significant pathways are shown for (a). CCL2 vs serum free (SF) (b). HGF vs SF and (c). CCL2/HGF vs SF. Statistical analysis was performed using hypergeometric test. Statistical significance was determined by -log(p-value) < 1.3.

To understand how CCL2 and HGF single and co-treatment affected metabolic pathways, pathway enrichment analyses were conducted using MetaboAnalyst. Pathway analysis revealed overlapping alterations in metabolic pathways with CCL2 single treatment, HGF single treatment and CCL2/HGF co-treatment. Notably, in all treatment groups, there were changes in Glycolysis or the Warburg effect, Pyrimidine Metabolism and Purine Metabolism, as indicated in Figures 4a,b. In addition, CCL2 and HGF single treatment both affected the transfer of acetyl groups into mitochondria. HGF and CCL2/HGF treatment both altered Glutamate metabolism, nucleotide sugars metabolism and methionine metabolism. Despite overlapping pathways, their impact differed among treatment groups. For example, the impact of pyrimidine metabolism was higher with CCL2/HGF co-treatment than in CCL2 or HGF treatment alone. While alterations in glutamine/glutamate have been reported in breast cancers,^35^ this pathway was of lower impact than glucose metabolism in CCL2/HGF co-treated cells. In addition to shifts in the impact of overlapping pathways, there were unique alterations in metabolism among treatment groups. CCL2 treatment was distinctly associated with Beta-Alanine Metabolism, spermidine and spermine biosynthesis, Alpha Linolenic Acid and Linoleic Acid metabolism. CCL2/HGF co-treatment resulted in distinct alterations in nicotinate and nicotinamide metabolism, pentose phosphate pathway and amino sugar metabolism. These pathways are summarized in Figures 5a-c. Overall, these data indicated that CCL2, HGF and CCL2/HGF co-treatment led to changes in overlapping and unique metabolic pathways in breast cancer cells.

As CCL2, HGF and CCL2/HGF treatment of breast cancer cells led to differences in glucose metabolism, we examined for expression of glycolytic proteins over time, including glucose transporter 1 (GLUT1) and rate limiting enzymes HK2, lactate dehydrogenase (LDHA) and pyruvate kinase M (PKM). In DCIS.com cells, compared to untreated cells, CCL2 treatment altered expression of LDHA, HGF treatment altered expression of HK2, while CCL2 and HGF co-treatment altered GLUT1 and HK2 expression (Supplemental Figure S3a). In HCC1937 cells, compared to untreated cells, CCL2 treatment alone altered expression of GLUT1, HK2 and LDHA expression, HGF treatment altered expression of HK2, while CCL2 and HGF co-treatment affected expression of GLUT1, HK2, LDHA and PKM expression (Supplemental Figure S3b). In summary, CCL2/HGF treatment visibly altered expression of glycolytic proteins more than CCL2 or HGF treatment alone, with some differences in expression levels between DCIS.com and HCC1937 cell lines.

### Pharmacologic inhibition of AMPK, PKC, AKT and p42/44MAPK signaling alters CCL2 and HGF mediated glycolysis in breast cancer cells

To determine the intracellular mechanisms associated with CCL2/HGF-mediated cellular activity, we assessed the activity of candidate signaling pathways. Previous studies showed that CCL2/CCR2 mediated p42/44MAPK and PKC signaling was important for breast cancer cell growth, survival, and motility.^25,26^ HGF/MET mediated p42/44MAPK, AMPK and AKT signaling was important for growth, survival and scattering of cervical cancer and head and neck cancer cells.^15,36^ Therefore, DCIS.com and HCC1937 cells were treated with CCL2 and/or HGF and analyzed by immunoblot for phosphorylation of these pathways. In DCIS.com cells, CCL2 and HGF co-treatment enhanced phosphorylation of AKT, AMPK, p42/44MAPK and PKC more than CCL2 or HGF alone. In HCC1937 cells, CCL2 and HGF co-treatment enhanced phosphorylation of p42/44MAPK, AKT and PKC more than CCL2 or HGF alone. On the other hand, AMPK was most highly phosphorylated by HGF compared to CCL2 alone and CCL2/HGF co-treatment. CCL2/HGF co-treatment resulted in AMPK phosphorylation at levels in between CCL2 and HGF treatment alone, indicating that HGF enhancement of AMPK phosphorylation may have been moderated by CCL2 (Supplemental Figures S4a,b). Overall, these data indicated that CCL2/HGF co-treatment enhanced phosphorylation of multiple signaling pathways in DCIS.com cells and HCC1937 cells.

**Figure 6.**
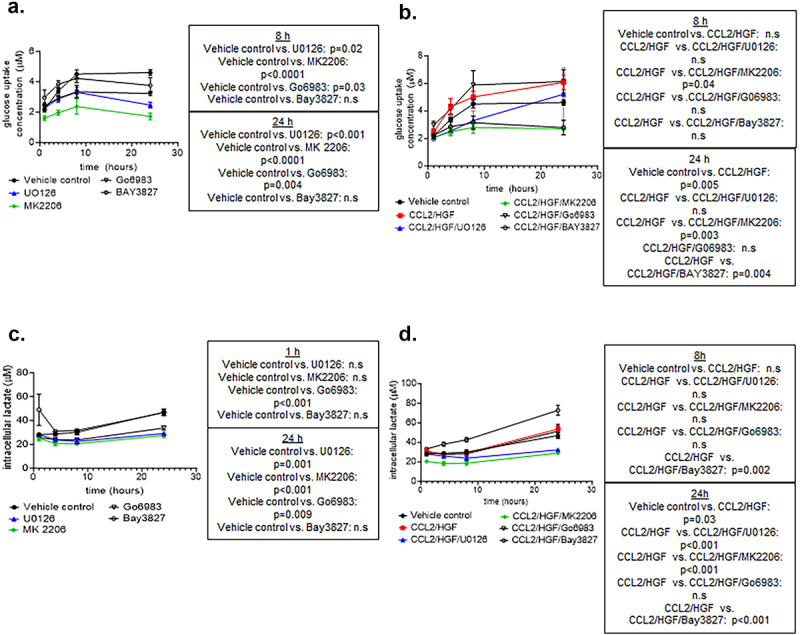
Pharmacologic inhibition of AMPK, AKT, ERK and PKC alters glucose uptake and lactate production mediated by CCL2- and HGF-co-treated cells. DCIS.com cells were treated with 100 ng/ml CCL2 and HGF with/without or inhibitors to AMPK (5 nM BAY3827), PKC (5 nM Go6983), AKT (1 µM MK2206) or p42/44MAPK (1 µM U0126) for up to 24 hours and analyzed for (a-b). Glucose uptake or (c-d). intracellular lactate production through biochemical assays. Statistical analysis was performed using 2 way ANOVA with Tukey’s post-hoc comparison. p-values are shown in boxes next to graphs. Significance was determined by *p* < .05. n.S = not significant. Mean ± SEM are shown.

Given the alterations in AKT, AMPK, p42/44MAPK and PKC signaling with CCL2 and HGF treatment, we determined the contributions of these pathways to CCL2 and HGF-mediated metabolism in breast cancer cells. DCIS.com cells were co-treated with CCL2 and HGF for 1, 4, 8 and 24 hours with/without pharmacologic inhibitors to AMPK (BAY3827),^37^ PKC (Go6983),^38^ AKT (MK2206)^39^ and p4/2/44MAPK (U0126).^40^ DCIS.com cells were examined for changes in glucose uptake and intracellular lactate production through biochemical assays. Basal-level glucose uptake was mediated by PKC, AKT and p42/44MAPK as indicated by treatment with Go6983, MK2206 and UO126 alone (Figure 6a). CCL2/HGF co-treatment increased glucose uptake over time, which was inhibited by MK2206 at 8 and 24 hours and inhibited by BAY3827 at 24 hours. These data indicated that AKT and AMPK signaling were important to CCL2 and HGF-mediated regulation of glucose uptake. In contrast, U0126 or Go6983 did not significantly affect CCL2/HGF-mediated glucose uptake indicating that p42/44MAPK and PKC were not important to this process (Figure 6b). In lactate assays, basal-level lactate production was mediated by PKC, AKT and p42/44MAPK, as indicated by Go6983, MK2206 or UO126 treatment alone (Figure 6c). CCL2/HGF co-treatment increased intracellular lactate levels, which were reduced with MK2206 and UO126 at 24 hours, further elevated by BAY3827 at 8 and 24 hours and were not affected by Go6983 treatment. These data indicated that AKT, p42/44MAPK and AMPK but not PKC were important to CCL2/HGF-mediated intracellular lactate production (Figure 6d). We then examined for changes in the expression of proteins associated with CCL2/HGF-mediated glycolysis, including GLUT1 and HK2. U0126 but not MK2206 treatment reduced CCL2/HGF-mediated GLUT1 and HK2 expression. BAY3827 treatment altered HK2 but not GLUT1 expression in CCL2/HGF-treated cells (Supplemental Figures S5a-c). Taken together, AKT, p42/44MAPK and AMPK signaling are important for CCL2/HGF-mediated glycolysis in breast cancer cells.

### Merestinib treatment combined with CCR2 deficiency resulted in decreased growth and survival of DCIS.com MIND xenografts

**Figure 7.**
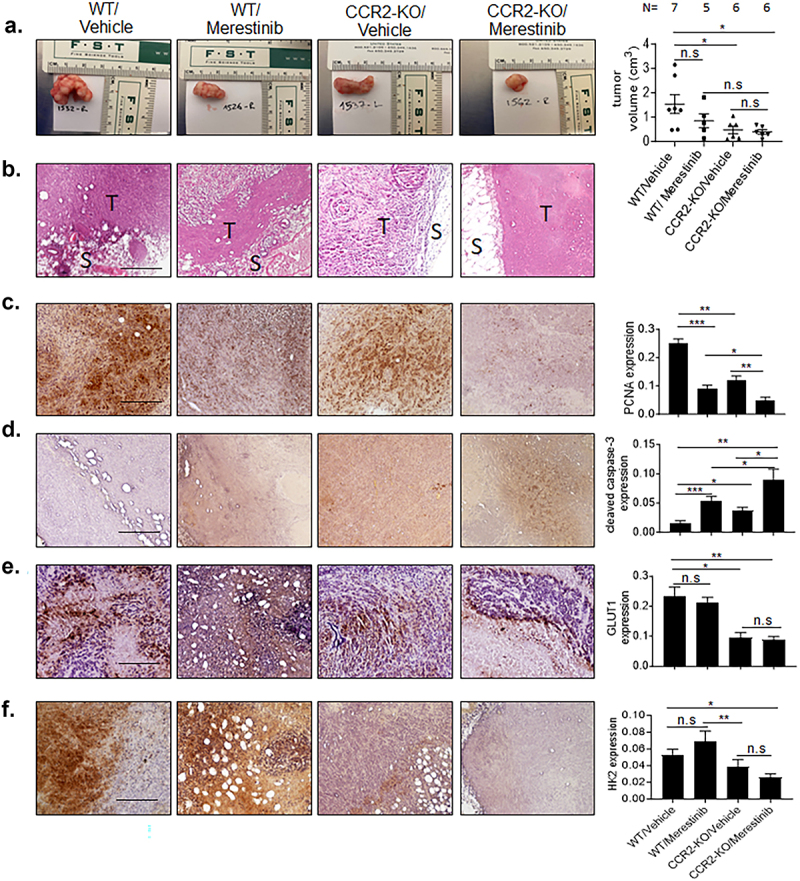
Merestinib treatment of CCR2 deficient breast cancers significantly inhibits growth of DCIS.com xenografts. NSG mice (6–8 weeks old) bearing DCIS.com xenografts with/without CCR2 knockout (KO) were treated with 12 mg/kg Merestinib or 20% captisol vehicle control for 4 weeks. (a). Mammary tissues were analyzed for changes in tumor volume. Representative tumor is shown for each group. (b). Representative H&E stain is shown for each group. Tumor is indicated by T. Stroma is indicated by S. C-F. Mammary tissues were immunostained for: PCNA (c), cleaved caspase-3 (d), GLUT1 (e), HK2 (f). Expression was quantified by image J. Statistical analysis was performed using One way ANOVA with Tukey’s post hoc comparison. Statistical significance was determined by *p* < .05. *p < .05, **p < .01. n.S = not significant. Mean ± SEM are shown. Scale bar = 200 microns.

In previous studies, CCR2 and MET co-expression correlated with the invasiveness of breast cancer cells. In addition, some populations of cells exclusively expressed CCR2 or MET^27^, suggesting variations in CCR2 and MET signaling within breast cancer cell lines. Given the cooperative effects between CCL2 and HGF on breast cancer cells, we hypothesized that targeting CCR2 or MET alone would inhibit DCIS progression more effectively than inhibiting CCR2 or MET alone. Currently, there are few approaches to reliably model the stages of DCIS progression. The Mammary INtraDuctal injection (MIND) model facilitates the investigation of human DCIS progression, by placing the carcinoma cells within the breast duct of animals.^41^ DCIS.com cells that are MIND injected form DCIS lesions that progress to invasive ductal carcinomas over 4 weeks. This disease progression is characterized by initial growth of carcinomas within the breast duct, followed by disappearance of the myoepithelial layer and invasion of breast ductal carcinoma cells into the periductal stroma. The DCIS.com MIND model has been demonstrated to be a reliable and physiologically relevant model of DCIS progression.^33,42^ To target CCR2, mice were MIND injected with CCR2 knockout (CCR2-KO) and compared control DCIS.com cells expressing wildtype CCR2 (WT). To target MET, MIND xenografts received 12 mg/kg of the MET inhibitor, Merestinib (LY2801653),^27^ or vehicle control by oral gavage on a 5/2 schedule for up to 4 weeks. CCR2 and MET dual inhibition was achieved by treating CCR2-KO xenografts with Merestinib. Xenografts were then analyzed for changes in growth and stromal reactivity, which correlates with invasiveness.^43^ Compared to control xenografts, Merestinib treatment did not significantly inhibit the growth of breast xenografts, while CCR2-KO alone significantly reduced the growth of breast xenografts by 68%, consistent with previous studies.^33^ CCR2-KO combined with Merestinib treatment resulted in the most significant inhibition in the growth of breast xenografts, with a 74% reduction in tumor volume. The reductions in growth were associated with decreased stromal and immune reactivity as observed by H&E staining (Figures 7a,b). By immunostaining, CCR2-KO or Merestinib treatment alone inhibited cell proliferation and increased cellular apoptosis, compared to the control group receiving vehicle treatment. Proliferation was further reduced and apoptosis was further increased with CCR2-KO combined with Merestinib treatment (Figures 7c,d). Changes in metabolic biomarker expression were also assessed. GLUT1 and HK2 expression were reduced with CCR2-KO but not Merestinib treatment, compared to the control. Their expression was not further affected by CCR2-KO combined with Merestinib treatment, compared to CCR2-KO (Figures 7e,f). In summary, CCR2-KO combined with Merestinib treatment results in the most significant reduction in growth of breast xenografts and is characterized by reduced carcinoma cell proliferation and survival.

As breast xenografts showed reduced activity in the microenvironment, we analyzed for changes in biomarker expression for fibroblasts (α-SMA and FSP1) and angiogenesis (VWF), and M2 macrophages (arginase-I), which correlates with fibroblast accumulation in breast cancers.^43^ Immunostaining revealed alterations in α-SMA and FSP1 fibroblast biomarker expression among groups. Notably, α-SMA expression was inhibited by CCR2-KO but not Merestinib treatment. CCR2-KO combined with Merestinib treatment did not further affect α-SMA expression, compared to CCR2-KO. These data indicated that CCR2 was a more important factor associated with α-SMA expression in vivo. FSP1, VWF and Arginase I expression were most highly reduced by CCR2-KO combined with Merestinib treatment, with CCR2-KO or Merestinib treatment alone exerting lesser effects on the microenvironment (Supplemental Figures S6a-d). In summary, CCR2-KO combined with Merestinib treatment leads to significant reductions in fibroblast accumulation, angiogenesis and M2 macrophage infiltration.

## Discussion

### Overall summary of findings

In previous studies, we demonstrated interactions between CCR2 and MET receptors in the DCIS progression and metabolism.^27^ In these studies, we showed that HGF and CCL2 cooperated to enhance breast cancer growth, survival, and invasion, induced broad metabolic changes associated with glycolysis, and enhanced p42/44MAPK, AKT and AMPK signaling to regulate glucose metabolism. Finally, we showed that targeting MET and CCR2 in vivo inhibits DCIS progression and stromal and immune reactivity. These studies demonstrate important and novel contributions of CCL2 and HGF to CCR2 and MET signaling, with important therapeutic implications.

### Relevance of CCL2 and HGF co-expression to DCIS and IDC

In previous studies, CCR2 and MET co-expression were more strongly associated with IDC than in DCIS.^27^ Here, we showed that CCL2 associations with HGF were stronger in IDC compared to DCIS. These data potentially indicate that increased CCR2 and MET expression and thus activity may be associated with DCIS progression to IDC. How might CCL2 and HGF expression be regulated? CCL2 and HGF expression are modulated by cytokines such as TGF-β.^44,45^ In breast cancer, CCL2 is regulated at the transcriptional level by EGR1, RXRA and PARP.^44,46^ In addition, CCL2 is regulated post-transcriptionally by miRNAs^47,48^ and long non-coding RNAs.^49^ On the other hand, HGF gene expression is regulated by SP transcription factors^50^ and at the post-transcriptional mechanisms through miR-199a.^45^ These data indicate that CCL2 and HGF are induced by similar cytokines but may be regulated differently downstream at the transcriptional and post-transcriptional levels. Overall, the mechanisms that regulate CCL2 and HGF appear distinct and multi-factorial and could depend on the cancer type. Understanding the specific mechanisms that regulate CCL2 and HGF expression are regulated in breast cancer may enable us to predict when CCR2 and MET signaling may be altered during breast cancer progression. Overall, these studies indicate that increased CCL2/CCR2 and HGF/MET co-signaling represent significant factors associated with DCIS progression.

### Significance of CCL2/HGF-mediated signaling and metabolism in breast cancer cell lines

Here, we showed that CCL2/HGF co-treatment increased cell growth, survival, and invasion in DCIS.com and HCC1937 breast cancer cells over CCL2 or HGF treatment alone. CCL2/HGF-mediated cellular phenotypes were associated with increased AMPK, p42/44MAPK, AKT and PKC signaling, indicating cooperative roles for CCL2 and HGF signaling in breast cancer for both cell lines. Interestingly, there were differences in the contribution of CCL2 and HGF to cellular behavior within and between cell lines. In DCIS.com and HCC1937 cells, HGF had a greater effect than CCL2 on proliferation and survival. The greater effect of HGF on cell behavior was observed with AMPK phosphorylation in HC1937 cells but not in DCIS.com cells. In addition to differences in signaling, there were differences in glycolytic protein expression with CCL2 and HGF treatment. Notably, HGF increased HK2 expression in HCC1937 cells but not in DCIS.com cells. Moreover, CCL2/HGF co-treatment altered expression of more glycolytic proteins in HCC1937 cells compared to DCIS.com cells. What factors might cause these differences in CCL2 and HGF-mediated activity between cell lines? As CCR2 and MET are expressed at similar levels in DCIS.com and HCC1937 cells, receptor expression is not a likely factor.^27^ Rather, proliferation and survival in DCIS.com cells could be influenced by intracellular pathways specific to this cell line that may be sensitive to HGF, including STAT3 and p21 activated kinase.^51^ Another factor could be differences in genomic alterations. DCIS.com cells exhibit PI3K mutations that enhance AKT activity.^52^ In colon cancer, AKT/GSK3B stabilizes B-catenin and positively regulates CCR2 and MET activity.^53,54^ As such, PI3K mutations could indirectly affect CCL2 and HGF signaling in DCIS.com cells. In contrast to DCIS.com cells, HCC1937 cells are BRCA1 defective, which is associated with MET expression,^55,56^ and could thus affect HGF signaling. It would be of interest to conduct further studies on how CCL2 and HGF regulate breast cancer cell activity through genetic and epigenetic mechanisms.

Here, we found that CCR2 expression was an important factor in modulating responsiveness to CCL2 and HGF. The increased survival and invasion of CCR2-H cells with CCL2/HGF co-treatment were similar to phenotypes observed in DCIS.com and HCC1937 cells. CCR2 overexpression in SUM225 cells was shown to increase MET expression,^26,27^ likely contributing to the increased responsiveness to CCL2 and HGF observed here. Unexpectedly, CCR2-H cells did not show the same level of increased growth with CCL2/HGF co-treatment, as observed with DCIS.com and HCC1937 cells. One potential factor contributing to these differences may involve molecular subtype. Breast carcinomas, regardless of subtype, show increased CCR2 expression, compared to normal breast. Yet, basal-like breast cancers and cell lines exhibit higher levels of CCR2 expression, compared to luminal breast cancer cells.^26^ As SUM225 cells express hormone receptors and HER2, other oncogenic pathways, notably ER, HER2, and EGFR may be more important in modulating cell growth than CCL2 or HGF.^51,57^ Interestingly, CCR2 overexpression increased cooperative responses between CCL2 and HGF in enhancing cell survival and invasion compared to control cells. This could indicate that receptor expression determines a certain level of threshold of signaling regulating cellular activity. Previous studies have shown that in T cells, 8000 T cell receptors/cell are needed to induce proliferation.^58^ The increased receptor expression in CCR2-H cells could increase the threshold for CCL2 and HGF signaling, with CCL2 and HGF co-treatment amplifying the signals involved in cell survival and invasion. Further, biochemical studies of breast cancer cell lines from various subtypes could be helpful in clarifying the mechanisms through which CCR2 and MET overexpression regulates CCL2 and HGF signaling.

### Significance of CCL2 and HGF-mediated glucose uptake and lactate production in breast cancer

Here, we demonstrated that CCL2/HGF regulated glycolysis through p42/44MAPK, AKT and AMPK dependent mechanisms. For one, CCL2 and HGF regulated lactate production that was partly dependent on p42/44MAPK. Although CCL2/HGF co-treatment did not affect LDH expression, they could regulate LDH activity through other mechanisms including modulation of metabolic intermediates such as pyruvate, allosteric effectors such as ATP and regulating translational and post-translational modifications of glycolytic enzymes.^59,60^ Interestingly, U0126 treatment did not affect CCL2/HGF-mediated glucose uptake but did transiently affect CCL2/HGF-mediated GLUT1 and HK2 expression. It is possible a short-term reduction in glycolytic protein expression was not sufficient to perturb CCL2/HGF-mediated glucose uptake. In contrast to p42/44MAPK, which was important only for lactate production, AKT was important for both CCL2/HGF-mediated glucose uptake and lactate production. However, AKT-mediated glycolysis was not associated with changes in GLUT1 and HK2 expression in CCL2/HGF co-treated cells, indicating their activity may be regulated through other mechanisms. It is possible that CCL2/HGF could activate AKT to modulate GLUT1 activity through translocation, while HK2 activity could be regulated through phosphorylation and/or glucose binding at the N terminal and C terminal domains.^61–63^ Similar to AKT, AMPK was found to be important for CCL2/HGF-mediated glucose uptake and lactate production. AMPK-mediated glycolysis was associated with changes in HK2 expression, which could be transcriptionally regulated through HIF1α-dependent mechanisms.^64^ AMPK may be tumor suppressive or tumor promoting and may either negatively or positively regulate glycolysis.^65^ Our data suggest that CCL2/HGF activation of AMPK is cancer promoting as it positively regulates glycolysis associated with increased breast cancer cell growth, survival, and invasion. Overall, these data demonstrate that CCL2/HGF regulates various aspects of glycolysis through distinct mechanisms involving p42/44MAPK, AMPK and AKT pathways.

### Potential role for CCL2 and HGF -mediated nucleotide metabolism in breast cancer

Here, we found that CCL2/HGF-mediated glycolysis was shown to be associated with alterations in nucleotide metabolism in breast cancer cells. These findings are consistent with previous studies indicating that glucose metabolism regulates purine and pyrimidine metabolism through the PPP and serine biosynthesis pathways and via the TCA cycle.^66^ How might nucleotide metabolism be important in CCL2/HGF-mediated DCIS progression? Nucleotide metabolism is not only important for DNA/RNA synthesis and cell division but also maintains the mesenchymal state of epithelial cells.^66,67^ In breast cancer, increased expression of pyrimidine and purine metabolism associated genes are associated with reduced overall survival and increased immune checkpoint gene expression.^68,69^ Targeting nucleotide metabolism and inhibiting uracil incorporation through targeting dUTPase reduce the growth and survival of invasive breast cancers.^70^ Targeting purine biosynthesis inhibit breast cancer growth and tamoxifen resistance of ER+ cells.^71^ Decreased purine levels shift cells from a proliferative state to a migratory state that is associated with epithelial to mesenchymal transition in A37 and Hela cells.^72^ Based on these studies, CCL2/HGF mediated nucleotide metabolism could be important in breast cancer cell growth, survival, invasion, and alterations in the immune microenvironment to facilitate DCIS progression. It would be of interest to further elucidate the role of CCL2/HGF mediated nucleotide metabolism in functional studies using the MIND model. In addition, metabolic flux analysis would clarify the biochemical contribution of glucose to nucleotide metabolism relative to other metabolic pathways in breast cancer cells.

### Potential relevance and mechanisms of targeting of CCR2 and MET in breast cancer

Here, we showed that targeting of CCR2 and MET inhibited DCIS progression that was associated with changes in growth, survival, changes in glycolytic protein expression and alterations in the microenvironment. Previous studies have shown that CCR2 regulated MET expression and MET expression is important for HGF signaling and CCL2/CCR2 signaling in breast cancer cells.^27^ Therefore, how would targeting CCR2 and MET affect CCL2 and HGF signaling in these studies? First, CCR2-KO or Merestinib treatment alone reduced the growth and survival of mammary carcinomas and inhibited M2 macrophage recruitment, indicating disruptions to CCL2 and HGF signaling with inhibition of CCR2 or MET. Interestingly, targeting of CCR2 and MET resulted in the highest reduction in growth of mammary carcinomas, corresponding to a significant increase in cancer cell apoptosis, decreased cellular proliferation, as well as reduced stromal reactivity and inflammation in the microenvironment. These data indicated possible residual MET expression and activity in CCR2 deficient carcinomas enabled signaling, which was then inhibited by Merestinib treatment. We noted that CCR2-KO alone but not Merestinib treatment alone decreased expression of GLUT1 and inhibited accumulation of α-SMA+ cells, indicating that these phenotypes were regulated by CCR2 independent of MET. It would be of interest to further investigate how CCR2 and MET regulate DCIS progression through codependent and independent mechanisms.

### Significance of CCR2 and MET-mediated alterations in the microenvironment

Here, we observed that CCR2 and MET targeting reduced stromal reactivity and immune infiltration. In particular, CCR2 contributed to decreased α-SMA fibroblast accumulation and tumor angiogenesis, MET contributed to FSP1+ fibroblast and both CCR2 and MET contributed to M2 macrophages. These findings indicated that CCR2 and MET signaling made distinct and overlapping contributions to a cancer promoting microenvironment. How might interactions between breast cancer cells with the microenvironment regulate CCL2/CCR2 and HGF/MET signaling? CCR2 and MET signaling in breast cancer cells may induce expression of factors that regulate accumulation of fibroblasts, macrophages, and endothelial cells such as MMPs, TGF-β. PDGF, CD154 and/or CCL2.^73–75^ Conversely, fibroblasts and macrophages are important sources of CCL2 and HGF,^76,77^ which act on cancer cells to increase CCR2 and MET signaling during DCIS progression. CCL2 may also act on CCR2 expressing endothelial cells to promote angiogenesis or macrophages to induce macrophage recruitment and M2 polarization.^75,78^ Overall, CCL2 and HGF may regulate DCIS progression through complex interactions between cancer cells and the microenvironment and interactions among stromal cells and immune cells.

### Potential utility of targeting CCR2 and MET

While these studies indicate that CCR2 and MET could be therapeutic targets to inhibit DCIS progression to IDC, further studies involving pharmacologic inhibitors must be conducted to validate their utility as targets. At this time, we cannot conclude that pharmacologic inhibition of CCR2 will result in the same changes in breast cancer growth as genetic targeting of CCR2. Targeting both CCR2 and MET may provide some added therapeutic benefit, when compared to single targeting. These benefits include increased cancer cell apoptosis and decreased stromal and immune reactivity, which may suppress long-term growth and disease progression.

Since there are significant associations between CCR2 and MET, their expression as biomarkers could be useful for tailoring treatment in several ways. For one, patients with DCIS, which express high levels of CCR2 and MET, might be considered at increased risk for disease recurrence with invasive progression. While administration of tamoxifen may reduce the risk of recurrence by 50%,^1^ patients with hormone receptor-positive breast cancers might also receive CCR2 and MET targeted therapy to further minimize the risk for recurrence with invasive disease. For patients with HER2+ DCIS, treatment with Trastuzumab modestly reduces risk of disease recurrence.^79^ Resistance to Trastuzumab is associated with MET expression.^80^ Therefore, patients with HER2+ breast cancers might benefit from MET and/or CCR2 targeted therapy to enhance efficacy of Trastuzumab. Patients with basal-like DCIS are typically treated with radiation and/or lumpectomy without a targeted treatment, and may also face a higher risk of disease recurrence.^1,81^ As such, these patients might benefit from CCR2 and MET targeted therapy to reduce this risk. For patients with localized IDC (stage 1 or 2) and high CCR2 and MET expression, CCR2 and MET targeted therapies might be applied in combination with existing targeted therapy and/or chemotherapy, to enhance therapeutic efficacy. To further determine feasibility of these treatment strategies, it would be important to determine the associations of CCR2 and MET expression with breast cancer subtype and disease recurrence in patients with early-stage breast cancer. To translate a feasible strategy to the bedside, it would be important to develop treatment strategies involving CCR2 pharmacologic inhibitors combined with Merestinib and test additional models of DCIS progression. These models could include transgenic models such as the MMTV-PyVmT and MMTV-neu lines, MIND models involving additional breast cancer cell lines and patient-derived xenograft models^2^ to address questions on the evolution of breast cancer subtype, effects of CCR2 and MET signaling in cancer progression over time, effects of CCR2 and MET inhibitors on breast cancer subtype and immune response in early stage breast cancer progression.

### Conclusions

Overall, these studies provide insight into how CCL2 and HGF function to coordinate breast cancer growth, survival, invasion, and metabolism, and demonstrate that targeting CCR2 and MET inhibit DCIS progression. Targeting CCR2 and MET pathways could be effective at preventing formation of IDC and/or therapeutic intervention of IDC. In summary, these findings on CCL2/CCR2 and HGF/MET signaling in early-stage breast cancer could be used to tailor treatments for patients with DCIS.

## Materials and methods

### Ethics statements on human tissues and animal subjects

Tissue microarrays were established from patient samples of DCIS and IDC, as previously described.^28,29^ The tissues were classified as “Exempted” from IRB evaluation as human subjects research and were approved for study by the Human Research Protection Program at the University of Kansas Medical Center (KUMC) (#080193). The Biospecimen Core Repository, a facility approved by the Institutional Review Board, obtained written informed consent for tissue collection and de-identified the samples prior to distribution. Existing medical records were used in compliance with KUMC and National Cancer Institute regulations. These regulations are aligned with the World Medical Association Declaration of Helsinki. Animals were maintained at KUMC in accordance with the Association for Assessment and Accreditation of Laboratory Animal Care. Animal experiments were performed under a protocol approved by the KUMC Institutional Animal Care and Use Committee (protocol number# 25–02–435) and complied with the ARRIVE guidelines.

### Cell culture

DCIS.com cells (also known as MCF10DCIS or MCF10DCIS.com) were derived from a xenograft of the MCF10AT1 breast cancer cell line and were characterized in previous studies to form early-stage breast cancers in vivo.^33,51,82^ DCIS.com cells were cultured in DMEM/10% FBS/2 mM L-glutamine/1% penicillin-streptomycin. DCIS.com CCR2-KO cells were generated by CRISPR and characterized in previous studies.^27,33^ HCC1937 cells were derived from stage IIB breast ductal carcinoma diagnosed in a 23 female.^55^ These cells were cultured in RPMI/10% FBS, 2 mM L-glutamine/1% penicillin-streptomycin. SUM225 pHAGE and SUM225 CCR2-H cells were derived from the SUM225 cell line as shown.^33^ The SUM225 cell line originated from a chest wall recurrence from patient with DCIS.^83^ These cells were cultured in Ham’s F12/10%FBS/5 ug/ml insulin/1 ug/ml hydrocortisone/2 mM L-glutamine/1% penicillin-streptomycin. Cells were tested for mycoplasma infection after thawing using the MycoAlert Plus Kit (Lonza cat no.LT07–701) and were maintained for no longer than 4 months.

### Reagents

Recombinant human HGF (cat no.100-39 H) and human CCL2 (cat no.300–04) was obtained from Peprotech. For in vivo studies, Merestinib was obtained from Eli Lily, Lot no. KW1-E02099-043-A and resuspended in 20% Captisol (Cydex Pharmaceuticals) in water and formulated in 10% PEG 400/90% as previously described. For in vitro studies, the following inhibitors were used: U0126 (LC Laboratories, cat no.U-6770), MK2206 (VWR cat no.101760–616), BAY3827 (MedChem Express cat no.HY-112083), Go6983 (Cayman Chemical Company cat no.13311).

### Immunocytochemistry

Sixty-thousand cells/well in 24 well plates were fixed with 10% neutral formalin buffer, permeabilized with methanol and blocked with PBS/3% FBS for 1 hour. Cells were immunostained with antibodies to: PCNA (BioLegend, cat no.307902) at a 1:100 dilution or cleaved caspase-3 (Asp-175; Cell Signaling Technologies, cat no.9661) at a 1:500 dilution. PCNA expression was detected using secondary donkey anti-mouse Alexa-Fluor568 (Invitrogen, cat no.A10037). Cleaved caspase-3 was detected using secondary donkey anti-rabbit Alexa Fluor647 (Invitrogen, cat no.A-31573). Samples were counterstained with DAPI in 50% glycerol/PBS. Four images/well were captured at 10x magnification using the FL-Auto EVOS imaging system.

### Transwell invasion assay

8 micron pore transwell inserts (Greiner Bio-One, cato.662638) were coated with Growth Factor Reduced Basement Membrane Extract (Fisher Scientific, cat no.343301001) diluted 1:10 in PBS for 1 hour at 37°C. Four hundred-microliter DMEM serum free (SF) medium containing 100 ng/ml CCL2 and/or HGF were pipetted in each well of a 24 well plate. Transwell inserts were placed in each well. Seventy-five thousand cells were plated on top of the transwell in 200 µl SF medium and incubated for 24 hours at 37°C. Cells were aspirated. Transwell inserts were fixed in 10% neutral formalin buffer for 10 minutes and stained with 0.1% crystal violet for 10 minutes, followed by three washes in PBS. The undersides of the transwells were imaged at 10x magnification, 4 fields per well using a Motic AE31 inverted microscope. The number of cells invaded to the transwell underside was quantified by using Image J.

### Immunoblot

For immunoblot, 100,00 cells/well in 24 well plates were lysed in RIPA buffer with protease/phosphatase inhibitors. Lysates were sonicated and debris was pelleted by centrifugation. Protein concentrations were measured by Bradford assay. Thirty-microgram protein was resolved on SDS-PAGE and transferred to nitrocellulose membranes. Membranes were blocked with PBS/0.5% Tween-20 (PBS-T) containing 3% milk for 1 hour and incubated with primary antibodies to GLUT1 (Santa Cruz Biotechnology, cat no. sc -377,228), HK2 (Cell Signaling Technology, cat no.2867), PKM1/2 (Cell Signaling Technology, cat no.3190S), LDHA (Santa Cruz Biotechnology, cat no.sc -137,243), phospho-AMPKα Thr172 (Cell Signaling Technology cat no.2535S), AMPKα (Cell Signaling Technology cat no.5832S), phospho-AKT Ser473 (Cell Signaling Technology cat no.406S), AKT (Cell Signaling Technology cat no.4691S), phospho-p42/44MAPK (Cell Signaling Technology cat no.9101S), p42/44MAPK (Cell Signaling Technology cat no.91202S), pan-phospho-PKC β11 Ser660 (Cell Signaling Technology, cat no.9371S) or Tubulin (Proteintech cat no.66240-Ig). Proteins were detected using appropriate secondary antibodies. Membranes were developed with West Pico ECL chemiluminescent substrate and imaged using a Analytik Jena Imaging System. Densitometry was performed using Image J as described.^26^ Blots were stripped through incubation in Thermo Scientific Restore Plus Western Blot Stripping Buffer (VWR cat no.PI46430) for 15 minutes at room temperature. Blots were then washed in PBS-T three times and blocked for 1 hour before being reblotted.

### Metabolomics profiling and analysis

#### Treatment

50,000 DCIS.com cells were seeded per well in 24-hour plates in triplicate serum starved for 24 hours and incubated in serum free (SF) DMEM with/without 100 ng/ml HGF and 100 ng/ml CCL2 for 1, 4 or 8 hours. Cells were washed in PBS, trypsinized, pelleted and flash frozen in liquid nitrogen. Samples were analyzed in triplicate by LC-MS for 552 metabolites by Metabolon Inc.

#### Bioinformatics

The informatics system consisted of four major components, the Laboratory Information Management System, the data extraction and peak-identification software, data processing tools for QC and compound identification, and a collection of information interpretation and visualization tools for use by data analysts. The hardware and software foundations for these informatics components were the LAN backbone, and a database server running Oracle 10.2.0.1 Enterprise Edition. Raw data was extracted, peak-identified and QC processed using Metabolon’s hardware and software. Peaks were quantified using area-under-the-curve.

Biochemical values were normalized to Bradford concentration and rescaled to set the median equal to 1. Missing values were imputed with the minimum. Principal component analysis was performed in the following manner. The first principal component was computed by determining the coefficients of the metabolites that maximizes the variance of the linear combination. The second component found the coefficients that maximize the variance with the condition that the second component was orthogonal to the first. The total variance was defined as the sum of the variances of the predicted values of each component (the variance is the square of the standard deviation), and for each component, the proportion of the total variance was computed. Hierarchal clustering was performed using Euclidean distance, where each sample was a vector with all the metabolite values.

Metabolites were analyzed in MetaboAnalyst (https://www.metaboanalyst.ca/). Pathway analysis was conducted on metabolites using the Homo sapiens KEGG pathway library.

### Metabolic assays

Ten thousand cells/well in 96 well plates were analyzed for glucose uptake using the Glucose Uptake-Glo Assay (Promega, cat no.J1341), according to manufacturer’s instructions. Intracellular lactate levels were measured using the Lactate-Glow Assay (Promega, cat no.J5021), according to manufacturer’s instructions. Luciferase activity was measured using a Tecan Infinite M Plex plate reader.

### Mammary intra-ductal injection/Merestinib treatment

Non-Obese Diabetic Severe Combined Immunodeficient interleukin 12 receptor- γ2 null female mice, 6–8 weeks old, were purchased from Jackson Laboratories. DCIS.com WT control or CCR2-KO cells (*n* = 5–7/group) were injected through the #4–5 and/or #9–10 mammary nipples using a 30-gauge Hamilton syringe using approaches described.^42^ Animals were monitored for recovery from anesthesia every 5–15 minutes until they were ambulatory. Four weeks later, mice bearing WT or CCR2-KO xenografts were dosed by oral gavage with vehicle control or 12 mg/kg of Merestinib once daily at a 5/2 schedule for 4 weeks. Individual mice were monitored twice a week until endpoint. Animals were not randomized. To demonstrate the robustness of the experiments, batches of mice (*n* = 2–10 at a time) were grafted with cells and treated with Merestinib at different intervals. To reduce bias, treatment order alternated between WT and CCR2-KO groups. Animal experiments were conducted by multiple individuals in the following manner. One investigator (DA) performed the mouse surgery, drug treatments, tissue harvesting and measurements. Other investigators (PC, AE, AB) who were not aware of the experimental groups, performed the immunostaining. Other investigators (NC and ZG) analyzed the data. Animals were included if they underwent successful engraftment of breast cancer cells, defined by the presence of carcinoma as confirmed by histology. The animals were excluded if engraftment was unsuccessful, defined by the lack of carcinoma, which was confirmed by histology. Based on these criteria, six animals were excluded from the study.

### Immunohistochemistry

Tissues were fixed in 10% formalin and embedded in paraffin using procedures previously described.^30^ H&E stain was performed using procedures described previously.^30^ Sections were immunostained for PCNA, cleaved caspase-3, FSP1, VWF and arginase I using procedures described in previous studies.^28,30^ For detection of CCL2, HGF, HK2, GLUT1 and α-SMA expression, 5 micron sections were dewaxed in a series of xylenes and decreasing percentages of ethanol (100–50%). For HGF, HK2, GLUT1 and α-SMA immunostaining, dewaxed sections were subject to antigen retrieval through incubation in 10 mM sodium citrate pH 6.0 for 3 minutes under low pressure in a pressure cooker at 104–105°C. For CCL2, sections were subject to antigen retrieval using 10 mM Tris-EDTA pH 9.0 for 3 minutes under low pressure. Slides were then washed in PBS, subject to peroxidase quenching in PBS/10% methanol/10% H_2_0_2_ for 20 minutes at room temperature. Sections were blocked in Affinipure^TM^ FAB fragment Goat anti-Mouse IgG (H+L) (Jackson Immunoresearch, cat no.115–007–003) or PBS/3% FBS for 1 hour. Sections were then incubated overnight (1:100) with primary antibodies to HGF (Santa Cruz Biotechnology, cat no. 374422), CCL2 (Santa Cruz Biotechnology,cat no. sc -32,771), α-SMA (Spring Biosciences, cat no.M4712), GLUT1 (Santa Cruz Biotechnology, cat no. sc -377,228) or HK2 (Cell Signaling Technology, cat no.2867). Slides were washed in PBS and proteins were detected using appropriate secondary biotinylated antibodies (1:500) for 1 hour in PBS/3% FBS. Secondary antibodies were then bound to streptavidin peroxidases using Vectastain Elite (Vector Laboratories, cat no. PK6200) for 30 minutes. Proteins were detected using 3,3’-diaminobenzidine (DAB) (Vector Laboratories, cat no.SK-4100). Sections were counterstained with Mayer’s hematoxylin, dehydrated and mounted with Cytoseal. Four images at 10x magnification were captured using the EVOS FL Auto imager and quantified by Image J using procedures described.^33^

### Quantification of protein expression

Protein expression in tissues or cells was measured using procedures previously described.^28^ Briefly, images were opened in Adobe Photoshop. Images were normalized to white background. The selection tool was then used to highlight the DAB staining or fluorochrome staining, which was copied and pasted into a new window and saved as a new window. The images were opened in Image J and converted to 8 bit before measuring the particle size. Positive immunostaining was normalized to hematoxylin or DAPI staining.

### Statistical analysis

For animal studies, sample sizes were determined through power analysis using PS: Power and Sample Size software (Vanderbilt University) using tumor mass as the primary outcome. Based on previous studies on Merestinib treatment in vivo,^84^ the response within each subject group was normally distributed with a standard deviation = 110. The true difference in the experimental and control means was determined to be 210. Thus, *n* = 5 experimental subjects and *n* = 5 control subjects were needed to be able to reject the null hypothesis that the population means of the experimental and control groups are equal with probability (power) of 0.8. The Type I error probability associated with this test of this null hypothesis was 0.05.

For metabolomics data, statistical analysis was performed on log transformed values with Array Studio/Jupyter Notebook using One Way ANOVA with p-values adjusted with false discovery rate (FDR) as described.^85^ On MetaboAnalyst, hypergeometric test was used for pathway analysis. For other in vitro experiments, unless otherwise stated in figure legends, assays were conducted with triplicate samples and performed a minimum of three times. Statistical analysis was performed using GraphPad Prism 10 software. Kolmogorov – Smirnov test of normality distribution was performed. For normally distributed data with one factor, the Student’s two-tailed t test was used for 2 groups. For more than 2 groups, data were analyzed using One-way ANOVA with Tukey’s post-hoc comparison or two-factor ANOVA with Tukey’s post-hoc comparison. For data with non-normal distribution, the following tests were used: Spearman correlation test for associations between two variables or Kruskal Wallis Test with Dunn’s post-hoc comparison for more than 2 groups. Statistical significance was defined by *p* < .05. Unless otherwise specified, the statistical significance was indicated as *p < .05, **p < .01, ***p < .001, ns = not significant.

## Supplementary Material

Supplemental Material

Supp figure 4 4 25.pdf

## Data Availability

The authors confirm that the data supporting the findings of this study are available within the article and its supplementary materials. The datasets used and analyzed for this study are available from the corresponding author upon reasonable request.
