# Supplementary material for: Cooperative CCL2/CCR2 and HGF/MET signaling enhances breast cancer growth and invasion associated with metabolic reprogramming
Source: Cancer Biol Ther. 2025 Jul 30;26(1):2535824. doi: 10.1080/15384047.2025.2535824 (PMC12320856; doi:10.1080/15384047.2025.2535824)
Supplement: Supplemental Material [file KCBT_A_2535824_SM9443.docx]

**Supplemental Table 1. Heat map of statistically significant biochemicals profiled in this study.** Red and green shaded cells indicate p≤0.05 (red indicates that the mean values are significantly higher for that comparison; green values significantly lower). Light red and light green shaded cells indicate 0.05<p<0.10 (light red indicates that the mean values trend higher for that comparison; light green values trend lower). For the ANOVA, blue-shaded cells indicate p≤0.05; light blue-shaded cells indicate 0.05<p<0.10

| **Biochemical Name** | **Platform** | **Comp ID** | **HMDB** | **PUBCHEM** | **1h CCL2/SF** | **4h CCL2/SF** | **8h CCL2/SF** | **1h HGF/SF** | **4h HGF/SF** | **8h HGF/SF** | **1h CCL2 HGF/SF** | **4h CCL2 HGF/SF** | **8h CCL2 HGF/SF** |
| --- | --- | --- | --- | --- | --- | --- | --- | --- | --- | --- | --- | --- | --- |
| glycine | LC/MS pos early | 58 | HMDB00123 | 750 | 0.97 | 1.02 | 1.03 | **0.89** | **0.93** | 0.97 | 0.93 | 1.01 |  |
| N-acetylglycine | LC/MS pos early | 27710 | HMDB00532 | 10972 | **0.85** | **0.84** | 0.99 | 0.97 | 0.88 | 0.95 | 1.11 | 0.89 |  |
| betaine | LC/MS pos early | 3141 | HMDB00043 | 247 | 0.94 | 1.01 | 0.91 | 0.92 | 0.91 | **0.85** | 1.04 | 0.94 |  |
| serine | LC/MS pos early | 1648 | HMDB00187 | 5951 | 1.00 | 0.97 | 1.06 | **0.91** | **0.91** | 1.02 | 0.93 | 1.00 |  |
| N-acetylserine | LC/MS pos early | 37076 | HMDB02931 | 65249 | 0.99 | 0.98 | 0.97 | 1.00 | 1.00 | 0.98 | 1.01 | 1.01 |  |
| threonine | LC/MS pos early | 1284 | HMDB00167 | 6288 | 0.92 | 1.09 | 1.01 | **0.81** | 0.91 | 0.92 | **0.81** | 0.95 |  |
| N-acetylthreonine | LC/MS neg | 33939 |  | 152204 | 0.96 | 1.01 | 0.95 | 0.94 | 0.99 | 0.95 | 1.01 | 1.04 | 0.99 |
| homoserine lactone | LC/MS pos early | 15696 |  | 445963 | 1.05 | 0.76 | 0.93 | 1.21 | 0.82 | 1.14 | 1.04 | 0.94 | 1.29 |
| alanine | LC/MS pos early | 1126 |  |  | 1.10 | 0.86 | 1.15 | **1.29** | 0.82 | 1.16 | 1.02 | 0.84 | 1.08 |
| N-acetylalanine | LC/MS neg | 1585 |  |  | 0.95 | 0.99 | 1.00 | 0.97 | 1.04 | 1.06 | 1.01 | 1.03 | 1.07 |
| aspartate | LC/MS pos early | 443 | HMDB00161 | 5960 | **0.83** | 0.94 | 1.06 | 1.00 | 0.96 | 0.92 | 1.10 | 0.91 | **0.82** |
| N-acetylaspartate (NAA) | LC/MS polar | 22185 | HMDB00766 | 65065 | 0.97 | 0.55 | 0.47 | **0.23** | 0.37 | 0.30 | 0.48 | 0.38 | 0.42 |
| asparagine | LC/MS pos early | 512 | HMDB00191 | 6267 | 1.00 | 1.01 | 1.00 | 0.94 | 0.96 | 0.90 | 0.91 | 1.02 | 0.94 |
| N-acetylasparagine | LC/MS pos early | 33942 | HMDB00812 | 99715 | 0.99 | 1.04 | 0.96 | 1.03 | 1.03 | 0.93 | 1.00 | 1.08 | 0.94 |
| glutamate | LC/MS pos early | 57 | HMDB00168 | 611 | 0.99 | 1.01 | 0.97 | 0.95 | 1.01 | 1.00 | 1.03 | 1.05 | 0.96 |
| N-acetylglutamate | LC/MS pos early | 15720 | HMDB01138 | 70914 | 0.95 | 1.01 | 0.92 | 0.96 | 0.98 | 1.00 | 0.97 | 0.98 | 0.99 |
| N-acetylglutamine | LC/MS pos early | 33943 | HMDB06029 | 182230 | 0.95 | 1.01 | 1.01 | 0.97 | 1.02 | 0.98 | 0.98 | 1.04 | 1.00 |
| glutamate, gamma-methyl ester | LC/MS pos early | 33487 | HMDB61715 | 68662 | 0.97 | 1.04 | 0.89 | 1.08 | 1.26 | 0.97 | 1.09 | 1.23 | 1.01 |
| pyroglutamine* | LC/MS pos early | 46225 |  | 134508 | 0.95 | 1.02 | 1.04 | 0.91 | 1.03 | 1.02 | 1.00 | 1.06 | 0.98 |
| N-acetyl-aspartyl-glutamate (NAAG) | LC/MS pos early | 35665 | HMDB01067 | 5255 | 0.99 | 1.11 | 1.00 | 0.99 | 1.21 | 1.09 | 0.93 | 1.16 | 1.02 |
| beta-citrylglutamate | LC/MS neg | 54923 |  | 72715786 | 1.07 | 1.00 | 1.08 | 1.01 | 0.93 | 1.05 | 1.08 | 0.92 | 0.98 |
| S-1-pyrroline-5-carboxylate | LC/MS pos early | 42370 | HMDB01301 | 1196 | 0.64 | 0.70 | 2.31 | 0.46 | 0.73 | **2.40** | 0.98 | 0.96 | 1.42 |
| histidine | LC/MS neg | 59 | HMDB00177 | 6274 | 1.02 | 1.03 | **0.80** | 1.05 | 0.96 | 0.86 | 0.92 | 0.92 | 0.99 |
| N-acetylhistidine | LC/MS pos early | 33946 | HMDB32055 | 75619 | 0.92 | 1.07 | 0.88 | 0.94 | 1.11 | 0.96 | 1.05 | 1.09 | 0.90 |
| imidazole propionate | LC/MS pos early | 40730 | HMDB02271 | 70630 | **0.87** | 1.02 | 0.97 | **0.83** | 1.00 | 1.10 | 0.94 | **1.18** | **1.22** |
| carnosine | LC/MS pos early | 1768 | HMDB00033 | 439224 | 1.03 | 1.08 | 0.97 | 1.02 | 1.01 | 0.99 | 1.08 | 1.03 | 0.94 |
| 1-ribosyl-imidazoleacetate* | LC/MS pos early | 61868 | HMDB02331 | 5117448 | 1.07 | 1.12 | 1.02 | 0.98 | **1.42** | 1.08 | 0.93 | **1.29** | 1.04 |
| 4-imidazoleacetate | LC/MS pos early | 32349 | HMDB02024 | 96215 | 0.98 | 1.06 | 1.03 | 1.13 | **1.35** | **1.43** | **1.19** | 1.04 | **1.52** |
| histidine methyl ester | LC/MS pos early | 32493 |  | 92893 | 0.78 | 1.30 | 0.55 | 1.19 | 1.22 | 0.83 | **1.90** | 1.41 | 1.04 |
| lysine | LC/MS pos early | 1301 | HMDB00182 | 5962 | 1.01 | 1.01 | 0.90 | 0.96 | 0.98 | 0.88 | 0.95 | 0.99 | 0.90 |
| N6-acetyllysine | LC/MS pos early | 36752 | HMDB00206 | 92832 | 0.89 | 1.04 | **0.77** | 0.92 | 0.97 | 0.86 | 0.98 | 0.98 | **0.80** |
| N6,N6,N6-trimethyllysine | LC/MS pos early | 1498 | HMDB01325 | 440120 | 0.97 | 0.99 | 1.05 | 0.96 | **0.81** | **0.88** | 0.97 | **0.80** | **0.86** |
| 5-(galactosylhydroxy)-L-lysine | LC/MS pos early | 43582 |  |  | 1.03 | 0.92 | 0.91 | 0.95 | 0.98 | 0.91 | 1.07 | 0.92 | 0.93 |
| saccharopine | LC/MS pos early | 1495 | HMDB00279 | 160556 | 0.90 | 1.19 | 0.85 | 0.90 | **1.26** | 1.00 | 1.20 | **1.45** | 0.98 |
| 2-aminoadipate | LC/MS pos early | 6146 | HMDB00510 | 469 | 1.03 | 1.30 | **0.59** | 1.11 | 1.24 | 0.89 | **1.78** | **1.53** | 0.84 |
| pipecolate | LC/MS pos early | 1444 | HMDB00070 | 849 | 1.00 | 1.41 | 1.34 | 1.01 | 1.23 | **1.51** | **1.53** | 1.25 | 1.36 |
| 6-oxopiperidine-2-carboxylate | LC/MS pos early | 43231 | HMDB61705 | 3014237 | **0.32** | 0.49 | 1.32 | 0.51 | 2.44 | 0.75 | 0.51 | 1.32 | 1.40 |
| cadaverine | LC/MS pos early | 15308 | HMDB02322 | 273 | 0.95 | 0.97 | 0.94 | 0.97 | 1.05 | **1.35** | 0.96 | 0.98 | **1.26** |
| N-acetyl-cadaverine | LC/MS pos early | 43530 | HMDB02284 | 189087 | **0.86** | 0.99 | 1.01 | 0.95 | 1.04 | **1.38** | 0.95 | 0.91 | 1.14 |
| 5-aminovalerate | LC/MS pos early | 18319 | HMDB03355 | 138 | 0.89 | 1.01 | 0.87 | 0.88 | 1.00 | **0.84** | 0.98 | 1.00 | **0.81** |
| N-trimethyl 5-aminovalerate | LC/MS pos early | 57687 |  |  | 0.90 | 1.03 | 0.98 | 0.92 | 0.96 | 1.00 | 0.98 | 1.01 | 0.91 |
| phenylalanine | LC/MS pos early | 64 | HMDB00159 | 6140 | 1.05 | 1.05 | 0.91 | 1.01 | 1.01 | 0.92 | 1.00 | 1.02 | 0.97 |
| N-acetylphenylalanine | LC/MS neg | 33950 | HMDB00512 | 74839 | **1.17** | 1.07 | 1.07 | 1.14 | 0.88 | 1.14 | 1.09 | 0.94 | 0.92 |
| phenyllactate (PLA) | LC/MS neg | 22130 | HMDB00779 | 3848 | 1.02 | 0.83 | 1.02 | 0.89 | 1.01 | **1.34** | 0.92 | 0.93 | 1.22 |
| tyrosine | LC/MS pos early | 1299 | HMDB00158 | 6057 | 1.01 | 1.07 | 0.97 | 0.99 | 1.12 | 0.94 | 0.93 | 1.10 | 0.94 |
| 1-carboxyethyltyrosine | LC/MS neg | 62564 |  |  | 1.09 | **1.44** | 0.91 | 0.99 | 1.14 | 0.98 | 1.04 | **1.49** | 1.11 |
| 3-(4-hydroxyphenyl)lactate | LC/MS neg | 32197 | HMDB00755 | 9378 | 0.96 | 0.99 | 1.03 | **0.86** | 0.95 | **1.29** | **0.83** | 0.96 | **1.14** |
| phenol sulfate | LC/MS neg | 32553 | HMDB60015 | 74426 | 0.88 | 0.97 | 1.04 | **0.80** | 0.96 | 1.00 | 0.90 | 0.98 | 0.99 |
| O-methyltyrosine | LC/MS pos early | 37451 | HMDB14903 | 76957 | 1.13 | 0.90 | 0.89 | 0.87 | 0.88 | 0.89 | 0.89 | 0.91 | 0.92 |
| tryptophan | LC/MS pos early | 54 | HMDB00929 | 6305 | 1.06 | 1.04 | 0.92 | 1.02 | 0.98 | 0.97 | 1.02 | 1.03 | 0.99 |
| C-glycosyltryptophan | LC/MS pos early | 48782 |  | 10981970 | 1.01 | 1.05 | 0.96 | 0.99 | 1.04 | 1.07 | 1.09 | 1.09 | 1.02 |
| kynurenine | LC/MS pos early | 15140 | HMDB00684 | 161166 | 1.77 | 0.73 | 0.83 | 0.76 | 1.20 | 0.93 | 1.38 | 0.69 | 1.22 |
| indolelactate | LC/MS neg | 18349 | HMDB00671 | 92904 | 0.89 | 0.95 | 1.05 | **0.80** | 1.00 | 1.12 | **0.84** | 1.13 | **1.35** |
| leucine | LC/MS pos early | 60 |  | 5246661 | 1.10 | 1.05 | 0.89 | 1.09 | 1.04 | 0.89 | 1.07 | 1.06 | 0.93 |
| N-acetylleucine | LC/MS neg | 1587 | HMDB11756 | 70912 | 0.86 | 0.92 | 1.03 | 1.05 | 0.89 | 1.09 | 0.98 | 1.04 | 0.97 |
| 1-carboxyethylleucine | LC/MS neg | 62559 |  |  | 1.29 | **1.46** | 0.77 | 1.16 | 1.27 | 0.79 | 1.20 | **1.97** | 0.97 |
| alpha-hydroxyisocaproate | LC/MS neg | 22132 | HMDB00746 | 83697 | 1.31 | 1.12 | 1.22 | 0.97 | 1.08 | **1.49** | 1.06 | 1.41 | **1.86** |
| isovalerylcarnitine (C5) | LC/MS pos early | 34407 | HMDB00688 | 6426851 | 0.91 | 1.01 | 0.80 | 0.86 | 0.80 | 1.11 | 0.79 | 1.01 | 1.08 |
| beta-hydroxyisovalerate | LC/MS polar | 12129 | HMDB00754 | 69362 | **0.79** | 0.98 | 1.03 | **0.68** | 0.84 | **1.29** | **0.70** | 0.91 | **1.82** |
| 3-methylglutaconate | LC/MS polar | 57747 | HMDB00522 | 1551553 | 0.99 | 0.99 | 1.08 | 1.00 | 0.99 | **1.23** | 1.03 | 0.97 | 1.17 |
| isoleucine | LC/MS pos early | 1125 | HMDB00172 | 6306 | 1.06 | 1.09 | 0.91 | 1.03 | 1.05 | 0.91 | 1.00 | 1.06 | 0.94 |
| N-acetylisoleucine | LC/MS neg | 33967 | HMDB61684 | 2802421 | 0.86 | 0.82 | 0.90 | 1.45 | 0.80 | 0.87 | 1.08 | 0.93 | 1.05 |
| 1-carboxyethylisoleucine | LC/MS neg | 62558 |  |  | 1.08 | 1.07 | 0.79 | 0.95 | **0.74** | 0.90 | 1.03 | 1.07 | 0.96 |
| alpha-hydroxyisovalerate | LC/MS polar | 46537 | HMDB00407 | 99823 | 1.08 | **1.46** | 0.80 | 1.04 | 1.14 | 1.15 | 0.96 | 1.26 | **1.49** |
| 2-methylbutyrylcarnitine (C5) | LC/MS pos early | 45095 | HMDB00378 | 6426901 | 0.92 | 1.09 | **0.85** | 0.89 | 0.96 | **0.85** | 1.00 | 1.02 | **0.80** |
| 3-hydroxy-2-ethylpropionate | LC/MS polar | 32397 | HMDB00396 | 188979 | 1.27 | 1.17 | 1.34 | 1.32 | 1.26 | 1.19 | 1.08 | 1.16 | 0.87 |
| ethylmalonate | LC/MS polar | 15765 | HMDB00622 | 11756 | 0.99 | 1.05 | 0.97 | 1.04 | **1.19** | **1.31** | 1.06 | **1.17** | **1.22** |
| methylsuccinate | LC/MS polar | 15745 | HMDB01844 | 10349 | 0.89 | 0.82 | **2.02** | 0.64 | 0.90 | 1.18 | 0.85 | 0.97 | 1.00 |
| valine | LC/MS pos early | 1649 | HMDB00883 | 6287 | 1.09 | 1.08 | 0.90 | 1.05 | 1.02 | 0.88 | 1.01 | 1.08 | 0.92 |
| N-acetylvaline | LC/MS neg | 1591 | HMDB11757 | 66789 | 1.00 | 0.93 | **1.36** | **0.77** | 0.91 | 1.13 | 1.05 | 0.93 | **1.18** |
| 1-carboxyethylvaline | LC/MS neg | 62562 |  |  | 1.06 | **1.26** | 0.82 | 1.06 | 0.98 | 0.84 | 1.07 | 1.16 | 0.99 |
| isobutyrylcarnitine (C4) | LC/MS pos early | 33441 | HMDB00736 | 168379 | 0.95 | 0.96 | **1.54** | 1.11 | 1.12 | **1.50** | 1.16 | 1.28 | 1.16 |
| 3-hydroxyisobutyrate | LC/MS polar | 1549 | HMDB00336 | 87 | **0.71** | 1.00 | 1.02 | **0.60** | 0.85 | 1.13 | **0.76** | 0.95 | **1.22** |
| methionine | LC/MS pos early | 1302 | HMDB00696 | 6137 | 1.11 | 1.07 | 0.89 | 1.04 | 1.04 | 0.86 | 1.04 | 1.06 | 0.88 |
| N-acetylmethionine | LC/MS neg | 1589 | HMDB11745 | 448580 | **0.86** | 0.97 | 1.05 | **0.90** | 0.98 | 1.06 | **0.87** | 0.93 | 1.06 |
| N-formylmethionine | LC/MS neg | 2829 | HMDB01015 | 439750 | 0.96 | 1.01 | 1.02 | **0.88** | 0.96 | 1.06 | **0.87** | 0.99 | 1.06 |
| methionine sulfoxide | LC/MS pos early | 18374 | HMDB02005 | 158980 | 1.00 | 1.09 | 0.93 | 0.92 | 1.00 | 0.98 | 0.94 | 1.07 | 0.96 |
| N-acetylmethionine sulfoxide | LC/MS pos early | 45428 |  | 193368 | 0.81 | 1.11 | 0.91 | 0.97 | 1.20 | 1.06 | 1.01 | 1.18 | 1.01 |
| S-adenosylmethionine (SAM) | LC/MS pos early | 15915 | HMDB01185 | 34756 | 1.03 | 1.01 | 0.94 | 1.06 | 1.03 | 1.08 | 1.12 | **1.16** | **1.20** |
| S-adenosylhomocysteine (SAH) | LC/MS neg | 42382 | HMDB00939 | 439155 | 1.03 | 0.97 | 0.92 | 0.96 | 1.01 | 1.00 | 1.03 | 1.09 | 1.01 |
| homocysteine | LC/MS pos early | 15128 | HMDB00742 | 778 | **0.78** | 0.93 | 1.08 | **0.80** | 0.95 | **1.34** | **0.75** | 1.08 | **1.41** |
| cystathionine | LC/MS pos early | 15705 | HMDB00099 | 439258 | 0.89 | 0.96 | **0.82** | 0.87 | 1.06 | 1.13 | 1.06 | **1.25** | 1.14 |
| cysteine | LC/MS pos early | 1868 | HMDB00574 | 5862 | 0.98 | 1.05 | 0.96 | 0.99 | **1.17** | 0.96 | 1.12 | **1.24** | 0.96 |
| N-acetylcysteine | LC/MS pos early | 1586 | HMDB01890 | 12035 | 0.94 | 1.02 | 0.94 | 1.20 | 1.17 | **1.41** | 1.00 | 1.07 | **1.46** |
| lanthionine | LC/MS pos early | 42002 |  | 98504 | 0.96 | 1.07 | 0.77 | **1.39** | 0.98 | 0.93 | 0.83 | 0.79 | 0.82 |
| cysteine sulfinic acid | LC/MS neg | 37443 | HMDB00996 | 109 | 0.88 | 1.31 | 0.64 | **0.52** | 1.26 | 0.70 | 0.90 | 1.45 | 0.62 |
| hypotaurine | LC/MS pos early | 590 | HMDB00965 | 107812 | 0.99 | 0.98 | 1.06 | 0.93 | 0.95 | 1.05 | 1.04 | 1.01 | 0.97 |
| taurine | LC/MS pos early | 2125 | HMDB00251 | 1123 | 0.96 | 1.00 | 0.99 | **0.89** | 0.94 | 1.02 | 0.99 | 0.99 | 0.94 |
| N-acetyltaurine | LC/MS neg | 48187 |  | 159864 | 0.96 | 1.03 | 1.00 | **0.90** | 1.09 | **1.15** | 1.05 | **1.22** | **1.24** |
| 3-sulfo-L-alanine | LC/MS polar | 47089 | HMDB02757 | 72886 | 0.81 | **3.51** | **0.11** | 1.95 | 2.41 | 0.48 | 2.12 | 2.93 | 0.70 |
| arginine | LC/MS pos early | 1638 | HMDB00517 | 232 | 0.98 | 0.96 | 0.94 | 0.96 | 0.97 | 0.92 | 0.92 | 0.98 | 0.91 |
| argininosuccinate | LC/MS pos early | 15497 | HMDB00052 | 828 | 0.96 | 1.03 | 1.19 | 1.00 | 1.32 | 1.08 | 1.10 | 1.46 | 1.22 |
| ornithine | LC/MS pos early | 1493 | HMDB03374 | 6262 | **0.79** | 1.17 | 1.04 | **0.72** | **0.56** | **0.44** | **0.66** | **0.49** | **0.39** |
| 2-oxoarginine* | LC/MS pos early | 55072 | HMDB04225 | 558 | 0.73 | 0.85 | 1.00 | 1.05 | 0.77 | 1.10 | 0.79 | **0.59** | 1.10 |
| proline | LC/MS pos early | 1898 | HMDB00162 | 145742 | 0.95 | 1.00 | 1.01 | **0.90** | 0.94 | 1.05 | 0.93 | 1.00 | 1.00 |
| dimethylarginine (SDMA + ADMA) | LC/MS pos early | 36808 | HMDB01539 | 123831 | **0.82** | 0.89 | 1.06 | 0.86 | 0.94 | 0.96 | 0.89 | 1.04 | 0.94 |
| N-acetylarginine | LC/MS pos early | 33953 | HMDB04620 | 67427 | **0.80** | 1.13 | **0.78** | **0.80** | 0.96 | **0.79** | 0.88 | 1.05 | **0.77** |
| trans-4-hydroxyproline | LC/MS pos early | 32306 | HMDB00725 | 5810 | 0.91 | 1.03 | 0.99 | **0.89** | **0.86** | **0.87** | **0.86** | 0.92 | **0.83** |
| pro-hydroxy-pro | LC/MS pos early | 35127 | HMDB06695 | 11673055 | **3.69** | 1.21 | 0.45 | 1.35 | 0.51 | 0.78 | **4.10** | 0.75 | 0.43 |
| N-monomethylarginine | LC/MS pos early | 43586 | HMDB29416 | 132862 | 0.88 | 0.89 | 1.10 | 0.93 | 0.90 | 0.96 | 0.87 | 0.98 | 0.96 |
| guanidinoacetate | LC/MS pos early | 43802 | HMDB00128 | 763 | 1.03 | 1.04 | 1.15 | 0.98 | **1.34** | 1.11 | 1.10 | **1.38** | 1.11 |
| creatine | LC/MS pos early | 27718 | HMDB00064 | 586 | 0.93 | 1.00 | 0.99 | **0.91** | 1.00 | 1.03 | 0.95 | 1.02 | 0.96 |
| creatinine | LC/MS pos early | 513 | HMDB00562 | 588 | 1.00 | 1.12 | 0.96 | 1.02 | 1.04 | 1.13 | 1.02 | 1.06 | 0.97 |
| creatine phosphate | LC/MS polar | 33951 | HMDB01511 | 587 | 0.64 | 0.66 | 0.80 | 0.72 | 0.57 | 0.79 | 0.64 | **0.50** | 0.91 |
| putrescine | LC/MS pos early | 1408 | HMDB01414 | 1045 | **0.78** | 0.99 | 0.91 | **0.75** | 1.04 | 1.10 | **0.68** | **0.80** | 0.94 |
| spermidine | LC/MS pos early | 485 | HMDB01257 | 1102 | 1.03 | 0.94 | **0.84** | 1.02 | **0.80** | 0.92 | 0.90 | **0.76** | **0.85** |
| N('1)-acetylspermidine | LC/MS pos early | 57689 | HMDB01276 | 496 | 0.95 | 1.06 | **0.85** | 0.97 | 0.97 | 1.00 | 1.01 | 1.03 | 0.98 |
| spermine | LC/MS pos late | 603 | HMDB01256 | 1103 | 1.48 | 0.80 | 0.44 | 1.40 | 0.52 | 1.23 | 0.61 | 0.28 | 1.53 |
| N(1)-acetylspermine | LC/MS pos early | 32360 | HMDB01186 | 916 | 1.03 | 0.94 | 0.93 | 0.95 | 0.72 | 1.04 | 0.82 | 0.75 | 0.99 |
| N1,N12-diacetylspermine | LC/MS pos early | 52987 | HMDB02172 | 132680 | 1.11 | 1.28 | 0.85 | 1.22 | 1.04 | 1.02 | 1.24 | 1.17 | 0.93 |
| 5-methylthioadenosine (MTA) | LC/MS pos early | 1419 | HMDB01173 | 439176 | 0.98 | 0.97 | **0.91** | **0.91** | 1.04 | 1.07 | 0.95 | 1.01 | 0.98 |
| N-acetylputrescine | LC/MS pos early | 37496 | HMDB02064 | 122356 | **0.87** | 1.01 | 0.92 | **0.87** | **1.17** | **1.15** | 0.92 | 0.98 | 0.94 |
| 4-guanidinobutanoate | LC/MS pos early | 15681 | HMDB03464 | 500 | 1.20 | **1.49** | **1.36** | 1.00 | 1.12 | 1.11 | 1.17 | 1.13 | 0.97 |
| glutathione, reduced (GSH) | LC/MS pos early | 2127 | HMDB00125 | 124886 | 1.09 | 1.08 | 1.33 | 0.96 | **1.48** | 1.12 | 1.10 | **1.45** | 1.05 |
| glutathione, oxidized (GSSG) | LC/MS pos early | 27727 | HMDB03337 | 65359 | 1.02 | 1.19 | 0.89 | 0.98 | 1.18 | 0.90 | 1.08 | 1.13 | 0.89 |
| cysteine-glutathione disulfide | LC/MS pos early | 35159 | HMDB00656 | 4247235 | 0.97 | 1.02 | 0.92 | 1.03 | 1.12 | 1.00 | 1.17 | **1.32** | 1.08 |
| S-methylglutathione | LC/MS pos early | 33944 |  | 3605667 | 1.28 | 1.12 | 0.93 | 1.00 | **1.94** | 1.09 | 0.91 | 1.49 | 1.10 |
| S-lactoylglutathione | LC/MS neg | 15731 | HMDB01066 | 440018 | 1.36 | 0.86 | 0.87 | 1.25 | **0.52** | 0.62 | 0.78 | **0.26** | 0.62 |
| cysteinylglycine | LC/MS pos early | 35637 | HMDB00078 | 439498 | 1.01 | 1.09 | 0.93 | 1.08 | 0.97 | 0.95 | **1.24** | 0.97 | 0.86 |
| 5-oxoproline | LC/MS neg | 1494 | HMDB00267 | 7405 | 0.97 | 0.90 | 1.07 | 0.85 | 1.06 | 1.24 | 1.02 | 1.20 | 1.11 |
| S-nitrosoglutathione (GSNO) | LC/MS pos early | 47127 | HMDB04645 | 3514 | 0.93 | 0.77 | **0.42** | 0.65 | 0.87 | **0.42** | 0.78 | **1.57** | **0.53** |
| 4-hydroxy-nonenal-glutathione | LC/MS neg | 48504 |  |  | 0.80 | 1.18 | 0.76 | 0.79 | **1.43** | 0.80 | 0.88 | 0.95 | 0.77 |
| gamma-glutamylcysteine | LC/MS pos early | 1778 | HMDB01049 | 842 | 1.00 | 0.86 | 1.00 | 1.19 | 0.86 | 1.07 | **1.42** | 0.95 | 0.83 |
| gamma-glutamylglutamate | LC/MS pos early | 36738 | HMDB11737 | 92865 | 1.05 | **0.78** | 1.06 | 1.06 | **0.81** | 1.06 | 1.08 | 0.88 | 1.00 |
| gamma-glutamylglutamine | LC/MS pos early | 2730 | HMDB11738 | 150914 | 0.94 | 0.99 | 1.03 | 0.90 | **0.72** | 0.89 | 0.89 | **0.75** | **0.83** |
| gamma-glutamylglycine | LC/MS pos early | 33949 | HMDB11667 | 165527 | 1.00 | 1.00 | 1.35 | 1.00 | 1.00 | 1.26 | 1.18 | 1.26 | 1.17 |
| gamma-glutamylisoleucine* | LC/MS pos early | 34456 | HMDB11170 | 14253342 | 0.96 | 1.12 | **0.83** | 0.95 | 1.10 | 0.90 | 1.04 | 1.04 | 0.90 |
| gamma-glutamylleucine | LC/MS neg | 18369 | HMDB11171 | 151023 | 1.02 | 1.01 | 0.97 | 1.03 | 0.93 | **0.82** | 1.04 | 0.94 | 0.94 |
| gamma-glutamylmethionine | LC/MS pos early | 44872 | HMDB29155 | 7009567 | 1.07 | 1.01 | 0.87 | 0.97 | 0.89 | 0.87 | 1.06 | 0.92 | 0.91 |
| gamma-glutamylthreonine | LC/MS pos early | 33364 | HMDB29159 | 76078708 | 0.95 | 0.97 | 1.13 | 1.17 | **0.72** | 1.14 | 1.13 | **0.64** | 0.88 |
| gamma-glutamylvaline | LC/MS pos early | 43829 | HMDB11172 | 7015683 | 1.17 | 1.02 | 1.06 | 1.19 | 1.03 | 1.12 | 0.97 | 1.27 | 0.94 |
| alanylleucine | LC/MS pos early | 37093 | HMDB28691 | 259583 | 0.84 | 1.15 | **0.73** | 0.89 | 1.07 | 0.88 | 0.84 | 0.88 | 0.84 |
| glutaminylleucine | LC/MS pos early | 42994 |  |  | 0.66 | 1.22 | 0.56 | 0.99 | 0.79 | 0.79 | 0.79 | 0.69 | 0.69 |
| glycylleucine | LC/MS pos early | 34398 | HMDB00759 | 92843 | 0.87 | 1.14 | 0.80 | 0.93 | 1.21 | 0.86 | 0.84 | 1.02 | 0.89 |
| glycylvaline | LC/MS pos early | 18357 | HMDB28854 | 97417 | 0.98 | 1.05 | 0.89 | 0.93 | 1.26 | 0.99 | 0.94 | 1.21 | 0.94 |
| isoleucylglycine | LC/MS pos early | 40008 | HMDB28907 | 342532 | 1.14 | 1.05 | 0.85 | 1.16 | 1.01 | 0.90 | 1.18 | 0.89 | 0.92 |
| leucylalanine | LC/MS pos early | 40010 | HMDB28922 | 259321 | 0.78 | 1.07 | 1.02 | 0.88 | 0.85 | 1.18 | 0.83 | 0.70 | 1.12 |
| leucylglycine | LC/MS pos early | 40045 | HMDB28929 | 79070 | 1.01 | 1.09 | 1.05 | 1.16 | **0.60** | 1.28 | 1.16 | **0.45** | 1.12 |
| lysylleucine | LC/MS pos early | 40020 | HMDB28955 | 4682588 | 1.39 | 1.23 | 0.78 | 1.47 | 1.03 | 0.76 | 1.41 | 1.14 | 0.85 |
| phenylalanylalanine | LC/MS pos early | 41374 |  | 5488196 | **1.22** | **1.26** | 1.01 | **1.30** | 1.17 | 1.14 | **1.37** | 1.08 | 1.16 |
| phenylalanylglycine | LC/MS pos early | 41370 | HMDB28995 | 98207 | 1.25 | 1.19 | 0.91 | 1.23 | 1.16 | 1.02 | 1.27 | 1.13 | 1.06 |
| prolylglycine | LC/MS pos early | 40703 | HMDB11178 | 6426709 | 1.25 | **1.64** | 1.06 | 1.36 | **1.85** | 1.36 | 1.47 | **2.60** | 1.14 |
| threonylphenylalanine | LC/MS neg | 31530 | HMDB29068 | 4099799 | 1.11 | 1.09 | 1.13 | 1.09 | 0.94 | 1.12 | 1.06 | 0.87 | **1.17** |
| tryptophylglycine | LC/MS pos early | 43028 | HMDB29083 | 263471 | 1.35 | 1.34 | 1.06 | 1.41 | **1.61** | 1.30 | **1.59** | 1.29 | **1.57** |
| tyrosylglycine | LC/MS neg | 41375 | HMDB29105 | 259323 | 1.15 | 1.14 | 1.04 | 1.13 | 1.14 | 1.11 | 1.22 | 1.12 | 1.17 |
| valylglutamine | LC/MS pos early | 42079 | HMDB29125 | 5253209 | 0.85 | 1.16 | 0.80 | 0.95 | 0.77 | 1.00 | 1.03 | **0.62** | 0.95 |
| valylglycine | LC/MS pos early | 40475 | HMDB29127 | 136487 | 1.33 | 0.85 | 1.01 | 1.32 | 0.77 | 1.10 | 1.28 | **0.55** | 1.06 |
| valylleucine | LC/MS pos early | 39994 | HMDB29131 | 352039 | 0.86 | 1.21 | 0.81 | 1.22 | 1.01 | 1.12 | 1.17 | 0.86 | 1.11 |
| leucylglutamine* | LC/MS pos early | 48189 | HMDB28927 | 4305457 | 0.74 | 1.04 | 0.99 | 0.84 | 0.80 | 1.16 | 0.85 | 0.71 | 1.05 |
| glucose | LC/MS polar | 20488 | HMDB00122 | 79025 | 1.38 | 1.07 | 0.64 | 1.33 | 1.19 | 0.84 | **1.90** | 1.16 | 0.82 |
| glucose 6-phosphate | LC/MS polar | 31260 | HMDB01401 | 5958 | 0.79 | 1.26 | **1.86** | 0.90 | 1.44 | **2.01** | 1.04 | 1.61 | **1.81** |
| fructose-6-phosphate | LC/MS polar | 12021 | HMDB00124 | 69507 | 1.50 | 1.30 | 0.72 | **1.58** | 1.39 | 0.84 | **1.71** | **1.65** | 0.84 |
| fructose 1,6-diphosphate/glucose 1,6-diphosphate/myo-inositol diphosphates | LC/MS neg | 46896 |  |  | **1.95** | 1.15 | **0.56** | 1.38 | 0.87 | **0.53** | 0.73 | 0.73 | **0.54** |
| dihydroxyacetone phosphate (DHAP) | LC/MS pos early | 15522 | HMDB01473 | 668 | 1.40 | 0.93 | 0.78 | 1.00 | 0.86 | **0.58** | **0.58** | 0.71 | **0.60** |
| 2-phosphoglycerate | LC/MS polar | 35629 | HMDB03391 | 59 | 1.16 | 0.90 | **0.67** | 1.05 | 0.76 | **0.70** | **0.57** | **0.61** | **0.64** |
| 3-phosphoglycerate | LC/MS neg | 1414 | HMDB00807 | 724 | 1.15 | 0.88 | 0.78 | 1.10 | **0.71** | 0.76 | **0.56** | **0.53** | **0.62** |
| phosphoenolpyruvate (PEP) | LC/MS neg | 597 | HMDB00263 | 1005 | 1.17 | 0.89 | 0.73 | 1.04 | 0.82 | **0.59** | **0.65** | **0.69** | **0.65** |
| pyruvate | LC/MS polar | 22250 | HMDB00243 | 1060 | 1.02 | 1.14 | **0.77** | 1.13 | **1.24** | 1.10 | **1.27** | **1.21** | 1.17 |
| lactate | LC/MS neg | 527 | HMDB00190 | 612 | 1.03 | 0.89 | 1.07 | 0.94 | 0.93 | 1.09 | 1.00 | 0.95 | 1.04 |
| glycerate | LC/MS polar | 1572 | HMDB00139 | 752 | 0.88 | 1.01 | 1.03 | **0.87** | 1.06 | **1.15** | 0.88 | 1.00 | **1.21** |
| 6-phosphogluconate | LC/MS neg | 15442 | HMDB01316 | 91493 | 1.14 | 1.40 | 1.05 | 1.21 | 1.30 | 1.15 | 0.91 | 1.20 | 1.22 |
| ribulose 5-phosphate | LC/MS polar | 1474 | HMDB00618 | 439184 | 1.14 | 0.89 | 0.99 | 0.95 | 0.88 | 0.90 | 0.83 | 0.85 | 0.79 |
| ribose 1-phosphate | LC/MS polar | 1763 | HMDB01489 | 439236 | 0.86 | 0.94 | 0.91 | **0.80** | 0.95 | 0.90 | 0.86 | 0.82 | **0.81** |
| 5-phosphoribosyl diphosphate (PRPP) | LC/MS neg | 36840 | HMDB00280 | 7339 | 1.04 | 1.02 | 0.89 | 0.94 | 1.13 | 1.13 | 0.89 | **1.30** | **1.43** |
| sedoheptulose-7-phosphate | LC/MS neg | 35649 | HMDB01068 | 616 | 0.82 | 1.20 | 1.01 | 0.83 | 1.18 | 1.12 | 1.06 | **1.35** | 1.05 |
| ribose | LC/MS polar | 1471 | HMDB00283 | 5779 | 0.63 | 0.83 | 0.95 | **0.37** | 0.63 | 1.09 | 0.94 | 0.78 | 1.20 |
| ribitol | LC/MS polar | 15772 | HMDB00508 | 6912 | 0.98 | 0.98 | 1.07 | 0.93 | 0.99 | 1.09 | 1.07 | 1.02 | 1.07 |
| ribonate | LC/MS polar | 27731 | HMDB00867 | 5460677 | 0.98 | 1.05 | 1.00 | 0.93 | 0.99 | 0.98 | 1.08 | 1.08 | 1.02 |
| xylulose 5-phosphate | LC/MS polar | 37285 | HMDB00868 | 439190 | 0.98 | 1.21 | 0.85 | 0.72 | 0.89 | 0.86 | 0.84 | 1.32 | 0.47 |
| arabitol/xylitol | LC/MS polar | 48885 |  | 6912 | 1.00 | 1.01 | 1.03 | 0.93 | 0.93 | 0.95 | 1.05 | 0.99 | 0.94 |
| sedoheptulose | LC/MS polar | 53237 | HMDB03219 | 5459879 | 1.53 | 1.51 | 0.65 | 1.00 | **2.32** | 0.99 | 1.40 | **2.01** | 1.19 |
| fructose | LC/MS polar | 577 | HMDB00660 | 5984 | 0.89 | 1.21 | 0.92 | 1.00 | 1.36 | 1.10 | 1.14 | 1.32 | 1.02 |
| mannitol/sorbitol | LC/MS polar | 46142 | HMDB00247 | 5780 | 1.08 | 1.01 | 0.92 | **1.19** | 0.96 | 1.00 | **1.18** | 1.01 | 0.95 |
| mannose-6-phosphate | LC/MS polar | 1469 | HMDB01078 | 439198 | 0.86 | 1.15 | **1.45** | 0.87 | 1.16 | **1.42** | 0.99 | 1.30 | 1.24 |
| galactitol (dulcitol) | LC/MS polar | 1117 | HMDB00107 | 11850 | 1.00 | 1.11 | 1.00 | **0.74** | 0.94 | **0.81** | 0.97 | 0.95 | 0.86 |
| galactose 1-phosphate | LC/MS polar | 15706 | HMDB00645 | 123912 | **1.58** | 1.42 | 0.78 | **1.55** | 1.38 | 0.81 | **1.64** | **1.60** | 0.83 |
| galactonate | LC/MS polar | 27719 | HMDB00565 | 128869 | 1.46 | 1.31 | **0.40** | 1.59 | 1.63 | 0.67 | **1.76** | **1.75** | 1.15 |
| UDP-glucose | LC/MS polar | 32344 | HMDB00286 | 8629 | **1.24** | 1.12 | 0.97 | **1.18** | 1.04 | 0.96 | **1.35** | 1.08 | 0.96 |
| UDP-galactose | LC/MS polar | 15860 | HMDB00302 | 18068 | 0.78 | 0.91 | 1.26 | **0.69** | 0.85 | 1.12 | 0.81 | 0.88 | 1.04 |
| UDP-glucuronate | LC/MS neg | 2763 | HMDB00935 | 17473 | 0.98 | 0.98 | 0.97 | 0.92 | 0.91 | 0.91 | 0.94 | 0.92 | **0.86** |
| guanosine 5'-diphospho-fucose | LC/MS neg | 15903 |  |  | 0.92 | 0.96 | 0.96 | **0.81** | 0.98 | 0.95 | **0.82** | 0.95 | 0.93 |
| UDP-N-acetylglucosamine/galactosamine | LC/MS polar | 46148 |  |  | 1.01 | 1.04 | 1.17 | 0.90 | 0.92 | 0.97 | 1.17 | 1.07 | 0.90 |
| cytidine 5'-monophospho-N-acetylneuraminic acid | LC/MS polar | 36831 | HMDB01176 | 448209 | 1.01 | 0.60 | 0.73 | 0.87 | 0.62 | 0.71 | 1.02 | 0.61 | 0.98 |
| glucuronate 1-phosphate* | LC/MS neg | 52312 | HMDB03976 |  | 1.23 | 0.99 | 0.81 | 0.94 | 0.92 | **0.74** | 0.82 | 0.91 | 0.82 |
| glucosamine-6-phosphate | LC/MS polar | 580 | HMDB01254 | 439217 | 0.64 | **2.56** | 1.07 | 0.96 | **2.68** | 1.09 | 1.22 | **2.80** | 1.20 |
| glucuronate | LC/MS polar | 15443 | HMDB00127 | 444791 | 1.17 | **0.25** | 2.81 | 0.36 | 0.40 | 1.55 | 0.37 | 0.35 | 1.11 |
| N-acetylglucosamine 6-phosphate | LC/MS polar | 15107 | HMDB02817 | 439219 | 1.24 | 1.13 | 1.02 | 1.05 | 1.06 | 0.88 | 1.07 | 1.05 | 0.95 |
| N-acetyl-glucosamine 1-phosphate | LC/MS polar | 15741 | HMDB01367 | 440364 | 1.04 | 0.82 | 1.26 | 0.86 | 0.92 | 1.27 | 1.12 | 1.10 | **1.32** |
| N-acetylneuraminate | LC/MS polar | 32377 | HMDB00230 | 439197 | 1.02 | 1.02 | 0.90 | 1.01 | 1.01 | 0.94 | 1.10 | 1.04 | 0.91 |
| N-acetylglucosaminylasparagine | LC/MS pos early | 48149 | HMDB00489 | 123826 | 0.98 | 0.94 | 1.06 | 0.96 | **0.92** | 1.09 | 1.00 | 0.96 | 1.02 |
| erythronate* | LC/MS polar | 42420 | HMDB00613 | 2781043 | 1.02 | 0.97 | 1.00 | 0.97 | 1.01 | 1.08 | 1.06 | 1.02 | 1.06 |
| N-acetylglucosamine/N-acetylgalactosamine | LC/MS pos early | 46539 | HMDB00215 | 24139 | 0.91 | 1.01 | **0.86** | 0.92 | 0.93 | **0.88** | 0.95 | 1.00 | **0.85** |
| citrate | LC/MS neg | 1564 | HMDB00094 | 311 | 1.13 | 0.96 | 1.06 | 0.88 | 1.06 | **1.45** | 1.04 | 1.24 | **1.33** |
| aconitate [cis or trans] | LC/MS neg | 46173 |  |  | **1.36** | 0.95 | 1.00 | 0.89 | 1.07 | 1.28 | 1.05 | 1.25 | 1.19 |
| alpha-ketoglutarate | LC/MS polar | 528 | HMDB00208 | 51 | 0.99 | 1.15 | 0.99 | 1.10 | 1.26 | 1.14 | 1.20 | 1.23 | 1.23 |
| succinate | LC/MS polar | 1437 | HMDB00254 | 1110 | 0.85 | 0.49 | 1.09 | 0.23 | 0.37 | 0.68 | 0.53 | 0.46 | 0.59 |
| fumarate | LC/MS polar | 1643 | HMDB00134 | 444972 | 0.96 | 1.03 | **0.55** | 0.93 | 1.05 | 0.76 | 1.06 | 1.20 | 1.21 |
| malate | LC/MS polar | 1303 | HMDB00156 | 525 | 1.18 | 0.33 | **5.52** | **0.28** | 0.45 | 2.08 | 0.51 | 0.64 | 1.06 |
| 2-methylcitrate | LC/MS polar | 37483 | HMDB00379 | 439681 | 1.48 | 0.63 | **2.70** | 0.94 | 0.56 | 1.48 | 1.08 | 0.69 | 1.50 |
| phosphate | LC/MS neg | 42109 | HMDB01429 | 1061 | **0.89** | 0.93 | 0.92 | **0.82** | **0.90** | 0.97 | **0.81** | **0.87** | 0.92 |
| acetyl CoA | LC/MS neg | 43840 | HMDB01206 | 444493 | **0.50** | 1.13 | 1.04 | 0.91 | 1.18 | **1.62** | 0.93 | 1.25 | 1.32 |
| laurate (12:0) | LC/MS neg | 1645 | HMDB00638 | 3893 | **1.48** | 0.85 | 0.93 | 0.87 | 1.00 | **0.53** | 1.03 | 0.87 | 0.80 |
| 5-dodecenoate (12:1n7) | LC/MS neg | 33968 | HMDB00529 | 5312378 | 0.94 | 0.74 | 0.95 | 0.75 | 0.68 | **0.38** | 0.71 | 0.61 | **0.56** |
| myristate (14:0) | LC/MS neg | 1365 | HMDB00806 | 11005 | **1.96** | 1.08 | 1.07 | 1.17 | 1.42 | 0.68 | 1.54 | 1.29 | 1.04 |
| myristoleate (14:1n5) | LC/MS neg | 32418 | HMDB02000 | 5281119 | 1.75 | 0.85 | 0.82 | 0.94 | 0.84 | **0.40** | 0.99 | 0.67 | 0.64 |
| pentadecanoate (15:0) | LC/MS neg | 1361 | HMDB00826 | 13849 | 1.24 | 1.08 | 1.19 | 0.92 | 1.49 | 1.02 | 1.26 | 1.47 | 1.27 |
| palmitoleate (16:1n7) | LC/MS neg | 33447 | HMDB03229 | 445638 | **2.59** | 0.99 | 1.03 | 1.09 | 1.25 | 0.51 | 1.39 | 1.14 | 0.90 |
| 10-heptadecenoate (17:1n7) | LC/MS neg | 33971 | HMDB60038 | 5312435 | **2.75** | 1.08 | 0.98 | 1.20 | 1.42 | 0.53 | 1.54 | 1.33 | 1.01 |
| oleate/vaccenate (18:1) | LC/MS neg | 52285 |  |  | **2.65** | 1.15 | 0.99 | 1.28 | 1.37 | 0.50 | 1.62 | 1.21 | 0.91 |
| 10-nonadecenoate (19:1n9) | LC/MS neg | 33972 | HMDB13622 | 5312513 | **2.43** | 1.09 | 1.02 | 1.31 | 1.41 | 0.54 | 1.70 | 1.22 | 0.92 |
| eicosenoate (20:1) | LC/MS neg | 33587 | HMDB02231 | 5282768 | 1.85 | 1.06 | 1.10 | 1.18 | 1.40 | 0.63 | 1.66 | 1.28 | 1.02 |
| erucate (22:1n9) | LC/MS neg | 1552 | HMDB02068 | 5281116 | 1.48 | 0.88 | 0.99 | 0.76 | 1.28 | 0.83 | 1.39 | 1.35 | 1.01 |
| eicosapentaenoate (EPA; 20:5n3) | LC/MS neg | 18467 | HMDB01999 | 446284 | **3.13** | 1.06 | 1.00 | 1.57 | 1.42 | 0.62 | 1.89 | 1.44 | 0.76 |
| docosapentaenoate (n3 DPA; 22:5n3) | LC/MS neg | 32504 | HMDB06528 | 6441454 | **2.79** | 1.06 | 1.04 | 1.52 | 1.57 | 0.73 | 1.99 | 1.57 | 0.84 |
| docosahexaenoate (DHA; 22:6n3) | LC/MS neg | 44675 | HMDB02183 | 445580 | **3.20** | 1.24 | 1.07 | 1.87 | 1.59 | 0.70 | **2.22** | 1.57 | 0.93 |
| nisinate (24:6n3) | LC/MS neg | 57810 | HMDB02007 | 11792612 | **3.11** | 1.21 | 1.25 | **2.51** | 1.61 | 0.84 | **3.69** | 1.68 | 1.44 |
| linoleate (18:2n6) | LC/MS neg | 1105 | HMDB00673 | 5280450 | **2.67** | 1.09 | 1.61 | 1.14 | 1.36 | 0.93 | 1.44 | 1.24 | 0.92 |
| linolenate [alpha or gamma; (18:3n3 or 6)] | LC/MS neg | 34035 | HMDB03073 | 5280934 | **2.36** | 1.18 | 1.11 | 1.05 | 1.04 | 0.65 | 1.14 | 1.00 | 1.11 |
| dihomo-linolenate (20:3n3 or n6) | LC/MS neg | 35718 | HMDB02925 | 5280581 | **2.91** | 1.10 | 1.02 | 1.44 | 1.61 | 0.69 | 1.73 | 1.35 | 0.89 |
| arachidonate (20:4n6) | LC/MS neg | 1110 | HMDB01043 | 444899 | **2.62** | 1.20 | 0.95 | 1.50 | 1.50 | 0.72 | 1.85 | 1.42 | 0.90 |
| docosadienoate (22:2n6) | LC/MS neg | 32415 | HMDB61714 | 5282807 | **2.03** | 1.07 | 1.15 | 1.26 | 1.37 | 0.73 | 1.92 | 1.39 | 1.13 |
| dihomo-linoleate (20:2n6) | LC/MS neg | 17805 | HMDB05060 | 6439848 | **2.54** | 1.18 | 0.82 | 1.45 | 1.57 | **0.45** | 1.91 | 1.37 | 0.67 |
| mead acid (20:3n9) | LC/MS neg | 35174 | HMDB10378 | 5312531 | **3.14** | 1.38 | 0.79 | 1.67 | 1.63 | 0.83 | **2.15** | 1.70 | 0.99 |
| 15-methylpalmitate (i17:0) | LC/MS neg | 38768 |  | 17903417 | 1.46 | 1.10 | 1.21 | 1.06 | 1.54 | 1.00 | 1.40 | 1.44 | 1.28 |
| 17-methylstearate (i19:0) | LC/MS neg | 38296 | HMDB37397 | 3083779 | 1.30 | 0.95 | 1.19 | 0.89 | 1.41 | 0.86 | 1.26 | 1.12 | 1.26 |
| dimethylmalonic acid | LC/MS polar | 42978 | HMDB02001 | 11686 | 1.20 | **1.93** | 0.79 | 1.11 | 1.11 | **0.36** | 0.93 | 1.34 | 0.57 |
| glutarate (C5-DC) | LC/MS polar | 396 | HMDB00661 | 743 | 0.51 | **6.04** | **0.09** | 3.41 | 5.63 | 0.24 | 2.52 | 5.81 | 0.22 |
| 2-hydroxyglutarate | LC/MS polar | 37253 | HMDB00606 | 43 | 0.85 | 0.22 | 3.09 | **0.12** | 0.26 | 1.32 | **0.14** | 0.38 | 1.12 |
| maleate | LC/MS polar | 20676 | HMDB00176 | 444266 | 0.89 | 1.01 | 0.95 | 0.92 | 1.05 | 1.09 | 1.08 | 1.13 | 1.20 |
| N-linoleoylserine* | LC/MS neg | 61823 |  |  | 1.85 | 1.13 | 1.48 | 1.99 | 2.11 | 1.46 | **2.79** | 2.11 | 2.11 |
| butyrylcarnitine (C4) | LC/MS pos early | 32412 | HMDB02013 | 439829 | 0.98 | 0.99 | 1.00 | 0.97 | 0.98 | **1.11** | 0.99 | 1.04 | 1.05 |
| propionylcarnitine (C3) | LC/MS pos early | 32452 | HMDB00824 | 107738 | 0.92 | 1.04 | 1.03 | **0.83** | 0.93 | 1.01 | 0.92 | 1.03 | 1.00 |
| acetylcarnitine (C2) | LC/MS pos early | 32198 | HMDB00201 | 1 | 0.97 | 1.00 | 0.97 | 0.96 | 0.94 | 0.95 | 1.00 | 1.01 | **0.90** |
| 3-hydroxybutyrylcarnitine (1) | LC/MS pos early | 43264 | HMDB13127 | 53481617 | 1.09 | 0.83 | 1.00 | 1.06 | 1.08 | 0.98 | 0.98 | 1.20 | 0.83 |
| 3-hydroxybutyrylcarnitine (2) | LC/MS pos early | 52984 | HMDB13127 |  | 0.95 | 0.96 | 0.99 | 0.97 | 0.95 | 0.98 | 1.02 | 1.00 | 0.94 |
| hexanoylcarnitine (C6) | LC/MS pos early | 32328 | HMDB00705 | 6426853 | 0.87 | 0.97 | 1.01 | **0.69** | 1.03 | 1.02 | 0.79 | **1.32** | **1.38** |
| octanoylcarnitine (C8) | LC/MS pos late | 33936 | HMDB00791 | 123701 | 1.15 | 0.73 | 1.35 | 0.59 | 0.77 | 1.00 | **0.51** | 0.71 | 0.80 |
| 5-dodecenoylcarnitine (C12:1) | LC/MS pos late | 61769 |  |  | 0.95 | 0.76 | 1.44 | 0.68 | 0.51 | 1.61 | 0.68 | **0.32** | 1.22 |
| laurylcarnitine (C12) | LC/MS pos late | 34534 | HMDB02250 | 10427569 | 1.13 | 0.94 | 1.60 | 0.77 | 0.78 | 0.73 | 0.70 | **0.56** | 1.09 |
| myristoylcarnitine (C14) | LC/MS pos late | 33952 | HMDB05066 | 6426854 | 1.07 | 0.70 | 1.81 | 0.62 | 0.55 | 1.23 | **0.49** | 0.53 | 1.20 |
| palmitoylcarnitine (C16) | LC/MS pos late | 44681 | HMDB00222 | 461 | 0.80 | 0.71 | 1.75 | 0.55 | 0.60 | 1.20 | 0.51 | 0.61 | 1.12 |
| palmitoleoylcarnitine (C16:1)* | LC/MS pos late | 53223 |  | 71464547 | 1.70 | 0.71 | 1.59 | 0.67 | 0.53 | 0.84 | 0.52 | 0.44 | 0.98 |
| stearoylcarnitine (C18) | LC/MS pos late | 34409 | HMDB00848 | 6426855 | 0.61 | 0.89 | 1.63 | 0.62 | 0.64 | 1.40 | 0.60 | 0.77 | 0.85 |
| acetylcarnitine (C2) | LC/MS pos early | 32198 | HMDB00201 | 1 | 0.97 | 1.00 | 0.97 | 0.96 | 0.94 | 0.95 | 1.00 | 1.01 | **0.90** |
| linoleoylcarnitine (C18:2)* | LC/MS pos late | 46223 | HMDB06469 | 6450015 | **3.34** | 0.67 | 1.68 | 0.88 | 0.46 | 0.73 | 0.56 | 0.33 | 1.01 |
| 3-hydroxyoleoylcarnitine | LC/MS pos late | 61840 |  |  | 0.66 | 0.83 | 0.96 | 0.71 | 0.76 | 0.80 | 0.64 | **0.48** | 0.76 |
| oleoylcarnitine (C18:1) | LC/MS pos late | 35160 | HMDB05065 | 6441392 | 1.42 | 0.63 | 1.65 | 0.57 | 0.48 | 0.85 | 0.39 | 0.40 | 1.00 |
| myristoleoylcarnitine (C14:1)* | LC/MS pos late | 48182 |  | 90659872 | 1.06 | 0.77 | 1.48 | 0.79 | **0.60** | 0.92 | **0.59** | **0.49** | 0.96 |
| arachidonoylcarnitine (C20:4) | LC/MS pos late | 57518 |  |  | **2.98** | 0.77 | 1.32 | 0.74 | 0.63 | 0.88 | 0.65 | 0.60 | 0.91 |
| eicosenoylcarnitine (C20:1)* | LC/MS pos late | 57519 |  |  | 0.70 | 0.68 | 1.64 | 0.51 | 0.51 | 1.19 | 0.46 | 0.67 | 0.93 |
| deoxycarnitine | LC/MS pos early | 36747 | HMDB01161 | 134 | 0.95 | 1.05 | 0.97 | 0.95 | 0.94 | 0.96 | 1.06 | 1.07 | 0.92 |
| carnitine | LC/MS pos early | 15500 | HMDB00062 | 10917 | 0.84 | 1.15 | 0.86 | 1.10 | 1.06 | 1.05 | 1.20 | 1.23 | 0.92 |
| 4-hydroxybutyrate (GHB) | LC/MS polar | 34585 | HMDB00710 | 10413 | **1.24** | 0.96 | 1.03 | 0.88 | 0.96 | 1.04 | 0.92 | 1.03 | 1.10 |
| 2-hydroxypalmitate | LC/MS neg | 35675 | HMDB31057 | 92836 | 1.39 | 1.09 | 1.12 | 1.18 | 1.50 | 1.15 | **1.88** | 1.57 | 1.47 |
| 3-hydroxylaurate | LC/MS neg | 32457 | HMDB00387 | 94216 | **0.57** | 0.84 | 1.09 | **0.64** | **0.66** | 0.87 | **0.62** | 0.71 | 0.85 |
| 3-hydroxystearate | LC/MS neg | 52938 |  | 5282907 | 1.17 | 0.99 | 1.36 | 1.27 | 1.47 | 1.13 | 1.69 | 1.69 | 1.54 |
| N-oleoyltaurine | LC/MS neg | 39732 |  | 6437033 | 1.85 | 1.28 | 1.25 | 1.39 | 1.70 | 0.73 | 2.28 | 1.30 | 0.96 |
| N-stearoyltaurine | LC/MS neg | 39730 |  | 168274 | 1.25 | 1.00 | 0.98 | 0.84 | 2.03 | 0.96 | 1.38 | 1.96 | 0.79 |
| myo-inositol | LC/MS polar | 1124 | HMDB00211 | 892 | 1.01 | 1.22 | **0.55** | 1.17 | 1.17 | **0.65** | 1.15 | 1.14 | 0.84 |
| inositol 1-phosphate (I1P) | LC/MS polar | 43849 | HMDB00213 | 440194 | 0.92 | 1.00 | 1.05 | 0.93 | 1.05 | 0.92 | 1.07 | 1.10 | **0.80** |
| choline | LC/MS pos early | 15506 | HMDB00097 | 305 | 1.09 | 0.98 | 0.86 | 0.87 | 0.86 | **0.74** | 1.04 | 0.95 | **0.70** |
| choline phosphate | LC/MS pos early | 34396 | HMDB01565 | 1014 | 1.03 | 0.98 | 1.00 | 0.96 | 0.92 | **0.91** | 1.05 | 0.96 | **0.83** |
| cytidine 5'-diphosphocholine | LC/MS pos early | 34418 | HMDB01413 | 13804 | 1.08 | 0.99 | 1.12 | 1.10 | 0.97 | 1.19 | 1.21 | 1.09 | 1.13 |
| glycerophosphorylcholine (GPC) | LC/MS pos early | 15990 | HMDB00086 | 71920 | 1.07 | 1.10 | 0.95 | 1.01 | 0.97 | 0.97 | 1.07 | 1.06 | 1.04 |
| cytidine-5'-diphosphoethanolamine | LC/MS polar | 34410 | HMDB01564 | 123727 | 0.95 | 0.93 | 0.99 | 0.71 | 0.83 | **0.55** | 1.03 | 0.99 | **0.42** |
| glycerophosphoethanolamine | LC/MS polar | 37455 | HMDB00114 | 123874 | 1.05 | 1.09 | 0.96 | 1.02 | 0.94 | 0.91 | **1.15** | 1.02 | 0.95 |
| glycerophosphoserine* | LC/MS pos early | 57404 |  | 3081457 | 1.15 | 1.15 | 1.00 | 1.04 | 1.05 | 1.08 | 1.04 | **1.27** | 1.11 |
| glycerophosphoinositol* | LC/MS polar | 47155 |  | 167572 | 1.07 | 0.91 | 1.12 | 1.06 | 1.07 | **1.25** | **1.21** | 1.16 | 1.12 |
| 1-myristoyl-2-palmitoyl-GPC (14:0/16:0) | LC/MS pos late | 19258 | HMDB07869 | 129657 | 0.97 | 0.97 | 0.95 | 0.99 | 1.06 | 1.08 | 0.97 | 1.09 | 1.02 |
| 1-myristoyl-2-arachidonoyl-GPC (14:0/20:4)* | LC/MS pos late | 53195 | HMDB07883 |  | 1.01 | 1.09 | 0.91 | 1.23 | 1.18 | 1.21 | 1.21 | **1.30** | 1.04 |
| 1,2-dipalmitoyl-GPC (16:0/16:0) | LC/MS pos late | 19130 | HMDB00564 | 452110 | 0.97 | 0.94 | 0.99 | 0.95 | 1.00 | 1.12 | 0.94 | 1.03 | 1.01 |
| 1-palmitoyl-2-palmitoleoyl-GPC (16:0/16:1)* | LC/MS pos late | 52470 | HMDB07969 |  | 0.97 | 0.97 | 0.97 | 1.00 | 1.05 | 1.06 | 0.98 | 1.07 | 0.99 |
| 1-palmitoyl-2-stearoyl-GPC (16:0/18:0) | LC/MS pos late | 52616 | HMDB07970 |  | 0.95 | 1.03 | 0.99 | 0.90 | 1.04 | 1.11 | 0.91 | 1.04 | 0.98 |
| 1-palmitoyl-2-oleoyl-GPC (16:0/18:1) | LC/MS pos late | 52461 | HMDB07972 | 6436017 | 0.98 | 0.96 | 0.99 | 0.97 | 1.00 | 1.05 | 0.96 | 1.02 | 0.96 |
| 1-palmitoyl-2-arachidonoyl-GPC (16:0/20:4n6) | LC/MS pos late | 52462 | HMDB07982 | 10747814 | 0.96 | 1.10 | 0.94 | 1.12 | **1.27** | 1.19 | 1.15 | **1.31** | 1.04 |
| 1-palmitoyl-2-docosahexaenoyl-GPC (16:0/22:6) | LC/MS pos late | 52610 | HMDB07991 | 6441886 | 0.98 | 1.04 | 0.95 | 1.13 | 1.11 | 1.08 | 1.14 | 1.20 | 0.91 |
| 1,2-dipalmitoleoyl-GPC (16:1/16:1)* | LC/MS pos late | 52472 |  |  | 0.99 | 0.96 | 0.99 | 1.03 | 1.02 | 1.09 | 1.05 | 1.07 | 0.98 |
| 1-palmitoleoyl-2-oleoyl-GPC (16:1/18:1)* | LC/MS pos late | 52458 |  |  | 0.96 | 0.98 | 0.99 | 0.99 | 1.04 | 1.06 | 0.97 | 1.05 | 0.98 |
| 1-palmitoleoyl-2-linoleoyl-GPC (16:1/18:2)* | LC/MS pos late | 52683 | HMDB08006 |  | 1.01 | 0.94 | 1.02 | 1.10 | 1.07 | 1.03 | 1.10 | 1.13 | 0.91 |
| 1-palmitoleoyl-2-linolenoyl-GPC (16:1/18:3)* | LC/MS pos late | 53180 | HMDB08008 |  | 1.04 | 1.12 | 1.08 | 1.14 | 1.17 | 1.28 | 1.15 | 1.18 | 1.06 |
| 1-stearoyl-2-oleoyl-GPC (18:0/18:1) | LC/MS pos late | 52438 | HMDB08038 |  | 0.99 | 1.03 | 1.01 | 0.96 | 0.99 | 1.09 | 0.95 | 1.05 | 0.94 |
| 1-stearoyl-2-arachidonoyl-GPC (18:0/20:4) | LC/MS pos late | 42450 | HMDB08048 | 16219824 | 0.95 | 0.95 | 0.88 | 0.98 | 1.07 | 0.93 | 0.99 | 1.04 | **0.83** |
| 1-stearoyl-2-docosahexaenoyl-GPC (18:0/22:6) | LC/MS pos late | 52611 | HMDB08057 |  | 0.89 | 0.95 | 0.89 | **0.85** | 1.00 | 0.92 | 0.98 | 0.98 | **0.83** |
| 1,2-dioleoyl-GPC (18:1/18:1) | LC/MS pos late | 52457 |  | 10350317 | 0.97 | 0.97 | 1.01 | 0.98 | 1.00 | 1.07 | 0.97 | 1.03 | 0.99 |
| 1-oleoyl-2-linoleoyl-GPC (18:1/18:2)* | LC/MS pos late | 52453 |  |  | 0.95 | 0.96 | 0.96 | 0.91 | 0.99 | 0.96 | 0.92 | 1.01 | **0.88** |
| 1-oleoyl-2-docosahexaenoyl-GPC (18:1/22:6)* | LC/MS pos late | 52697 | HMDB08123 |  | 1.07 | 1.06 | 0.94 | 1.17 | 1.11 | 1.09 | 1.17 | 1.18 | 0.93 |
| 1,2-dilinoleoyl-GPC (18:2/18:2) | LC/MS pos late | 52603 | HMDB08138 | 5288075 | 1.05 | 1.00 | 1.03 | 1.13 | 1.04 | 1.10 | 1.11 | 1.12 | 0.93 |
| 1,2-dipalmitoyl-GPE (16:0/16:0)* | LC/MS pos late | 57341 | HMDB08923 | 445468 | 0.96 | 0.75 | 0.96 | 0.82 | **0.62** | 0.94 | 0.96 | **0.61** | 0.97 |
| 1-palmitoyl-2-oleoyl-GPE (16:0/18:1) | LC/MS pos late | 19263 | HMDB05320 | 5283496 | 0.97 | 1.00 | 0.98 | 0.98 | 1.08 | 1.09 | 0.98 | 1.10 | 1.01 |
| 1-palmitoyl-2-arachidonoyl-GPE (16:0/20:4)* | LC/MS pos late | 52464 | HMDB05323 | 9546800 | 0.95 | 1.03 | 1.05 | 1.00 | 1.03 | **1.18** | 1.01 | 1.10 | 1.06 |
| 1-palmitoyl-2-docosahexaenoyl-GPE (16:0/22:6)* | LC/MS pos late | 52465 | HMDB05324 | 9546799 | 0.98 | 1.03 | 0.90 | 0.95 | 1.08 | 1.02 | 0.94 | 1.04 | 0.94 |
| 1,2-dipalmitoleoyl-GPE (16:1/16:1)* | LC/MS pos late | 52688 | HMDB05342 | 9546809 | 1.01 | 1.02 | 1.02 | 1.08 | 1.08 | 1.16 | 1.05 | 1.11 | 1.05 |
| 1-palmitoleoyl-2-oleoyl-GPE (16:1/18:1)* | LC/MS pos late | 52469 |  |  | 0.99 | 1.02 | 1.01 | 1.01 | 1.08 | 1.13 | 1.01 | 1.08 | 1.05 |
| 1-stearoyl-2-oleoyl-GPE (18:0/18:1) | LC/MS pos late | 42448 | HMDB08993 |  | 0.96 | 0.99 | 1.03 | 1.01 | 1.05 | 1.17 | 0.99 | 1.10 | 1.06 |
| 1-stearoyl-2-arachidonoyl-GPE (18:0/20:4) | LC/MS pos late | 52447 | HMDB09003 | 5289133 | 1.00 | 0.97 | 0.96 | 1.00 | 1.03 | 1.11 | 0.97 | 1.06 | 0.99 |
| 1-stearoyl-2-docosahexaenoyl-GPE (18:0/22:6)* | LC/MS pos late | 52466 | HMDB05334 | 9546798 | 0.98 | 0.92 | 0.96 | 0.92 | 0.99 | 0.99 | 0.91 | 0.99 | 0.94 |
| 1,2-dioleoyl-GPE (18:1/18:1) | LC/MS pos late | 52609 |  | 9546757 | 0.98 | 1.00 | 0.97 | 0.97 | 1.06 | 1.08 | 0.97 | 1.09 | 0.98 |
| 1-oleoyl-2-linoleoyl-GPE (18:1/18:2)* | LC/MS pos late | 52687 | HMDB05349 | 9546753 | 0.95 | 1.00 | 0.99 | 0.99 | 1.03 | 1.09 | 0.97 | 1.04 | 1.01 |
| 1-oleoyl-2-arachidonoyl-GPE (18:1/20:4)* | LC/MS pos late | 55041 | HMDB09069 |  | 1.00 | 1.01 | 1.00 | 1.01 | 1.05 | 1.13 | 1.02 | 1.10 | 0.98 |
| 1-palmitoyl-2-oleoyl-GPS (16:0/18:1) | LC/MS pos late | 19261 | HMDB12357 | 5283499 | 1.00 | 1.01 | 1.00 | 1.06 | 1.12 | **1.24** | 1.02 | **1.17** | 1.13 |
| 1-stearoyl-2-oleoyl-GPS (18:0/18:1) | LC/MS pos late | 19265 | HMDB10163 | 9547087 | 0.97 | 0.99 | 1.02 | 0.97 | 1.05 | 1.15 | 0.94 | 1.11 | 1.00 |
| 1-stearoyl-2-arachidonoyl-GPS (18:0/20:4) | LC/MS pos late | 52235 | HMDB12383 |  | 0.97 | 1.04 | 1.08 | 0.98 | 1.10 | **1.31** | 0.95 | 1.10 | 1.13 |
| 1,2-dioleoyl-GPS (18:1/18:1) | LC/MS pos late | 19191 |  | 6438639 | 1.00 | **0.84** | 1.01 | 1.04 | 1.06 | 1.14 | 1.01 | 1.13 | 1.03 |
| 1,2-dipalmitoyl-GPG (16:0/16:0) | LC/MS pos late | 52625 | HMDB10570 | 11846227 | 0.96 | 1.08 | 1.09 | 0.88 | 1.04 | 1.21 | 0.93 | 1.03 | 0.98 |
| 1-palmitoyl-2-oleoyl-GPG (16:0/18:1) | LC/MS pos late | 52448 |  | 5283509 | 1.03 | 1.06 | 1.05 | 1.00 | 1.05 | 1.18 | 1.05 | 1.10 | 0.99 |
| 1-stearoyl-2-oleoyl-GPG (18:0/18:1) | LC/MS pos late | 52623 |  |  | 0.99 | 1.02 | 1.00 | 0.99 | 1.00 | 1.18 | 0.98 | 1.14 | 1.04 |
| 1,2-dioleoyl-GPG (18:1/18:1) | LC/MS polar | 19142 |  | 11846228 | **2.27** | 1.05 | **1.74** | **2.13** | 1.06 | **1.63** | 1.14 | 1.14 | 1.04 |
| 1,2-dipalmitoyl-GPI (16:0/16:0)* | LC/MS pos late | 57375 | HMDB09778 |  | 0.64 | 0.96 | 1.05 | 0.62 | 1.22 | 0.94 | 0.61 | 1.57 | 1.14 |
| 1-palmitoyl-2-oleoyl-GPI (16:0/18:1)* | LC/MS pos late | 52669 | HMDB09783 |  | 1.00 | 0.94 | 1.10 | 0.95 | 1.06 | **1.23** | 0.96 | 1.10 | 1.09 |
| 1-palmitoleoyl-2-oleoyl-GPI (16:1/18:1)* | LC/MS pos late | 52725 |  |  | 0.95 | 1.01 | 1.10 | 1.02 | 1.08 | **1.27** | 0.99 | 1.15 | 1.14 |
| 1-palmitoyl-2-arachidonoyl-GPI (16:0/20:4)* | LC/MS pos late | 52467 | HMDB09789 |  | 1.01 | 0.93 | 1.00 | 0.93 | 1.02 | 1.09 | 0.95 | 1.03 | 0.88 |
| 1-stearoyl-2-oleoyl-GPI (18:0/18:1)* | LC/MS pos late | 52726 |  |  | 0.97 | 0.99 | 1.11 | 0.95 | 1.03 | **1.21** | 0.95 | 1.12 | 1.06 |
| 1,2-dioleoyl-GPI (18:1/18:1) | LC/MS pos late | 52619 | HMDB09837 |  | 0.98 | 0.98 | 1.11 | 0.96 | 1.06 | 1.20 | 0.86 | 1.08 | 1.08 |
| 1-stearoyl-2-arachidonoyl-GPI (18:0/20:4) | LC/MS pos late | 52449 | HMDB09815 |  | 1.07 | 0.96 | 1.02 | 0.93 | 0.95 | 0.99 | 0.92 | 1.05 | **0.80** |
| 1-oleoyl-2-arachidonoyl-GPI (18:1/20:4) * | LC/MS pos late | 54994 | HMDB09844 |  | 0.98 | 0.93 | 1.02 | 0.89 | 0.98 | 1.02 | 0.91 | 1.01 | 0.90 |
| 1-palmitoyl-GPC (16:0) | LC/MS pos late | 33955 | HMDB10382 | 86554 | 1.19 | 1.09 | 1.09 | 1.15 | 1.05 | 1.16 | 1.11 | 1.12 | 1.01 |
| 2-palmitoyl-GPC (16:0)* | LC/MS neg | 35253 | HMDB61702 | 15061532 | 1.18 | 1.40 | 0.86 | 1.08 | **2.20** | 0.76 | 1.32 | **2.05** | 1.27 |
| 1-palmitoleoyl-GPC (16:1)* | LC/MS pos late | 33230 | HMDB10383 | 24779461 | 1.08 | 1.06 | 0.97 | 1.22 | 1.09 | 1.19 | 1.13 | 1.23 | 1.01 |
| 2-palmitoleoyl-GPC (16:1)* | LC/MS pos late | 35819 | HMDB10383 |  | 0.98 | 1.06 | 1.00 | 0.92 | 1.13 | **1.27** | 0.99 | 1.20 | 1.09 |
| 1-stearoyl-GPC (18:0) | LC/MS pos late | 33961 | HMDB10384 | 497299 | 1.28 | 1.10 | 1.04 | 1.22 | 1.10 | 1.22 | 1.20 | 1.19 | 0.97 |
| 1-oleoyl-GPC (18:1) | LC/MS pos late | 48258 | HMDB02815 | 16081932 | 1.12 | 1.13 | 1.03 | 1.20 | 1.16 | 1.17 | 1.15 | 1.21 | 1.00 |
| 1-lignoceroyl-GPC (24:0) | LC/MS pos late | 49617 | HMDB10405 |  | 0.76 | 1.02 | 1.41 | 0.88 | 1.09 | 1.15 | 0.90 | 1.01 | 1.12 |
| 1-palmitoyl-GPE (16:0) | LC/MS pos late | 35631 | HMDB11503 | 9547069 | **1.37** | 1.09 | 0.91 | 1.28 | 1.26 | 1.13 | 1.20 | 1.28 | 0.97 |
| 1-stearoyl-GPE (18:0) | LC/MS pos late | 42398 | HMDB11130 | 9547068 | 1.26 | 1.21 | 0.84 | **1.43** | 1.20 | 1.00 | 1.19 | 1.25 | 0.89 |
| 2-stearoyl-GPE (18:0)* | LC/MS neg | 41220 | HMDB11129 |  | 0.98 | 1.75 | 1.11 | 1.39 | 1.50 | 0.77 | 1.23 | 1.41 | 0.81 |
| 1-oleoyl-GPE (18:1) | LC/MS neg | 35628 | HMDB11506 | 9547071 | 1.75 | 1.15 | 1.13 | 1.32 | 1.67 | 0.74 | 1.32 | 1.63 | 1.01 |
| 1-linoleoyl-GPE (18:2)* | LC/MS neg | 32635 | HMDB11507 | 52925130 | **2.65** | 1.20 | 0.97 | 0.80 | 1.00 | 0.95 | 1.68 | 1.73 | 1.16 |
| 1-arachidonoyl-GPE (20:4n6)* | LC/MS neg | 35186 | HMDB11517 | 42607465 | 0.98 | 1.58 | 0.78 | 0.85 | **1.96** | 0.61 | 1.05 | **2.04** | 0.84 |
| 1-palmitoyl-GPS (16:0)* | LC/MS neg | 46130 |  | 9547100 | 1.56 | 1.85 | 1.23 | 1.49 | **2.69** | 1.18 | 1.79 | **2.81** | 1.86 |
| 1-stearoyl-GPS (18:0)* | LC/MS neg | 45966 |  | 9547101 | 1.68 | 1.25 | 1.01 | 1.38 | 1.73 | 0.82 | 1.41 | 1.82 | 1.14 |
| 1-oleoyl-GPS (18:1) | LC/MS neg | 19260 | HMDB61694 | 9547099 | 1.83 | 1.51 | 0.80 | 1.47 | 1.86 | 0.71 | 1.65 | **2.00** | 1.10 |
| 1-palmitoyl-GPG (16:0)* | LC/MS neg | 45970 |  | 3300276 | 1.40 | 1.38 | 0.97 | 1.06 | 1.48 | 0.82 | 1.23 | 1.51 | 1.01 |
| 1-stearoyl-GPG (18:0) | LC/MS neg | 34437 |  |  | 1.33 | 1.22 | 0.99 | 1.03 | 1.45 | 0.77 | 1.24 | 1.59 | 1.11 |
| 1-oleoyl-GPG (18:1)* | LC/MS neg | 45968 |  |  | 1.42 | 1.39 | 0.92 | 0.99 | **1.68** | 0.76 | 1.34 | **1.70** | 1.03 |
| 1-linoleoyl-GPG (18:2)* | LC/MS neg | 54885 |  |  | 1.03 | 1.47 | 1.16 | 1.03 | 1.40 | 0.90 | 1.49 | 1.75 | 1.46 |
| 1-palmitoyl-GPI (16:0) | LC/MS neg | 35305 | HMDB61695 |  | 1.01 | 1.35 | 1.09 | 1.37 | **2.20** | 1.12 | **1.91** | **2.32** | 1.21 |
| 1-stearoyl-GPI (18:0) | LC/MS neg | 19324 | HMDB61696 |  | 1.16 | 1.17 | 1.10 | 1.28 | **2.14** | 1.13 | **1.82** | **2.32** | 1.11 |
| 1-oleoyl-GPI (18:1)* | LC/MS neg | 36602 |  |  | 1.35 | 1.25 | 1.10 | 1.28 | **2.16** | 1.14 | **1.73** | **2.27** | 1.19 |
| 1-arachidonoyl-GPI (20:4)* | LC/MS neg | 34214 | HMDB61690 |  | **2.03** | 1.21 | **0.55** | 1.15 | **1.77** | 0.71 | 1.53 | **2.17** | 0.70 |
| galactosylglycerol* | LC/MS polar | 57345 | HMDB06790 | 16048618 | 0.99 | 0.99 | 1.00 | 0.96 | 0.92 | 0.97 | 1.06 | 0.96 | 0.97 |
| 1-(1-enyl-palmitoyl)-2-oleoyl-GPE (P-16:0/18:1)* | LC/MS pos late | 52477 | HMDB11342 |  | 0.99 | 1.04 | 1.02 | 0.96 | 1.00 | **1.14** | 0.96 | 1.08 | 1.01 |
| 1-(1-enyl-palmitoyl)-2-linoleoyl-GPE (P-16:0/18:2)* | LC/MS pos late | 52677 | HMDB11343 |  | 0.92 | 1.00 | 1.00 | **0.84** | 0.94 | 1.01 | **0.86** | 1.03 | 0.93 |
| 1-(1-enyl-palmitoyl)-2-palmitoyl-GPC (P-16:0/16:0)* | LC/MS pos late | 52716 | HMDB11206 | 11146967 | 0.96 | 1.03 | 0.97 | 0.90 | 1.03 | 1.06 | 0.92 | 1.03 | 0.94 |
| 1-(1-enyl-palmitoyl)-2-palmitoleoyl-GPC (P-16:0/16:1)* | LC/MS pos late | 52713 | HMDB11207 |  | 0.97 | 0.99 | 1.00 | 0.95 | 1.00 | 1.08 | 0.98 | 1.04 | 0.97 |
| 1-(1-enyl-palmitoyl)-2-arachidonoyl-GPE (P-16:0/20:4)* | LC/MS pos late | 52673 | HMDB11352 |  | 0.97 | 1.01 | 0.99 | 0.97 | 1.02 | 1.03 | 0.95 | 1.04 | 0.97 |
| 1-(1-enyl-palmitoyl)-2-oleoyl-GPC (P-16:0/18:1)* | LC/MS pos late | 52478 |  |  | 0.98 | 1.02 | 1.01 | 0.95 | 1.02 | 1.09 | 0.96 | 1.04 | 0.99 |
| 1-(1-enyl-stearoyl)-2-oleoyl-GPE (P-18:0/18:1) | LC/MS pos late | 52614 | HMDB11375 |  | 0.99 | 1.05 | 1.01 | 0.95 | 1.04 | 1.09 | 1.00 | 1.15 | 0.93 |
| 1-(1-enyl-palmitoyl)-2-arachidonoyl-GPC (P-16:0/20:4)* | LC/MS pos late | 52689 | HMDB11220 |  | 0.95 | 0.95 | 1.01 | 0.93 | 0.89 | 1.06 | 0.95 | **0.74** | **0.67** |
| 1-(1-enyl-palmitoyl)-2-linoleoyl-GPC (P-16:0/18:2)* | LC/MS pos late | 52682 | HMDB11211 |  | 0.97 | 0.97 | 1.00 | 0.92 | 0.98 | 1.07 | 0.94 | 1.01 | 0.96 |
| 1-(1-enyl-stearoyl)-2-arachidonoyl-GPE (P-18:0/20:4)* | LC/MS pos late | 52475 | HMDB05779 | 9547058 | 0.98 | 1.06 | 0.98 | 0.94 | 1.06 | 1.01 | 0.95 | 1.08 | 0.92 |
| 1-(1-enyl-palmitoyl)-GPE (P-16:0)* | LC/MS pos late | 39270 |  |  | **1.53** | 1.23 | 0.87 | **1.53** | 1.21 | 0.94 | 1.32 | 1.30 | 0.87 |
| 1-(1-enyl-oleoyl)-GPE (P-18:1)* | LC/MS pos late | 44621 |  |  | **1.89** | 1.24 | 0.79 | **1.52** | 1.15 | 0.86 | **1.36** | 1.25 | 0.82 |
| 1-(1-enyl-stearoyl)-GPE (P-18:0)* | LC/MS pos late | 39271 |  |  | 1.35 | 1.23 | 0.89 | **1.50** | 1.15 | 1.01 | 1.30 | 1.28 | 0.91 |
| 1-(1-enyl-oleoyl)-2-oleoyl-GPE (P-18:1/18:1)* | LC/MS pos late | 54691 | HMDB11441 |  | 1.00 | 1.02 | 1.04 | 0.97 | 1.01 | **1.15** | 0.96 | 1.08 | 1.01 |
| glycerol | LC/MS neg | 15122 | HMDB00131 | 753 | 1.03 | 1.08 | 0.88 | 0.89 | 1.15 | 0.90 | 1.10 | 1.04 | 0.73 |
| glycerol 3-phosphate | LC/MS pos early | 43847 | HMDB00126 | 754 | **1.80** | 1.00 | 0.74 | 0.93 | 0.88 | 0.74 | 0.81 | 0.85 | 0.72 |
| glycerophosphoglycerol | LC/MS polar | 48857 |  | 439964 | 1.08 | 1.07 | 0.94 | 1.05 | 1.09 | **1.17** | **1.16** | **1.15** | **1.22** |
| 1-myristoylglycerol (14:0) | LC/MS neg | 35625 | HMDB11561 | 79050 | 1.86 | 0.99 | 1.05 | 0.95 | 1.19 | 0.55 | 1.24 | 1.11 | 0.91 |
| 1-pentadecanoylglycerol (15:0) | LC/MS neg | 47898 |  | 190750 | **2.35** | 1.89 | 1.17 | 1.12 | 1.51 | 0.72 | **2.31** | 1.24 | 1.02 |
| 1-palmitoleoylglycerol (16:1)* | LC/MS neg | 52431 | HMDB11565 |  | **2.69** | 1.04 | 1.00 | 1.11 | 1.21 | 0.41 | 1.50 | 1.03 | 0.79 |
| 1-margaroylglycerol (17:0) | LC/MS neg | 34391 |  | 107036 | 2.47 | 0.89 | **0.39** | 1.43 | 1.79 | **0.28** | 1.47 | 1.44 | **0.24** |
| 1-oleoylglycerol (18:1) | LC/MS neg | 21184 | HMDB11567 | 5283468 | 2.19 | 0.69 | 0.67 | 0.82 | 0.83 | 0.42 | 1.43 | 0.82 | 0.62 |
| 1-dihomo-linolenylglycerol (20:3) | LC/MS neg | 48341 |  |  | 1.96 | 1.11 | 0.51 | 0.59 | 0.97 | **0.32** | 1.22 | 0.88 | 0.55 |
| 2-myristoylglycerol (14:0) | LC/MS neg | 34383 | HMDB11530 | 137938 | **2.22** | 1.16 | 1.07 | 1.13 | 1.16 | 0.52 | 1.56 | 1.09 | 0.95 |
| 2-palmitoylglycerol (16:0) | LC/MS neg | 33419 | HMDB11533 | 123409 | **2.66** | 1.48 | 0.92 | 0.97 | 1.74 | 0.63 | 1.71 | 1.34 | 0.90 |
| 2-palmitoleoylglycerol (16:1)* | LC/MS neg | 52432 | HMDB11565 |  | **2.74** | 1.05 | 1.01 | 1.22 | 1.21 | 0.43 | 1.59 | 1.08 | 0.80 |
| 2-oleoylglycerol (18:1) | LC/MS neg | 21232 | HMDB11537 | 5319879 | 1.92 | 0.93 | 0.75 | 0.67 | 0.89 | **0.42** | 1.14 | 1.09 | 0.73 |
| diacylglycerol (12:0/18:1, 14:0/16:1, 16:0/14:1) [1]* | LC/MS pos late | 55002 |  |  | 1.16 | 0.71 | 0.83 | 1.13 | 0.93 | 0.78 | 1.11 | 0.98 | 1.02 |
| diacylglycerol (12:0/18:1, 14:0/16:1, 16:0/14:1) [2]* | LC/MS pos late | 55001 |  |  | 1.24 | 0.96 | 0.98 | 1.24 | 0.89 | 0.99 | 1.01 | 1.00 | 0.85 |
| diacylglycerol (14:0/18:1, 16:0/16:1) [1]* | LC/MS pos late | 54953 |  |  | 1.21 | 1.01 | 0.97 | 1.00 | 0.99 | 0.81 | 1.02 | 0.92 | 0.89 |
| diacylglycerol (14:0/18:1, 16:0/16:1) [2]* | LC/MS pos late | 54954 |  |  | 1.20 | 0.98 | 1.07 | 1.11 | 1.08 | 1.11 | 1.14 | 1.17 | 1.07 |
| diacylglycerol (16:1/18:2 [2], 16:0/18:3 [1])* | LC/MS pos late | 54966 |  |  | 1.33 | 1.04 | 1.00 | 1.15 | 0.87 | 0.82 | 0.93 | 0.95 | 0.89 |
| palmitoyl-myristoyl-glycerol (16:0/14:0) [1]* | LC/MS pos late | 61709 |  |  | 1.55 | 0.94 | 1.25 | 1.23 | **1.80** | 1.05 | 1.56 | 1.04 | 1.60 |
| palmitoyl-myristoyl-glycerol (16:0/14:0) [2] | LC/MS pos late | 57364 | HMDB07095 |  | 0.99 | 1.08 | 1.22 | 1.01 | 1.18 | **1.54** | 1.09 | **1.36** | **1.38** |
| palmitoyl-palmitoyl-glycerol (16:0/16:0) [2]* | LC/MS pos late | 54990 | HMDB07098 |  | 0.94 | 1.04 | 1.06 | 0.85 | 1.30 | 1.26 | 0.91 | **1.64** | 1.15 |
| palmitoleoyl-palmitoleoyl-glycerol (16:1/16:1) [2]* | LC/MS pos late | 57409 |  |  | 1.06 | 0.94 | 0.94 | 1.10 | 0.85 | 0.81 | 0.91 | 0.90 | 0.78 |
| palmitoyl-oleoyl-glycerol (16:0/18:1) [1]* | LC/MS pos late | 54943 | HMDB07102 |  | 1.00 | 1.13 | 0.78 | 1.51 | 1.95 | 1.56 | 2.75 | 3.00 | 2.60 |
| palmitoyl-oleoyl-glycerol (16:0/18:1) [2]* | LC/MS pos late | 54942 | HMDB07102 |  | 1.25 | 1.02 | 1.01 | 1.09 | 1.11 | 1.10 | 1.14 | **1.32** | 1.03 |
| palmitoleoyl-oleoyl-glycerol (16:1/18:1) [1]* | LC/MS pos late | 52632 | HMDB07131 | 9543694 | 1.07 | 0.90 | 0.92 | 0.94 | 0.93 | **0.69** | 0.98 | 0.92 | **0.76** |
| palmitoleoyl-oleoyl-glycerol (16:1/18:1) [2]* | LC/MS pos late | 52631 |  |  | 1.17 | 0.95 | 1.02 | 1.06 | 0.97 | 0.91 | 1.06 | 1.04 | 0.91 |
| palmitoyl-dihomo-linolenoyl-glycerol (16:0/20:3n3 or 6) [2]* | LC/MS pos late | 54941 |  |  | 0.70 | 0.76 | 1.16 | 1.16 | 0.96 | 0.55 | 0.57 | 1.34 | 0.64 |
| palmitoyl-arachidonoyl-glycerol (16:0/20:4) [2]* | LC/MS pos late | 54958 | HMDB07112 |  | 1.21 | 1.07 | 0.92 | 1.07 | 0.98 | 0.85 | 1.08 | 1.11 | **0.68** |
| oleoyl-oleoyl-glycerol (18:1/18:1) [2]* | LC/MS pos late | 54946 | HMDB07218 |  | 1.26 | 0.97 | 1.04 | 1.04 | 0.98 | 0.94 | 1.10 | 1.13 | 0.92 |
| oleoyl-linoleoyl-glycerol (18:1/18:2) [1] | LC/MS pos late | 46798 | HMDB07219 |  | 1.20 | 0.70 | **0.44** | 1.29 | 0.96 | **0.43** | 1.20 | **0.56** | **0.43** |
| oleoyl-linoleoyl-glycerol (18:1/18:2) [2] | LC/MS pos late | 46799 | HMDB07219 |  | 1.27 | 0.90 | 0.97 | 1.11 | 1.03 | 0.86 | 1.09 | 1.01 | 0.92 |
| stearoyl-arachidonoyl-glycerol (18:0/20:4) [2]* | LC/MS pos late | 57449 |  |  | 1.20 | 0.94 | 0.91 | 1.16 | 0.97 | 0.82 | 1.33 | 1.01 | **0.65** |
| oleoyl-arachidonoyl-glycerol (18:1/20:4) [2]* | LC/MS pos late | 54961 | HMDB07228 |  | **1.31** | 0.86 | 0.99 | 1.07 | 0.95 | 0.82 | 0.96 | 0.88 | **0.75** |
| stearoyl-docosahexaenoyl-glycerol (18:0/22:6) [2]* | LC/MS pos late | 57368 |  |  | 1.22 | 1.19 | 0.93 | 1.13 | 1.24 | 0.99 | 1.18 | **1.43** | **0.71** |
| sphinganine | LC/MS pos late | 17769 | HMDB00269 | 3126 | **0.75** | 0.89 | **1.29** | **0.80** | 0.94 | 1.20 | **0.79** | 0.99 | 1.11 |
| sphingadienine | LC/MS pos late | 57426 |  | 6449795 | **2.87** | 1.25 | 0.96 | 1.62 | 1.26 | 0.67 | 1.46 | 1.26 | 0.78 |
| phytosphingosine | LC/MS pos late | 1510 | HMDB04610 | 122121 | **1.47** | 1.16 | 0.98 | 1.20 | 1.09 | 0.87 | 1.21 | 1.20 | 0.89 |
| N-palmitoyl-sphinganine (d18:0/16:0) | LC/MS pos late | 52604 | HMDB11760 | 5283572 | 0.88 | 0.90 | 1.08 | 1.12 | 1.12 | **1.37** | 1.10 | 1.22 | 1.18 |
| N-palmitoyl-sphingosine (d18:1/16:0) | LC/MS pos late | 44877 | HMDB04949 | 5283564 | 0.91 | 0.95 | 1.00 | 0.88 | 1.00 | 1.14 | 1.01 | 1.14 | 1.12 |
| N-stearoyl-sphingosine (d18:1/18:0)* | LC/MS pos late | 54979 | HMDB04950 | 5283565 | 0.83 | 1.03 | 1.01 | 0.87 | 1.02 | 1.26 | 0.88 | 1.21 | 0.93 |
| N-palmitoyl-sphingadienine (d18:2/16:0)* | LC/MS pos late | 57416 |  |  | 0.88 | 0.89 | 0.97 | **0.69** | **0.83** | 0.89 | **0.77** | **0.83** | 0.89 |
| N-behenoyl-sphingadienine (d18:2/22:0)* | LC/MS pos late | 57372 |  |  | 2.22 | 1.18 | 0.46 | **2.37** | 0.99 | 1.21 | **2.55** | 1.00 | 1.17 |
| N-nervonoyl-sphingadiene (d18:2/24:1)* | LC/MS pos late | 57442 |  |  | 0.95 | 1.05 | 1.04 | 0.89 | 0.93 | 1.18 | 1.03 | 1.09 | 1.11 |
| N-palmitoyl-heptadecasphingosine (d17:1/16:0)* | LC/MS pos late | 57430 |  |  | 0.94 | 1.03 | 1.01 | 0.96 | 1.00 | 1.04 | 1.12 | 1.15 | 0.98 |
| ceramide (d18:1/14:0, d16:1/16:0)* | LC/MS pos late | 57432 |  |  | **0.82** | 0.86 | 1.00 | **0.71** | **0.85** | 1.04 | **0.76** | **0.85** | 0.95 |
| ceramide (d18:1/20:0, d16:1/22:0, d20:1/18:0)* | LC/MS pos late | 57440 |  |  | **0.66** | 1.20 | 0.93 | 0.77 | 1.13 | 1.00 | 0.93 | 1.29 | 0.88 |
| ceramide (d16:1/24:1, d18:1/22:1)* | LC/MS pos late | 57437 |  |  | 0.97 | 0.96 | 1.01 | 0.90 | 0.90 | 1.11 | 1.07 | 1.02 | 1.01 |
| glycosyl-N-palmitoyl-sphingosine (d18:1/16:0) | LC/MS pos late | 53013 |  |  | 0.95 | 0.99 | 0.93 | 0.91 | 1.03 | 0.97 | 0.96 | 1.08 | 0.90 |
| glycosyl-N-stearoyl-sphingosine (d18:1/18:0) | LC/MS pos late | 52234 |  |  | 0.99 | 1.18 | 0.97 | 0.97 | 1.23 | 1.09 | 1.09 | **1.36** | 0.87 |
| glycosyl-N-behenoyl-sphingadienine (d18:2/22:0)* | LC/MS pos late | 57421 |  |  | 0.99 | 1.11 | 0.97 | 0.96 | 1.14 | 1.18 | 1.03 | **1.24** | 0.93 |
| glycosyl-N-nervonoyl-sphingadienine (d18:2/24:1)* | LC/MS pos late | 57451 |  |  | 1.00 | 1.02 | 1.03 | 0.98 | 1.08 | 1.17 | 1.03 | 1.19 | 1.01 |
| glycosyl ceramide (d18:1/20:0, d16:1/22:0)* | LC/MS pos late | 57595 |  |  | 0.96 | 1.14 | 0.97 | 0.86 | **1.28** | **1.29** | 0.97 | **1.36** | 0.97 |
| glycosyl ceramide (d16:1/24:1, d18:1/22:1)* | LC/MS pos late | 57457 |  |  | 0.90 | 1.00 | 0.97 | 0.96 | 1.18 | 1.17 | 0.95 | 1.17 | 1.05 |
| glycosyl ceramide (d18:1/23:1, d17:1/24:1)* | LC/MS pos late | 57448 |  |  | 0.97 | 1.05 | 0.99 | 0.99 | 1.01 | 1.09 | 1.11 | **1.25** | 0.96 |
| lactosyl-N-palmitoyl-sphingosine (d18:1/16:0) | LC/MS pos late | 53010 |  |  | 1.06 | 1.02 | 1.07 | 1.07 | 1.08 | **1.22** | 1.02 | 1.08 | 1.04 |
| lactosyl-N-nervonoyl-sphingosine (d18:1/24:1)* | LC/MS pos late | 57370 |  |  | 1.03 | 1.05 | 0.96 | 1.06 | 1.10 | 1.15 | 1.09 | 1.15 | 0.95 |
| myristoyl dihydrosphingomyelin (d18:0/14:0)* | LC/MS pos late | 57365 | HMDB12085 |  | 1.07 | 1.02 | 0.99 | 1.20 | **1.37** | **1.46** | **1.35** | **1.36** | 1.16 |
| palmitoyl dihydrosphingomyelin (d18:0/16:0)* | LC/MS pos late | 52434 |  | 9939965 | 0.99 | 0.97 | 0.96 | 1.12 | **1.21** | **1.29** | **1.15** | **1.20** | **1.17** |
| behenoyl dihydrosphingomyelin (d18:0/22:0)* | LC/MS pos late | 57331 | HMDB12091 |  | 1.17 | 1.01 | 1.05 | 1.55 | 1.45 | **2.07** | 1.37 | **1.67** | **1.66** |
| sphingomyelin (d18:0/20:0, d16:0/22:0)* | LC/MS pos late | 57476 |  |  | 1.59 | 1.56 | 1.05 | 1.92 | 1.94 | **2.35** | **2.41** | **2.26** | 1.66 |
| palmitoyl sphingomyelin (d18:1/16:0) | LC/MS pos late | 37506 |  | 9939941 | 0.96 | 0.98 | 0.96 | 0.96 | 1.04 | 1.07 | 0.96 | 1.09 | 1.02 |
| stearoyl sphingomyelin (d18:1/18:0) | LC/MS pos late | 19503 | HMDB01348 | 6453725 | 0.94 | 1.03 | 1.00 | 0.91 | 0.97 | 1.08 | 0.88 | 0.98 | 0.92 |
| behenoyl sphingomyelin (d18:1/22:0)* | LC/MS pos late | 48492 | HMDB12103 |  | 0.93 | 1.17 | 0.86 | 0.80 | 1.16 | 1.01 | 1.03 | **1.31** | 0.87 |
| tricosanoyl sphingomyelin (d18:1/23:0)* | LC/MS pos late | 52436 | HMDB12105 |  | 0.99 | 1.25 | 0.96 | 0.89 | 1.26 | 1.13 | 1.04 | **1.45** | 0.91 |
| lignoceroyl sphingomyelin (d18:1/24:0) | LC/MS pos late | 57330 |  |  | 0.92 | 1.15 | 1.01 | 0.86 | 1.15 | 1.15 | 1.01 | **1.32** | 0.97 |
| sphingomyelin (d18:2/23:1)* | LC/MS pos late | 57482 |  |  | 1.05 | 1.06 | 1.03 | 0.94 | 1.18 | 1.02 | 1.00 | 1.01 | 0.87 |
| sphingomyelin (d18:2/24:2)* | LC/MS pos late | 57479 |  |  | 1.01 | 0.83 | 0.87 | 0.90 | 0.77 | 0.86 | 1.08 | 1.04 | 1.26 |
| sphingomyelin (d17:1/14:0, d16:1/15:0)* | LC/MS pos late | 62152 |  |  | 1.21 | 1.08 | 1.12 | 1.28 | 1.16 | **1.50** | 1.29 | **1.32** | 1.27 |
| sphingomyelin (d18:1/14:0, d16:1/16:0)* | LC/MS pos late | 42463 | HMDB12097 | 11433862 | 1.00 | 0.99 | 0.94 | 1.02 | 1.05 | 1.11 | 1.03 | 1.10 | 1.01 |
| sphingomyelin (d18:2/14:0, d18:1/14:1)* | LC/MS pos late | 47154 |  |  | 1.13 | 1.00 | 1.12 | 1.20 | 1.09 | **1.45** | 1.24 | 1.30 | 1.23 |
| sphingomyelin (d17:1/16:0, d18:1/15:0, d16:1/17:0)* | LC/MS pos late | 52433 |  |  | 1.07 | 1.07 | 1.00 | 1.12 | 1.17 | 1.20 | 1.17 | 1.21 | 1.03 |
| sphingomyelin (d18:2/16:0, d18:1/16:1)* | LC/MS pos late | 42459 |  |  | 0.99 | 0.96 | 1.00 | 1.00 | 1.02 | 1.12 | 1.01 | 1.08 | 1.01 |
| sphingomyelin (d18:1/20:0, d16:1/22:0)* | LC/MS pos late | 48490 | HMDB12102 |  | 0.95 | 1.03 | 1.00 | 0.94 | 1.03 | **1.19** | 0.94 | 1.08 | 1.00 |
| sphingomyelin (d18:1/21:0, d17:1/22:0, d16:1/23:0)* | LC/MS pos late | 52495 |  |  | 1.02 | 1.17 | 1.14 | 1.24 | 1.38 | **1.53** | 1.29 | 1.49 | 1.26 |
| sphingomyelin (d18:1/22:1, d18:2/22:0, d16:1/24:1)* | LC/MS pos late | 48493 | HMDB12104 |  | 0.99 | 1.06 | 1.00 | 1.01 | 1.13 | **1.22** | 1.03 | 1.13 | 1.05 |
| sphingomyelin (d18:1/22:2, d18:2/22:1, d16:1/24:2)* | LC/MS pos late | 57477 |  |  | 1.12 | 1.13 | 0.79 | 1.10 | 1.25 | 1.03 | 1.08 | **1.40** | 1.01 |
| sphingomyelin (d18:2/23:0, d18:1/23:1, d17:1/24:1)* | LC/MS pos late | 52435 |  |  | 1.07 | 1.15 | 1.01 | 1.16 | 1.30 | 1.40 | 1.28 | **1.48** | 1.17 |
| sphingomyelin (d18:1/24:1, d18:2/24:0)* | LC/MS pos late | 47153 | HMDB12107 |  | 0.96 | 1.16 | 0.92 | 1.00 | **1.27** | 1.16 | 1.10 | **1.38** | 1.02 |
| sphingomyelin (d18:2/24:1, d18:1/24:2)* | LC/MS pos late | 52437 |  |  | 0.99 | 1.06 | 1.01 | 0.99 | 1.11 | **1.21** | 1.02 | 1.13 | 1.05 |
| sphingomyelin (d18:1/25:0, d19:0/24:1, d20:1/23:0, d19:1/24:0)* | LC/MS pos late | 57478 |  |  | 0.95 | 1.13 | 0.95 | 0.98 | 1.16 | 1.19 | 0.95 | **1.29** | 0.94 |
| sphingosine | LC/MS pos late | 17747 | HMDB00252 | 5353955 | **1.47** | 1.03 | 0.99 | 1.23 | 0.96 | **0.78** | 1.20 | 0.94 | 0.81 |
| hexadecasphingosine (d16:1)* | LC/MS pos late | 57428 |  |  | **1.40** | 1.13 | 1.11 | 1.21 | 1.08 | 0.86 | 1.14 | 1.07 | 0.84 |
| heptadecasphingosine (d17:1) | LC/MS pos late | 57427 |  | 5283557 | **1.88** | 1.36 | 1.03 | 1.28 | 1.08 | 0.74 | 1.08 | 1.07 | **0.67** |
| eicosanoylsphingosine (d20:1)* | LC/MS pos late | 57597 |  |  | 1.02 | 0.91 | 0.99 | 0.98 | 0.84 | 0.97 | 1.02 | 0.96 | 0.91 |
| 3-hydroxy-3-methylglutarate | LC/MS polar | 531 | HMDB00355 | 1662 | 1.01 | 0.94 | 1.02 | 0.96 | 0.94 | **1.18** | 1.06 | 0.92 | **1.18** |
| cholesterol | LC/MS pos late | 63 | HMDB00067 | 11025495 | 0.95 | **1.20** | 0.88 | 1.03 | **1.22** | 1.07 | 1.10 | **1.27** | 1.00 |
| 7-hydroxycholesterol (alpha or beta) | LC/MS pos late | 47890 | HMDB06119 | 107722 | 1.00 | 1.34 | 0.92 | 0.90 | 1.23 | 0.98 | 0.81 | **1.46** | 1.10 |
| AICA ribonucleotide | LC/MS pos early | 38325 | HMDB01517 | 65110 | 0.99 | 1.46 | 0.97 | 0.90 | 1.36 | **0.47** | 0.89 | 1.08 | **0.29** |
| inosine 5'-monophosphate (IMP) | LC/MS pos early | 2133 | HMDB00175 | 8582 | 1.06 | 1.30 | 0.69 | 1.30 | 1.32 | 0.74 | 1.45 | 1.23 | 0.83 |
| inosine | LC/MS pos early | 1123 | HMDB00195 | 6021 | 1.03 | 1.19 | **0.72** | 1.15 | 1.24 | **0.74** | 1.24 | 1.15 | **0.72** |
| hypoxanthine | LC/MS neg | 3127 | HMDB00157 | 790 | 1.10 | 1.12 | **0.65** | 1.12 | 1.10 | **0.63** | 1.20 | 1.12 | **0.65** |
| xanthine | LC/MS neg | 3147 | HMDB00292 | 1188 | 0.93 | 1.09 | **0.76** | 0.95 | 1.07 | **0.75** | 1.07 | 1.12 | **0.73** |
| xanthosine 5'-monophosphate (xmp) | LC/MS neg | 12024 | HMDB01554 | 73323 | 0.95 | 0.87 | 0.88 | 0.65 | 1.36 | 1.16 | 0.74 | 1.33 | 1.06 |
| xanthosine | LC/MS neg | 15136 | HMDB00299 | 64959 | 0.79 | 1.18 | 0.76 | 0.90 | 1.18 | 0.77 | 1.00 | 1.13 | **0.71** |
| 2'-deoxyinosine | LC/MS neg | 15076 | HMDB00071 | 65058 | 1.54 | 1.52 | 0.54 | **2.11** | 2.09 | 0.69 | **2.55** | 2.04 | 0.64 |
| urate | LC/MS neg | 1604 | HMDB00289 | 1175 | **0.68** | 0.84 | 1.31 | **0.65** | 0.82 | 1.24 | **0.74** | 0.92 | 1.02 |
| allantoin | LC/MS polar | 1107 | HMDB00462 | 204 | 0.93 | 1.00 | 1.00 | 1.11 | 1.00 | 1.00 | 1.23 | 1.00 | 1.00 |
| adenosine 5'-diphosphate (ADP) | LC/MS neg | 3108 | HMDB01341 | 6022 | 1.02 | 0.77 | 1.37 | 1.17 | 1.03 | 1.36 | 0.93 | 0.99 | 1.31 |
| adenosine 5'-monophosphate (AMP) | LC/MS pos early | 32342 | HMDB00045 | 6083 | 0.85 | 1.01 | 1.09 | 0.94 | 1.08 | 1.20 | 1.16 | 1.16 | 0.98 |
| adenosine 3'-monophosphate (3'-AMP) | LC/MS neg | 35142 | HMDB03540 | 41211 | 0.85 | 1.04 | 0.87 | 0.94 | 0.97 | 0.83 | 0.75 | 0.93 | 0.85 |
| adenosine 2'-monophosphate (2'-AMP) | LC/MS neg | 36815 | HMDB11617 | 94136 | 1.05 | 1.20 | 0.84 | 1.12 | 1.25 | 0.78 | 1.03 | 1.10 | 0.84 |
| adenosine 3',5'-cyclic monophosphate (cAMP) | LC/MS neg | 2831 | HMDB00058 | 6076 | **1.32** | 1.00 | 1.07 | **1.36** | 1.06 | 1.08 | 1.20 | 1.05 | 1.08 |
| adenylosuccinate | LC/MS neg | 18360 | HMDB00536 | 195 | 1.34 | 0.86 | 0.85 | **2.56** | 1.11 | 0.91 | **3.43** | 1.23 | 0.88 |
| adenosine | LC/MS pos early | 555 | HMDB00050 | 60961 | 0.85 | 0.92 | 0.85 | 0.71 | 1.30 | 0.89 | 1.07 | **1.70** | 1.01 |
| adenine | LC/MS pos early | 554 | HMDB00034 | 190 | 1.69 | 1.12 | 0.64 | 1.12 | 0.96 | 0.61 | 0.98 | 1.04 | 0.58 |
| 1-methyladenine | LC/MS pos early | 1527 | HMDB11599 | 78821 | 0.90 | 0.74 | 1.03 | 0.72 | 0.63 | 1.01 | 0.73 | 0.71 | 1.00 |
| N1-methyladenosine | LC/MS pos early | 15650 | HMDB03331 | 27476 | 1.07 | 1.27 | **0.72** | 0.94 | 1.07 | 0.85 | 1.23 | 1.04 | 0.88 |
| N6-carbamoylthreonyladenosine | LC/MS neg | 35157 | HMDB41623 | 161466 | 1.17 | 1.03 | 0.81 | 1.22 | 1.16 | 0.95 | **1.44** | **1.34** | 0.84 |
| 2'-deoxyadenosine 5'-diphosphate | LC/MS neg | 15116 | HMDB01508 | 188966 | 0.88 | 0.92 | **1.47** | 1.01 | 1.09 | **1.58** | 1.06 | 1.33 | **1.52** |
| 2'-deoxyadenosine 5'-monophosphate | LC/MS neg | 46333 | HMDB00905 | 12599 | 0.94 | 1.01 | 0.94 | 1.16 | **1.28** | 1.01 | **1.56** | **1.49** | 0.97 |
| 2'-deoxyadenosine | LC/MS pos early | 1553 | HMDB00101 | 13730 | 1.03 | 0.73 | 0.51 | 0.93 | 1.36 | **0.45** | 1.53 | 1.77 | 0.62 |
| N6-succinyladenosine | LC/MS neg | 48130 | HMDB00912 | 165243 | 1.45 | 1.29 | 0.74 | **1.81** | **1.77** | 0.83 | **2.40** | **1.83** | 1.02 |
| guanosine 5'- diphosphate (GDP) | LC/MS neg | 2848 | HMDB01201 | 8977 | 0.79 | 1.27 | 0.91 | 0.90 | 0.85 | 0.76 | 0.77 | 1.34 | 1.16 |
| guanosine 5'- monophosphate (5'-GMP) | LC/MS pos early | 2849 | HMDB01397 | 6804 | 0.92 | 0.98 | 0.90 | 0.93 | 1.13 | 0.95 | **1.19** | **1.23** | 0.87 |
| guanosine 3'-monophosphate (3'-GMP) | LC/MS neg | 39786 |  | 3522 | 0.88 | 0.88 | **0.60** | 1.06 | 1.00 | 0.69 | 0.64 | 0.90 | 0.83 |
| guanosine | LC/MS pos early | 1573 | HMDB00133 | 6802 | 1.26 | 1.03 | **0.65** | 1.13 | 0.98 | **0.64** | 1.22 | 0.94 | **0.72** |
| guanine | LC/MS pos early | 32352 | HMDB00132 | 764 | **1.30** | 1.03 | **0.63** | 0.94 | 0.98 | **0.59** | 1.03 | 0.99 | **0.67** |
| 7-methylguanine | LC/MS pos early | 35114 | HMDB00897 | 11361 | 0.90 | 0.92 | 1.01 | 0.78 | 0.88 | 1.00 | **0.73** | 0.90 | 1.04 |
| N2-methylguanosine | LC/MS neg | 35133 | HMDB05862 | 3035422 | 1.02 | 0.97 | 0.92 | 0.94 | 0.96 | **0.83** | 0.88 | 0.86 | 0.98 |
| N2,N2-dimethylguanosine | LC/MS pos early | 35137 | HMDB04824 | 92919 | 0.88 | 0.86 | 0.93 | 0.84 | 0.90 | 0.98 | 0.99 | 0.99 | 0.99 |
| 2'-deoxyguanosine | LC/MS neg | 1411 | HMDB00085 | 187790 | **1.58** | 1.08 | 0.75 | 1.33 | **1.41** | 0.82 | **1.76** | **1.71** | **0.67** |
| dihydroorotate | LC/MS polar | 601 | HMDB03349 | 648 | 1.64 | 1.22 | 0.79 | 1.18 | 0.85 | 0.75 | 0.82 | 0.78 | 0.74 |
| orotate | LC/MS polar | 1505 | HMDB00226 | 967 | 1.26 | 1.44 | 0.76 | 1.19 | 0.89 | **0.59** | 1.06 | 0.86 | **0.49** |
| orotidine | LC/MS polar | 35172 | HMDB00788 | 92751 | 1.09 | 0.96 | 1.06 | 1.00 | 1.05 | **1.15** | **1.15** | 1.08 | **1.19** |
| uridine 5'-triphosphate (UTP) | LC/MS neg | 33448 | HMDB00285 | 6133 | 1.07 | 0.96 | 0.92 | 0.89 | 0.92 | 0.97 | **0.50** | 0.86 | 1.07 |
| uridine 5'-diphosphate (UDP) | LC/MS neg | 5345 | HMDB00295 | 6031 | 0.87 | 0.96 | 1.06 | 0.89 | 0.99 | 1.17 | **0.81** | 1.02 | 1.09 |
| uridine 5'-monophosphate (UMP) | LC/MS polar | 2856 | HMDB00288 | 6030 | 0.94 | 1.04 | 1.02 | 0.99 | 1.19 | 1.14 | **1.39** | **1.36** | 0.94 |
| uridine 3'-monophosphate (3'-UMP) | LC/MS polar | 39764 |  | 101543 | 0.88 | 1.04 | 0.96 | 0.79 | 0.96 | 0.90 | **0.71** | 0.90 | 0.91 |
| uridine | LC/MS neg | 606 | HMDB00296 | 6029 | 1.23 | 1.27 | 0.80 | 1.11 | **1.30** | **0.77** | 1.17 | **1.29** | 0.82 |
| uracil | LC/MS polar | 605 | HMDB00300 | 1174 | 1.10 | 1.19 | **0.48** | 1.43 | 1.14 | 0.59 | 1.63 | 1.03 | 0.65 |
| pseudouridine | LC/MS neg | 33442 | HMDB00767 | 15047 | **0.71** | 0.81 | 1.14 | **0.66** | 0.85 | 1.17 | **0.71** | 0.88 | 1.09 |
| 5,6-dihydrouridine | LC/MS neg | 61833 |  | 94312 | 1.31 | 0.94 | 1.17 | 1.10 | 0.91 | 1.09 | 1.38 | 1.06 | 1.11 |
| 2'-O-methyluridine | LC/MS neg | 57655 |  | 102212 | **0.57** | **0.71** | 1.09 | **0.63** | 0.76 | **1.40** | **0.61** | 0.80 | 1.32 |
| 5-methyluridine (ribothymidine) | LC/MS neg | 35136 | HMDB00884 | 445408 | 1.02 | 0.96 | 1.35 | 0.76 | 0.94 | 1.21 | 0.97 | 1.03 | 1.12 |
| 2'-deoxyuridine | LC/MS neg | 1412 | HMDB00012 | 13712 | **2.29** | **1.74** | **0.60** | **1.74** | 1.24 | **0.51** | 1.45 | 1.26 | 0.63 |
| 3-ureidopropionate | LC/MS pos early | 3155 | HMDB00026 | 111 | 0.97 | 1.17 | 0.91 | 1.04 | 1.23 | 0.85 | 0.90 | 1.19 | 1.25 |
| beta-alanine | LC/MS pos early | 55 | HMDB00056 | 239 | 0.93 | 0.99 | 1.01 | **0.90** | 0.99 | 1.05 | 0.96 | 1.09 | 1.02 |
| cytidine triphosphate | LC/MS neg | 2844 | HMDB00082 | 6176 | 0.88 | 0.83 | 0.83 | **0.64** | 0.73 | 0.94 | **0.38** | **0.63** | 1.08 |
| cytidine diphosphate | LC/MS neg | 2841 | HMDB01546 | 6132 | **0.73** | 0.84 | 1.09 | **0.67** | 0.84 | 1.24 | **0.59** | 0.85 | 1.21 |
| cytidine 5'-monophosphate (5'-CMP) | LC/MS pos early | 2372 | HMDB00095 | 6131 | **0.90** | 0.99 | 0.96 | **0.86** | 1.07 | 1.01 | 0.99 | **1.12** | 0.92 |
| cytidine | LC/MS pos early | 514 | HMDB00089 | 6175 | 1.11 | 1.14 | **0.51** | 1.12 | 1.23 | **0.61** | 1.39 | 1.19 | **0.63** |
| cytosine | LC/MS pos early | 573 | HMDB00630 | 597 | 0.68 | 0.83 | **0.56** | 0.73 | 0.98 | 0.94 | 0.67 | 0.81 | 0.84 |
| 5-methylcytidine | LC/MS pos early | 22119 | HMDB00982 | 92918 | **0.70** | 0.81 | 1.16 | **0.73** | 0.88 | 1.26 | 0.94 | 1.01 | 1.15 |
| 2'-deoxycytidine 5'-monophosphate | LC/MS pos early | 533 | HMDB01202 | 13945 | **0.67** | 0.99 | 1.17 | 1.04 | 1.30 | 1.31 | 1.22 | **1.43** | 1.16 |
| 2'-deoxycytidine | LC/MS pos early | 15949 | HMDB00014 | 13711 | **2.88** | 1.36 | **0.44** | **2.30** | 1.79 | 0.61 | **3.37** | **2.91** | 0.57 |
| 2'-O-methylcytidine | LC/MS pos early | 57554 |  | 150971 | **0.61** | 0.92 | 1.06 | 0.75 | 1.39 | 1.21 | 0.73 | 1.42 | 1.15 |
| thymidine 5'-monophosphate | LC/MS neg | 12023 | HMDB01227 | 9700 | 1.10 | 0.99 | 0.87 | 1.34 | 1.26 | 0.94 | **1.64** | 1.32 | 0.95 |
| 5,6-dihydrothymine | LC/MS neg | 1418 | HMDB00079 | 93556 | 1.22 | 0.83 | 0.66 | 1.17 | 1.33 | 1.24 | 1.46 | **1.52** | 1.07 |
| 3-aminoisobutyrate | LC/MS pos early | 1566 | HMDB03911 | 64956 | 0.58 | 0.81 | 1.55 | 1.12 | 0.62 | 1.27 | 1.08 | **2.12** | 1.65 |
| methylphosphate | LC/MS pos early | 37070 | HMDB61711 | 13130 | **0.76** | 0.88 | 0.93 | 0.77 | 1.02 | 1.00 | 0.90 | 1.21 | 0.97 |
| nicotinate ribonucleoside | LC/MS pos early | 33471 | HMDB06809 | 161234 | **0.43** | 1.56 | 1.12 | 0.85 | 1.56 | 1.21 | 1.21 | **1.92** | 0.99 |
| nicotinic acid mononucleotide (NaMN) | LC/MS pos early | 32461 | HMDB01132 | 5288991 | 0.66 | 1.30 | 1.00 | **0.53** | 1.17 | 1.00 | 0.90 | 0.96 | 1.00 |
| nicotinamide | LC/MS pos early | 594 | HMDB01406 | 936 | 1.03 | 1.04 | 0.92 | 1.00 | 1.09 | 0.99 | 0.99 | 1.10 | 0.96 |
| nicotinamide ribonucleotide (NMN) | LC/MS pos early | 22152 | HMDB00229 | 14180 | 0.93 | 0.98 | **0.74** | 1.00 | 1.01 | 0.79 | 1.10 | 1.02 | **0.70** |
| nicotinamide riboside | LC/MS pos early | 33013 | HMDB00855 | 439924 | 0.83 | 1.11 | 0.92 | 1.04 | 1.04 | 1.02 | 1.28 | 1.04 | 0.80 |
| nicotinamide adenine dinucleotide (NAD+) | LC/MS neg | 5278 | HMDB00902 | 5893 | 0.94 | 0.95 | 1.01 | 0.97 | 0.96 | **1.09** | 1.01 | 1.00 | 1.06 |
| nicotinamide adenine dinucleotide reduced (NADH) | LC/MS neg | 31475 | HMDB01487 | 439153 | 1.27 | 0.95 | 0.74 | 0.99 | 0.96 | **0.65** | 0.95 | 0.96 | 0.73 |
| nicotinamide adenine dinucleotide phosphate reduced (NADPH) | LC/MS neg | 33450 | HMDB00221 | 5884 | **0.52** | 1.22 | 1.70 | 0.95 | 1.35 | **2.05** | 0.99 | 1.56 | **1.86** |
| nicotinate adenine dinucleotide (NAAD+) | LC/MS neg | 15725 |  | 25246170 | 1.06 | 1.00 | 1.01 | 0.95 | 1.03 | 1.02 | 0.90 | 1.00 | 0.97 |
| 1-methylnicotinamide | LC/MS pos early | 27665 | HMDB00699 | 10129985 | **0.90** | 0.98 | **0.91** | **0.87** | **0.87** | 0.96 | **0.84** | **0.82** | **0.89** |
| trigonelline (N'-methylnicotinate) | LC/MS pos early | 32401 | HMDB00875 | 5570 | 0.82 | 0.89 | 1.42 | 0.87 | 0.73 | 1.08 | 0.94 | 0.89 | 0.92 |
| adenosine 5'-diphosphoribose (ADP-ribose) | LC/MS neg | 558 | HMDB01178 | 192 | 1.15 | 1.17 | 0.96 | 1.17 | 1.23 | 1.01 | 0.95 | 1.20 | 1.08 |
| riboflavin (Vitamin B2) | LC/MS pos early | 1827 | HMDB00244 | 493570 | 1.11 | 1.37 | **0.58** | 0.77 | 1.09 | **0.57** | 1.12 | 0.92 | **0.55** |
| flavin adenine dinucleotide (FAD) | LC/MS neg | 2134 | HMDB01248 | 643975 | 0.97 | 0.98 | 0.95 | 0.92 | 0.98 | 0.97 | 0.92 | 1.00 | 0.95 |
| flavin mononucleotide (FMN) | LC/MS neg | 15797 | HMDB01520 | 710 | **0.77** | 1.15 | 0.97 | 0.85 | 1.15 | 1.04 | 0.98 | **1.35** | 0.95 |
| pantothenate | LC/MS pos early | 1508 | HMDB00210 | 6613 | 0.88 | 0.93 | 1.09 | 1.13 | 0.96 | **1.31** | 0.97 | 1.07 | **1.31** |
| phosphopantetheine | LC/MS neg | 15504 | HMDB01416 | 987 | 1.24 | 1.02 | 0.59 | 0.63 | 0.87 | **0.36** | 0.64 | 0.97 | 0.61 |
| 3'-dephosphocoenzyme A | LC/MS neg | 18289 | HMDB01373 | 444485 | 1.16 | 1.25 | 0.72 | 1.31 | 1.13 | 0.83 | **2.69** | 1.19 | 0.89 |
| coenzyme A | LC/MS neg | 46322 | HMDB01423 | 317 | **0.57** | 1.08 | 0.83 | 1.07 | 1.27 | 1.25 | 1.24 | 1.33 | 1.11 |
| pantetheine | LC/MS neg | 57555 |  | 439322 | 1.06 | 0.88 | 0.96 | 0.85 | 0.87 | 0.79 | 0.87 | 0.85 | 0.91 |
| gulonate* | LC/MS polar | 46957 | HMDB03290 | 9794176 | 1.07 | 0.91 | 1.10 | 0.98 | **1.16** | **1.35** | **1.17** | 1.11 | **1.36** |
| alpha-tocopherol | LC/MS pos late | 1561 | HMDB01893 | 14985 | **1.22** | 1.14 | 1.15 | 1.02 | 1.01 | 1.01 | 1.18 | **1.24** | 1.11 |
| 5-methyltetrahydrofolate (5MeTHF) | LC/MS neg | 18330 | HMDB01396 | 146 | 1.10 | 0.88 | **1.26** | **1.35** | 0.98 | **1.26** | **1.19** | 1.02 | **1.24** |
| pterin | LC/MS pos early | 43023 | HMDB00802 | 73000 | **2.25** | 0.83 | 0.85 | **2.39** | **2.00** | **2.42** | **2.63** | **2.84** | **3.83** |
| bilirubin (Z,Z) | LC/MS pos late | 43807 | HMDB00054 | 5280352 | 1.15 | 1.02 | 1.14 | 1.09 | 1.28 | **1.50** | **1.68** | **1.77** | **1.86** |
| thiamin (Vitamin B1) | LC/MS pos early | 5341 | HMDB00235 | 1130 | 1.03 | 1.09 | **0.82** | 0.97 | 0.96 | 0.87 | 0.95 | 0.91 | **0.83** |
| thiamin diphosphate | LC/MS neg | 35670 | HMDB01372 | 1132 | 0.95 | 0.85 | 1.31 | 0.91 | 0.88 | 1.25 | 0.99 | 0.78 | 1.49 |
| retinol (Vitamin A) | LC/MS pos late | 1806 | HMDB00305 | 445354 | **2.87** | 0.95 | 1.07 | 0.92 | 0.76 | 0.57 | 0.81 | 0.76 | 0.71 |
| pyridoxine (Vitamin B6) | LC/MS neg | 608 | HMDB02075 | 1054 | 1.27 | 1.47 | 0.57 | 1.23 | 1.12 | 0.55 | 1.06 | 0.66 | **0.38** |
| pyridoxamine | LC/MS pos early | 2150 | HMDB01431 | 1052 | **1.65** | 1.21 | 0.76 | **1.48** | 1.30 | 1.00 | **1.51** | 1.30 | 0.88 |
| pyridoxal phosphate | LC/MS neg | 5331 | HMDB01491 | 1051 | 0.94 | 1.13 | **0.79** | 1.07 | 1.18 | 1.06 | 1.10 | 0.94 | 1.10 |
| pyridoxal | LC/MS pos early | 1651 | HMDB01545 | 1050 | 1.14 | 1.10 | **0.76** | 1.14 | 1.21 | 0.88 | **1.30** | **1.27** | 0.90 |
| benzoate | LC/MS neg | 15778 | HMDB01870 | 243 | 0.96 | **2.18** | 1.53 | 0.97 | 1.77 | 1.00 | 0.80 | 1.09 | 1.76 |
| guaiacol sulfate | LC/MS neg | 46111 | HMDB60013 | 22473 | 0.93 | **1.33** | 1.18 | 1.07 | **1.47** | 1.00 | 1.05 | 1.21 | 1.09 |
| p-cresol sulfate | LC/MS neg | 36103 | HMDB11635 | 4615423 | 0.92 | 0.95 | 1.09 | 0.82 | 1.06 | 1.04 | 0.86 | **1.34** | 0.98 |
| gluconate | LC/MS polar | 587 | HMDB00625 | 10690 | 1.66 | 1.33 | 0.79 | 1.28 | 1.16 | 0.87 | 1.25 | 0.98 | 0.97 |
| beta-guanidinopropanoate | LC/MS pos early | 35101 | HMDB13222 | 67701 | 1.05 | **2.00** | 1.86 | 1.32 | 1.40 | 1.34 | 0.95 | 1.37 | 1.48 |
| ergothioneine | LC/MS pos early | 37459 | HMDB03045 | 3032311 | 1.43 | 1.16 | 1.58 | 1.48 | 1.05 | 1.00 | 1.60 | 1.18 | 1.00 |
| homocitrate | LC/MS polar | 39601 | HMDB03518 | 439459 | 1.02 | 0.93 | 1.12 | 0.83 | 0.99 | 1.13 | 0.96 | 1.17 | 1.07 |
| N-glycolylneuraminate | LC/MS pos early | 37123 | HMDB00833 | 123802 | 1.11 | 0.84 | 1.17 | 0.91 | 0.84 | 1.03 | 0.96 | 0.93 | 1.09 |
| sulfate* | LC/MS neg | 46960 | HMDB01448 | 1118 | 0.92 | 0.92 | 0.96 | **0.80** | 0.86 | 1.02 | **0.80** | **0.84** | 1.02 |
| O-sulfo-L-tyrosine | LC/MS neg | 45413 |  | 514186 | 0.92 | 0.91 | 1.06 | 1.13 | 0.92 | 1.10 | 1.05 | 0.96 | 1.13 |
| 2-aminophenol sulfate | LC/MS neg | 43266 | HMDB61116 | 181670 | 0.95 | 1.05 | 1.00 | **0.87** | 1.05 | 1.00 | 0.96 | 0.99 | 0.94 |
| phenol red | LC/MS neg | 36817 |  | 4766 | 0.90 | 0.93 | 0.97 | 0.86 | 0.84 | 1.04 | **0.76** | **0.65** | 1.04 |
| succinimide | LC/MS polar | 41888 |  | 11439 | 0.70 | 0.97 | 1.16 | 1.11 | 1.19 | **2.05** | 1.21 | 1.12 | **1.78** |
| trizma acetate | LC/MS pos early | 20710 |  | 6503 | **0.68** | 0.82 | 0.81 | **0.64** | 0.90 | 1.01 | **0.45** | 0.82 | 1.00 |
| thioproline | LC/MS pos early | 53231 |  | 93176 | 1.08 | 1.04 | 0.80 | **0.78** | 1.01 | 0.99 | 0.82 | 1.06 | 0.82 |
