# Supplementary material for: Cooperative CCL2/CCR2 and HGF/MET signaling enhances breast cancer growth and invasion associated with metabolic reprogramming
Source: Cancer Biol Ther. 2025 Jul 30;26(1):2535824. doi: 10.1080/15384047.2025.2535824 (PMC12320856; doi:10.1080/15384047.2025.2535824)
Supplement: Supp figure 4 4 25.pdf [file KCBT_A_2535824_SM7714.pdf]

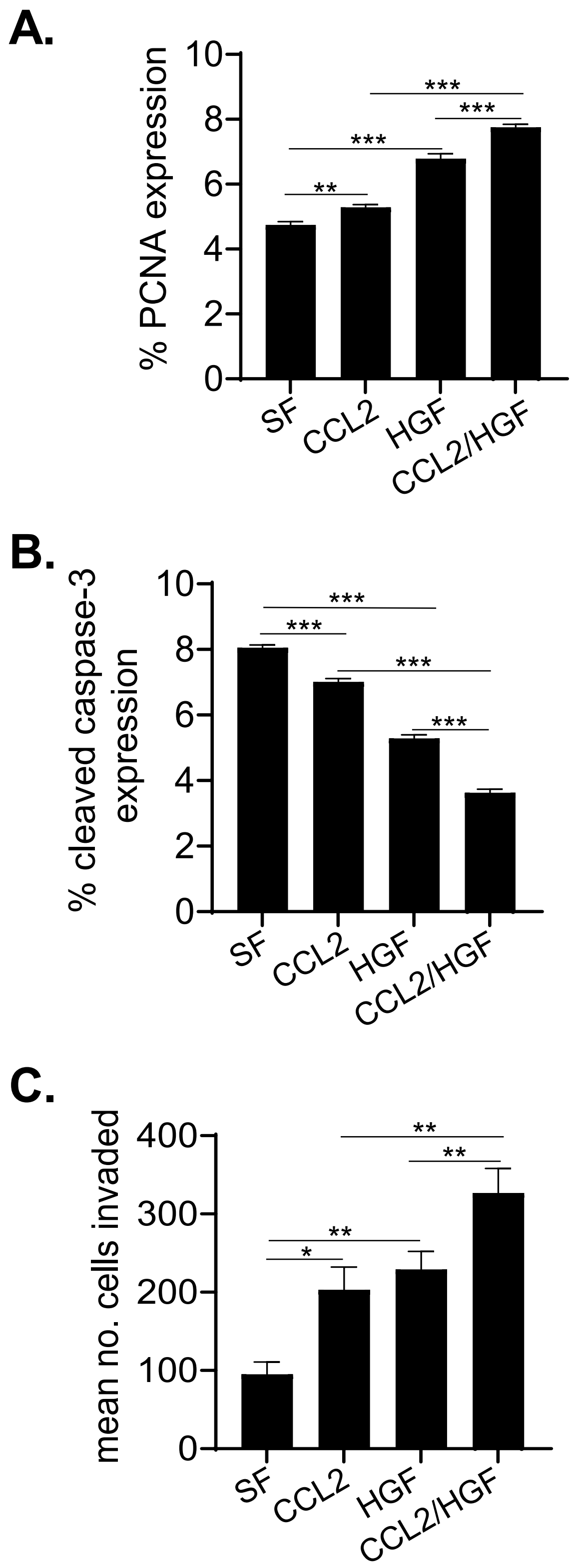

Supplemental Figure 1. Effects of CCL2 and HGF on cell proliferation, survival and invasion in HCC1937 cells. HCC1937 breast cancer cells were treated with/without 100 ng/ml CCL2 and/or HGF for 24 hours and analyzed for **A.** proliferation by PCNA immunostaining**, B.** apoptosis by cleaved caspase-3 immunostaining or **C.** invasion by transwell well assay. Expression was quantified by Image J. Statistical analysis was performed using One Way ANOVA with Tukey's post-hoc test. Statistical significance was determined by $p<0.05$. *$p<.05$, **$p<0.01$, ***$p<0.001$.

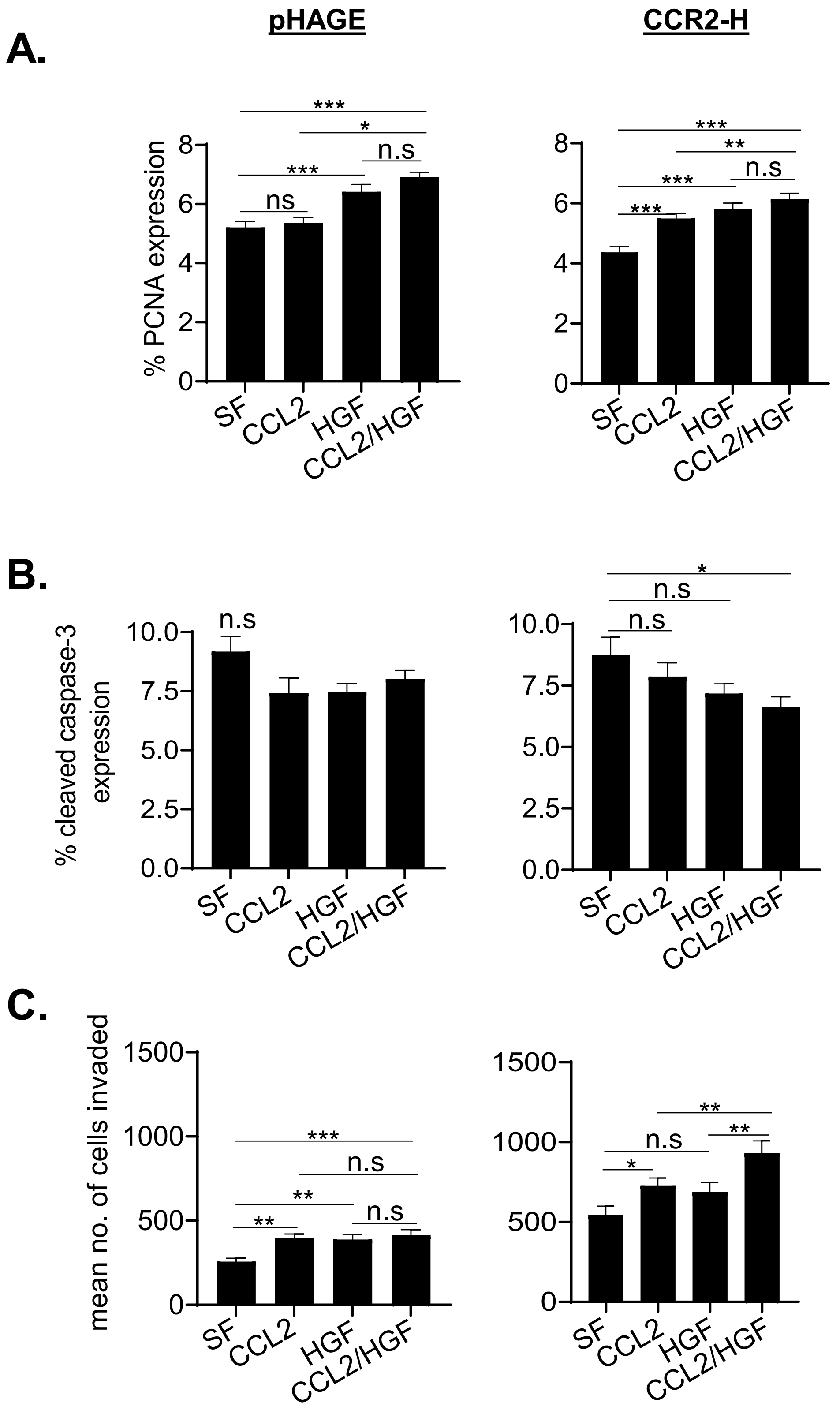

Supplemental Figure 2. CCR2 overexpression in SUM225 cells modulates responsiveness to CCL2 and HGF. SUM225 cells overexpressing CCR2 (CCR2-H) or pHAGE control cells were treated with 100 ng/ml CCL2 or HGF for 24 hours and analyzed for changes in **A.** cell proliferation by PCNA immunostaining, **B.** apoptosis by immunostaining for cleaved caspase-3 or **C**. invasion by transwell assay. Expression was quantified by Image J. Statistical analysis was performed using One Way ANOVA with Tukey's post-hoc test. Statistical significance was determined by $p<0.05$. *$p<.05$, **$p<0.01$, ***$p<0.001$, n.s=not significant.

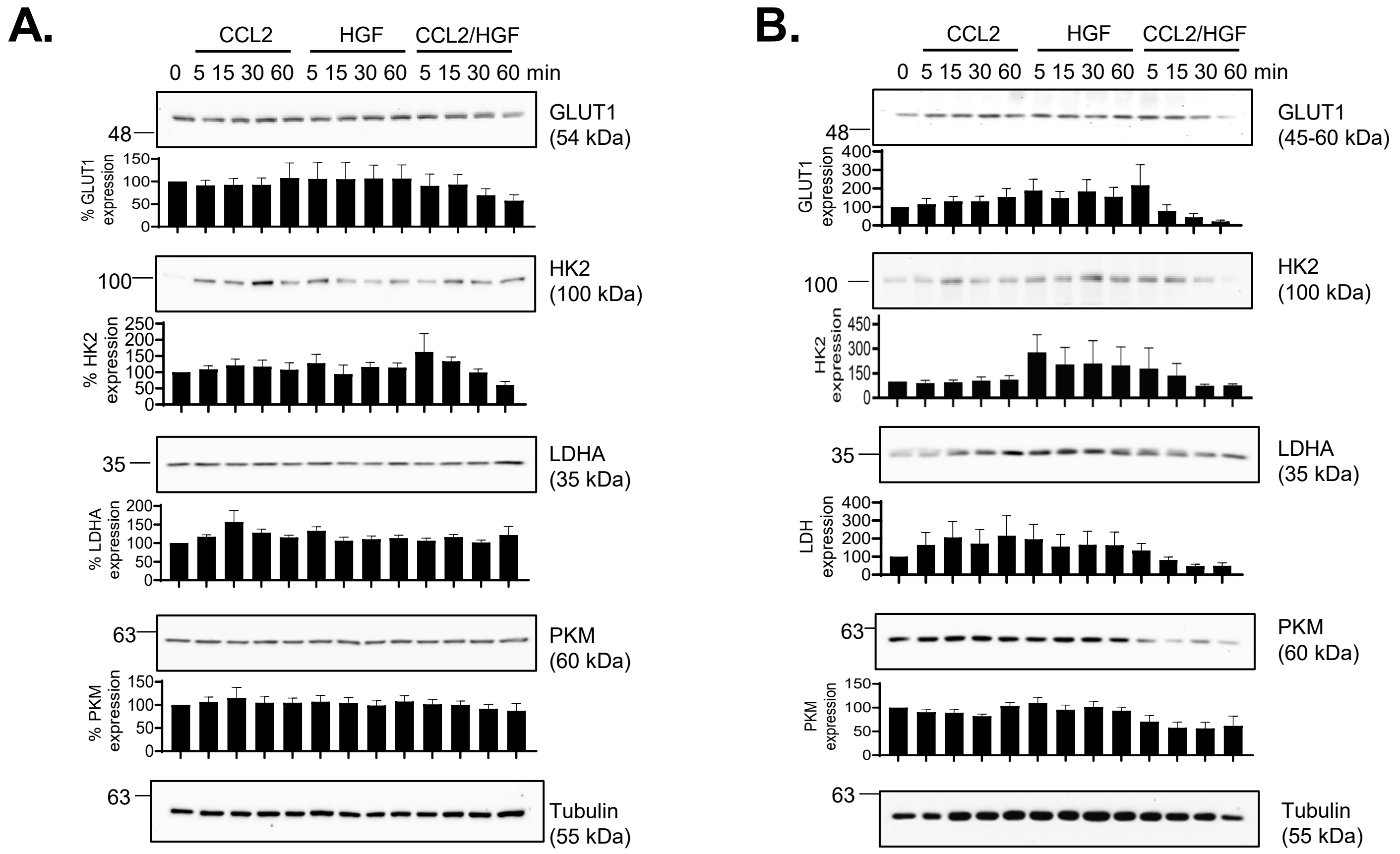

Supplemental Figure 3. CCL2 and HGF co-treatment modulate glycolytic protein expression in breast cancer cell lines. A. DCIS.com or **B.** HCC1937 cells were treated with/without 100 ng/ml CCL2 and/or HGF for up to 60 minutes and immunoblotted for expression of the indicated proteins. Densitometry analysis was performed using Image J. Mean+SEM are shown for 6 experiments.

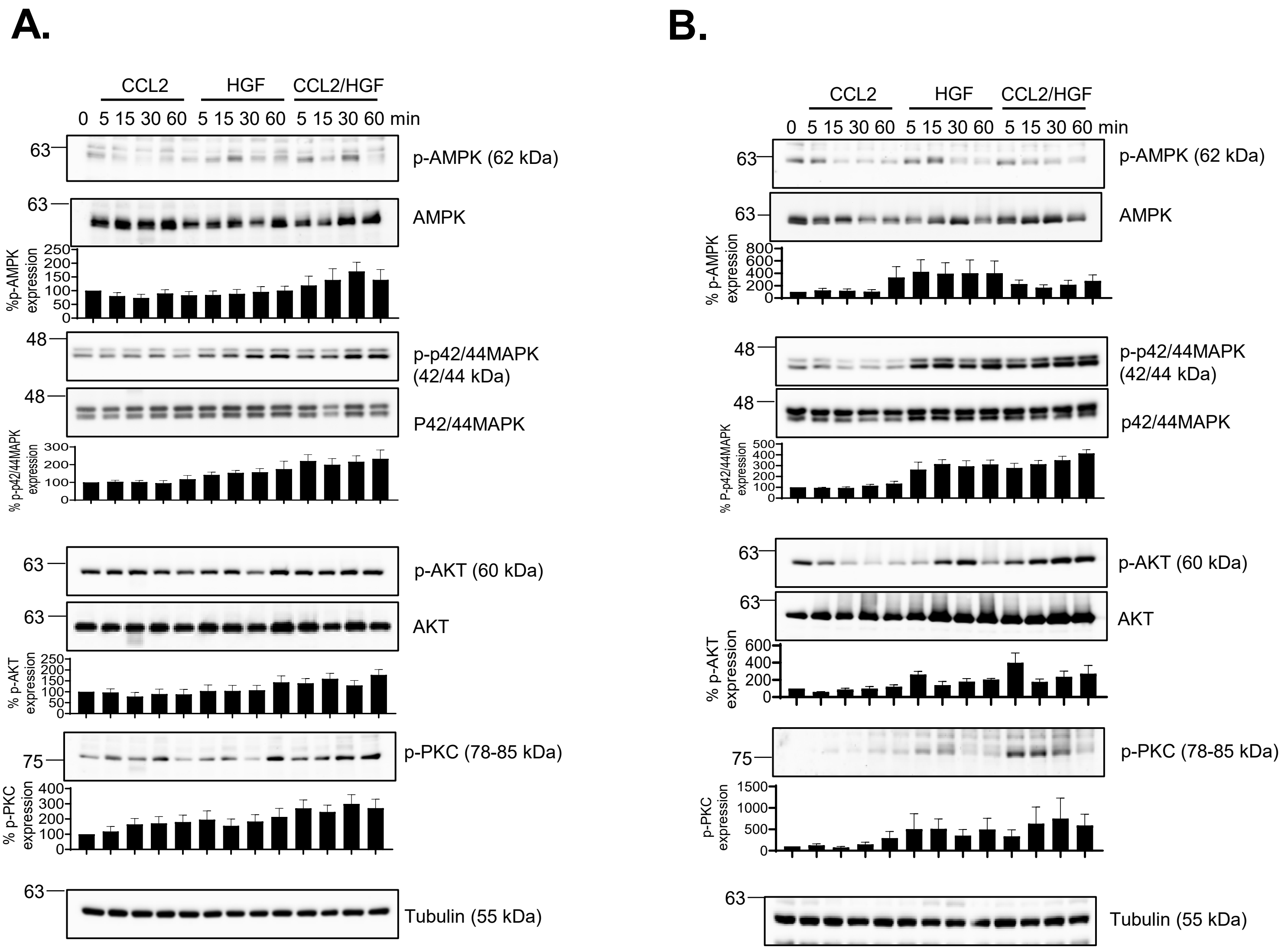

Supplemental figure 4. CCL2 and HGF co-treatment enhances phosphorylation of AKT, AMPK, p42/44MAPK and PKC in breast cancer cells. A. DCIS.com or **B.** HCC1937 cells were treated with 100 ng/ml CCL2 and/or HGF for up to 60 minutes and analyzed for expression of the indicated proteins by immunoblot. Expression was quantified by densitometry. Mean+SEM are shown for 6 experiments for DCIS.com cells and 4 experiments for HCC1937 cells.

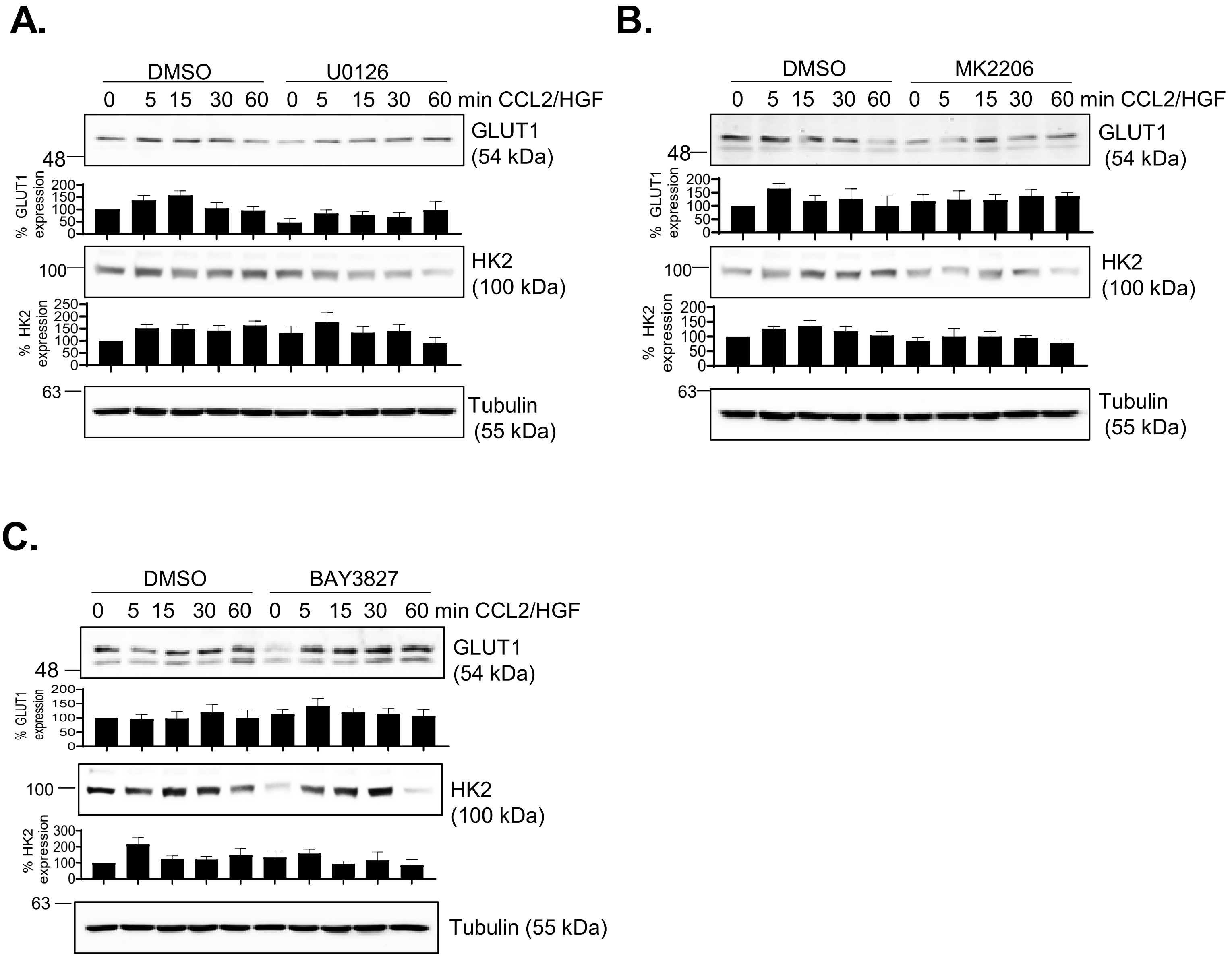

Supplemental Figure 5. Effects of pharmacologic inhibition of AKT, AMPK, PKC and p42/44MAPK on CCL2/HGF mediated expression of glycolytic protein expression. DCIS.com cells were treated with 100 ng/ml CCL2 and HGF with/without or inhibitors to **A.** p42/44MAPK (1 μM U0126) **B.** AKT (1 mM MK2206) or **C.** AMPK (5 nM BAY3827), for up to 60 minutes and analyzed for expression the indicated proteins by immunoblot. Protein expression was quantified by densitometry. Mean+SEM are shown for 6 experiments.

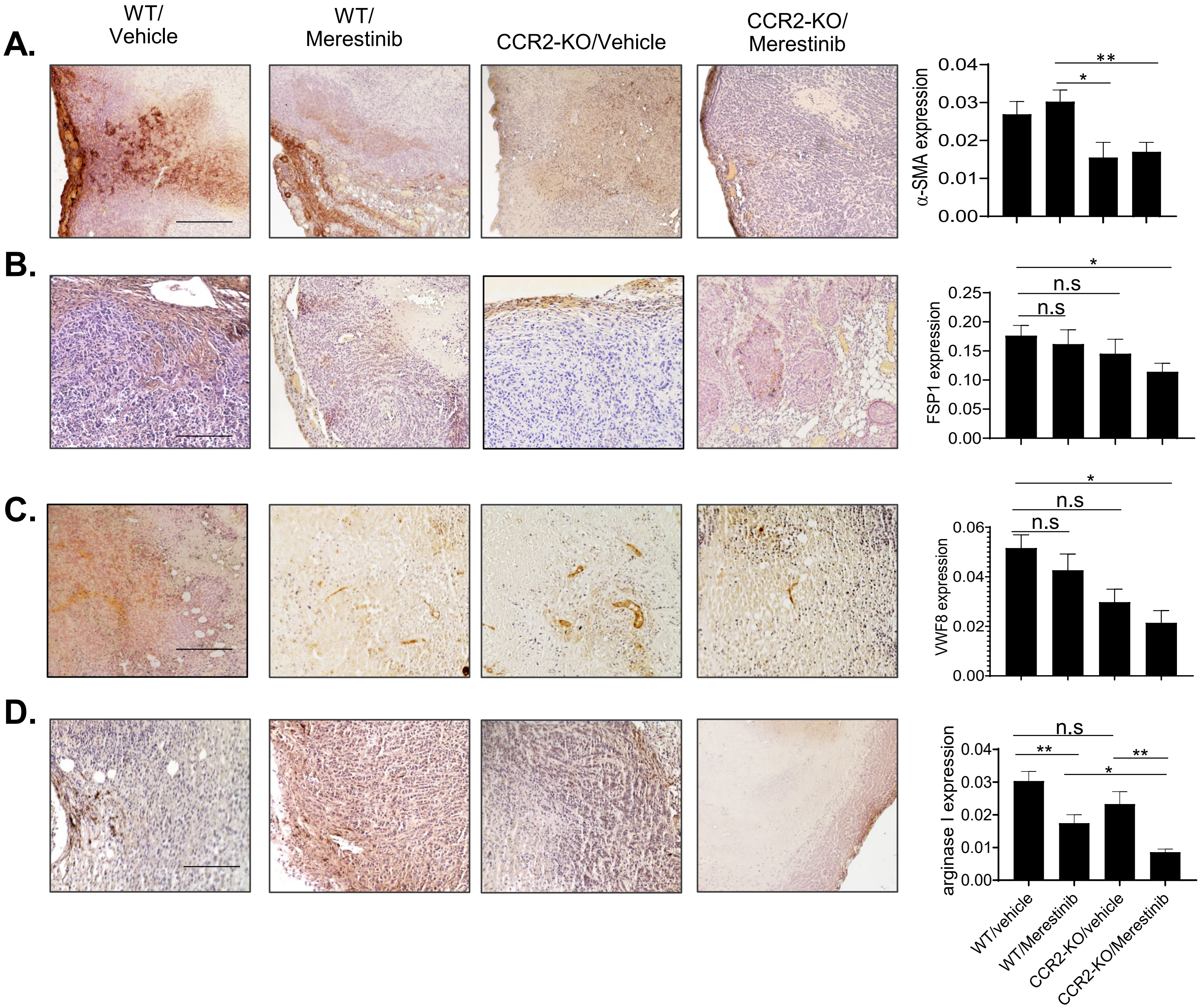

Supplemental Figure 6. Merestinib and CCR2-KO inhibit stromal reactivity. NSG mice bearing DCIS.com xenografts with/without CCR2 CRISPR KO were treated with 12 mg/kg Merestinib or 20% Captisol vehicle control for 4 weeks. **A.-D.** Mammary tissues were immunostained for expression of alpha smooth muscle actin (a-SMA) (A), Fibroblast Specific protein 1 (FSP1) (B), VWF8 (C) or arginase I (D). Expression was quantified by Image J. Statistical analysis was determined by One Way ANOVA with Tukey post hoc comparison. Statistical significance was determined by $p<0.05$. *$p<0.05$. **$p<0.01$. n.s=not significant. Mean±SEM are shown. Scale bar=200 microns.
